# Early DNA methylation changes in children developing beta cell autoimmunity at a young age

**DOI:** 10.1007/s00125-022-05657-x

**Published:** 2022-02-10

**Authors:** Inna Starskaia, Essi Laajala, Toni Grönroos, Taina Härkönen, Sini Junttila, Roosa Kattelus, Henna Kallionpää, Asta Laiho, Veronika Suni, Vallo Tillmann, Riikka Lund, Laura L. Elo, Harri Lähdesmäki, Mikael Knip, Ubaid Ullah Kalim, Riitta Lahesmaa

**Affiliations:** 1 Turku Bioscience Centre, University of Turku and Åbo Akademi University, Turku, Finland; 2 InFLAMES Research Flagship Center, University of Turku, Turku, Finland; 3 Turku Doctoral Programme of Molecular Medicine, University of Turku, Turku, Finland; 4 Department of Computer Science, Aalto University, Espoo, Finland; 5 Pediatric Research Center, Children’s Hospital, University of Helsinki, and Helsinki University Hospital, Helsinki, Finland; 6 Research Program for Clinical and Molecular Metabolism, Faculty of Medicine, University of Helsinki, Helsinki, Finland; 7 Children’s Clinic of Tartu University Hospital, Tartu, Estonia; 8 Institute of Clinical Medicine, University of Tartu, Tartu, Estonia; 9 Institute of Biomedicine, University of Turku, Turku, Finland; 10 Tampere Center for Child Health Research, Tampere University Hospital, Tampere, Finland

**Keywords:** DNA methylation, Epigenetics, T cells, Type 1 diabetes

## Abstract

**Aims/hypothesis:**

Type 1 diabetes is a chronic autoimmune disease of complex aetiology, including a potential role for epigenetic regulation. Previous epigenomic studies focused mainly on clinically diagnosed individuals. The aim of the study was to assess early DNA methylation changes associated with type 1 diabetes already before the diagnosis or even before the appearance of autoantibodies.

**Methods:**

Reduced representation bisulphite sequencing (RRBS) was applied to study DNA methylation in purified CD4^+^ T cell, CD8^+^ T cell and CD4^−^CD8^−^ cell fractions of 226 peripheral blood mononuclear cell samples longitudinally collected from seven type 1 diabetes-specific autoantibody-positive individuals and control individuals matched for age, sex, HLA risk and place of birth. We also explored correlations between DNA methylation and gene expression using RNA sequencing data from the same samples. Technical validation of RRBS results was performed using pyrosequencing.

**Results:**

We identified 79, 56 and 45 differentially methylated regions in CD4^+^ T cells, CD8^+^ T cells and CD4^−^CD8^−^ cell fractions, respectively, between type 1 diabetes-specific autoantibody-positive individuals and control participants. The analysis of pre-seroconversion samples identified DNA methylation signatures at the very early stage of disease, including differential methylation at the promoter of *IRF5* in CD4^+^ T cells. Further, we validated RRBS results using pyrosequencing at the following CpG sites: chr19:18118304 in the promoter of *ARRDC2*; chr21:47307815 in the intron of *PCBP3*; and chr14:81128398 in the intergenic region near *TRAF3* in CD4^+^ T cells.

**Conclusions/interpretation:**

These preliminary results provide novel insights into cell type-specific differential epigenetic regulation of genes, which may contribute to type 1 diabetes pathogenesis at the very early stage of disease development. Should these findings be validated, they may serve as a potential signature useful for disease prediction and management.

**Graphical abstract:**

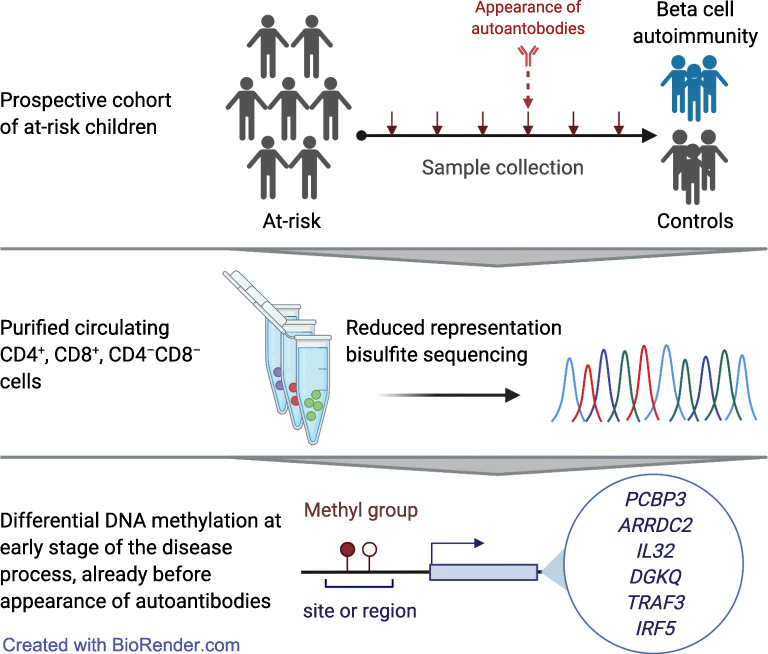

**Supplementary Information:**

The online version contains peer-reviewed but unedited supplementary material available at 10.1007/s00125-022-05657-x.

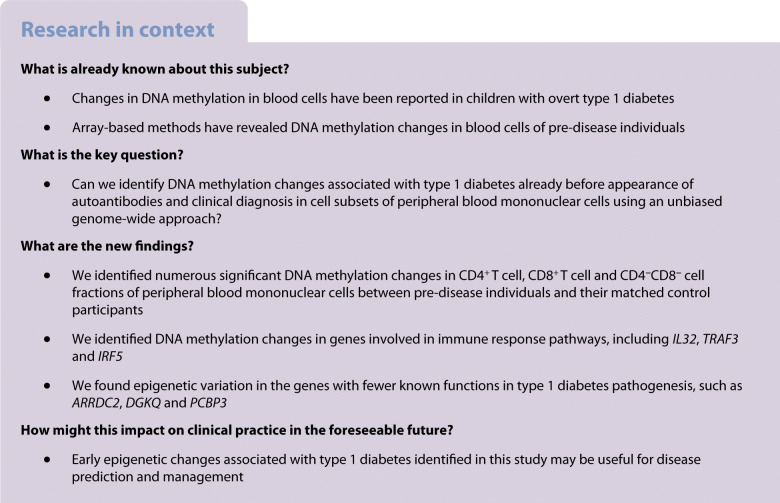



## Introduction

Type 1 diabetes is a complex autoimmune disease with a strong genetic component. Genome-wide association studies (GWAS) have identified more than 60 genetic loci associated with the risk of type 1 diabetes [1–3]. However, genetic variation alone cannot explain the conspicuous rise in the disease incidence during the past decades [4]. Several environmental factors, including viral infections, diet, toxins and other determinants, have been implicated [5].

Environmental exposures may result in epigenetic modifications of DNA, chromatin and histone proteins that change the level of gene expression. The importance of epigenetic gene regulation in complex disease phenotypes has been highlighted for several autoimmune disorders, such as rheumatoid arthritis (RA) and inflammatory bowel disease (IBD) [6, 7]. DNA methylation is one of the most well-studied and key epigenetic modifications. It represents the addition of a methyl group to cytosine usually at the CpG dinucleotide and is typically associated with gene expression silencing [8].

A few studies identified a link between DNA methylation in circulating immune cells and type 1 diabetes [9–13]. However, most of those studies focused on individuals who had already developed clinical type 1 diabetes. A recent study examined DNA methylation changes in peripheral blood samples longitudinally collected before the diagnosis of type 1 diabetes from children enrolled in the prospective Diabetes Autoimmunity Study in the Young (DAISY) cohort [14]. However, the study was limited by the use of a 450 K array for methylation profiling that restricts the analysis to approximately 450,000 targeted CpG sites.

The detection of type 1 diabetes-associated autoantibodies targeting insulin (IAA), glutamic acid decarboxylase (GADA), tyrosine phosphatase-like protein (islet antigen-2, IA-2A) and zinc transporter-8 (ZnT8A) is currently the primary method to predict the development of type 1 diabetes. Children who develop multiple autoantibodies are very likely to progress to type 1 diabetes [15]. However, the appearance of autoantibodies is an indication of an already ongoing autoimmune reaction. Therefore, discovery of methylation changes at the early stages of disease onset, before seroconversion, could provide new insights into molecular mechanisms leading to type 1 diabetes.

The aim of this study was to assess early DNA methylation changes associated with type 1 diabetes in longitudinally collected samples from children who later developed islet autoimmunity at a young age. We used the reduced representation bisulphite sequencing (RRBS) approach instead of array-based methods to achieve broader coverage including sites that are not included in the arrays. Further, we purified the CD4^+^ T cell, CD8^+^ T cell and CD4^−^CD8^−^ cell fractions of peripheral blood mononuclear cells (PBMCs) to determine cell type-specific methylation differences that have not been reported earlier. Besides identifying type 1 diabetes-associated DNA methylation changes, we investigated correlations between DNA methylation and gene expression, exploiting our earlier RNA sequencing (RNA-seq) study of the same samples [16]. The identification of early DNA methylation changes associated with type 1 diabetes will provide novel insights into the pathogenesis of the disease, and may serve as a potential signature for disease prediction and management.

## Methods

### Study cohort

**Fig. 1:**
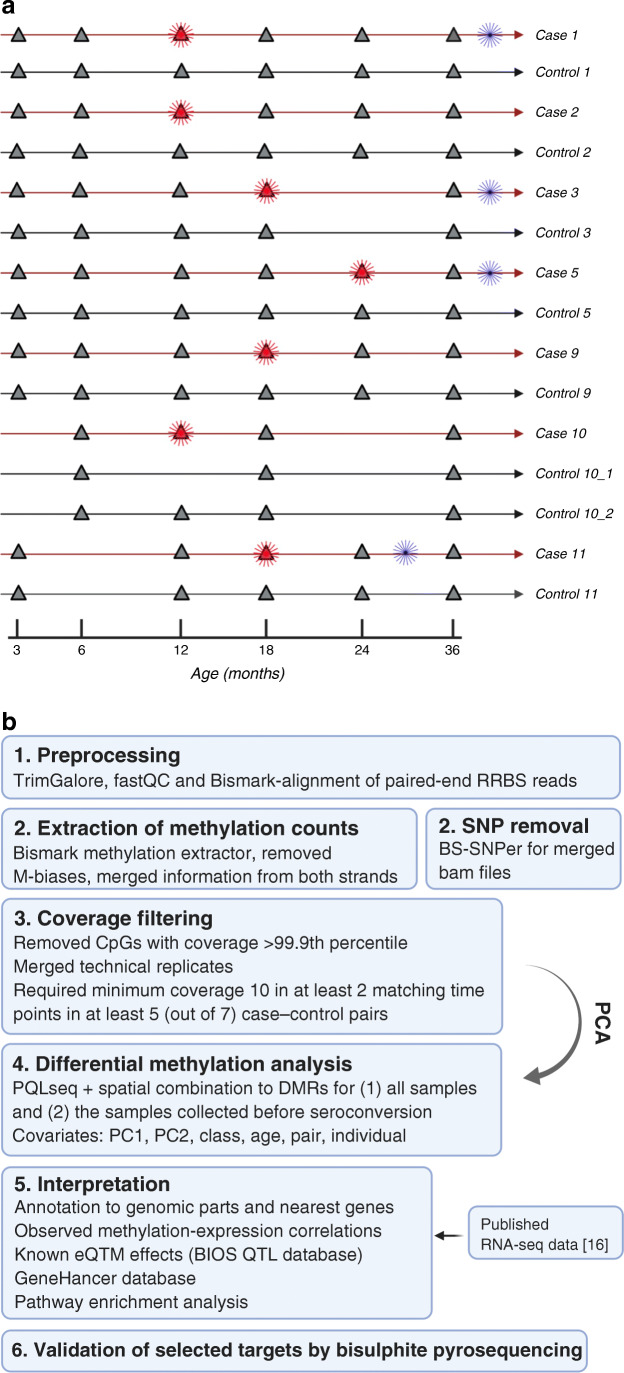
Study design and analysis workflow schematics. (**a**) Study design. Each case individual is visualised as a red line above the corresponding control individual, visualised as a black line. The sample collection time points are marked as triangles. Diagnosis of type 1 diabetes and seroconversion are marked as blue and red asterisks, respectively. (**b**) The schematic shows the outline of the RRBS data analysis workflow. Both (**a**) and (**b**) were created with BioRender. M-biases, biased average methylation level at 5′ or 3′ of the reads; PC1, principal component 1; PC2, principal component 2; PCA, principal component analysis

The cohort analysed here using RRBS is described in an earlier report on transcriptomics analysis of the same individuals [16]. DNA samples were longitudinally collected from seven case–control pairs of the Pathogenesis of Type 1 Diabetes – Testing the Hygiene Hypothesis (DIABIMMUNE) cohort (Fig. 1a, electronic supplementary material [ESM] Table 1). In total, we analysed 226 samples: 73, 77 and 76 for CD4^+^ T cells, CD8^+^ T cells and CD4^−^CD8^−^ cell fractions, respectively. The study protocols were approved by the ethics committees of the participating hospitals, and the parents gave written informed consent. HLA genotyping was described earlier [16]. Autoantibodies IAA, GADA, IA-2A and ZnT8A were measured from serum with specific radiobinding assays. The cutoff values were based on the 99th percentiles in children without diabetes, which were 2.80 relative units (RU) for IAA, 5.36 RU for GADA, 0.78 RU for IA-2A and 0.61 RU for ZnT8A. A sample was considered seropositive when any of the autoantibodies exceeded the threshold. The case individuals became positive for at least two type 1 diabetes-specific autoantibodies at the age of 1–2 years, whereas the control individuals remained autoantibody-negative throughout the follow-up period. The case–control pairs were matched by sex, place of birth, age and HLA risk class. The age was matched ±2 months except for the 36 months samples, where the samples were collected within 4 months. The HLA risk classes were defined as described earlier [17]. A detailed description of the study participants and their antibody measurements is presented in ESM Table 2.

### Sample fractionation and DNA extraction

PBMC sample collection, fractionation and viability of cells were as described earlier [16]. DNA was extracted from CD4^+^ T cells, CD8^+^ T cells and CD4^−^CD8^−^ cell fractions with Qiagen’s AllPrep Universal kit (Qiagen, Hilden, Germany, cat. No. 80224), according to the AllPrep DNA/RNA/miRNA Universal Handbook, 09/2012.

### RRBS library preparation

The library preparation from 100 ng of genomic DNA was carried out according to a protocol adapted from a gel-free multiplexed RRBS method [18]. Subsequent steps were performed as described earlier [19].

### HiSeq 2500 sequencing

The samples were normalised and pooled for the automated cluster preparation with an Illumina cBot station (Illumina, San Diego, CA, USA). CD4^+^ T cell libraries were run on eight lanes, 8–9 samples per lane. The CD8^+^ T cell libraries and CD4^−^CD8^−^ cell fraction libraries were allocated to four pools, including 18–20 samples per pool. Each pool was run on three lanes. The samples were sequenced with an Illumina HiSeq 2500 instrument (Illumina) and TruSeq v3 sequencing chemistry (Illumina). Paired-end sequencing with 2 × 100-bp read length was used with a 6-bp index run. Technical quality of the HiSeq 2500 run was good, and the cluster amount was as expected. More than 76% of all bases above Illumina quality score Q30 was required. The sequencing runs of 37 samples were repeated due to low raw read counts (less than 30 million reads per sample). The median yield of the other samples was 47 million reads (read 1 + read 2).

### RRBS data analysis

**Fig. 2:**
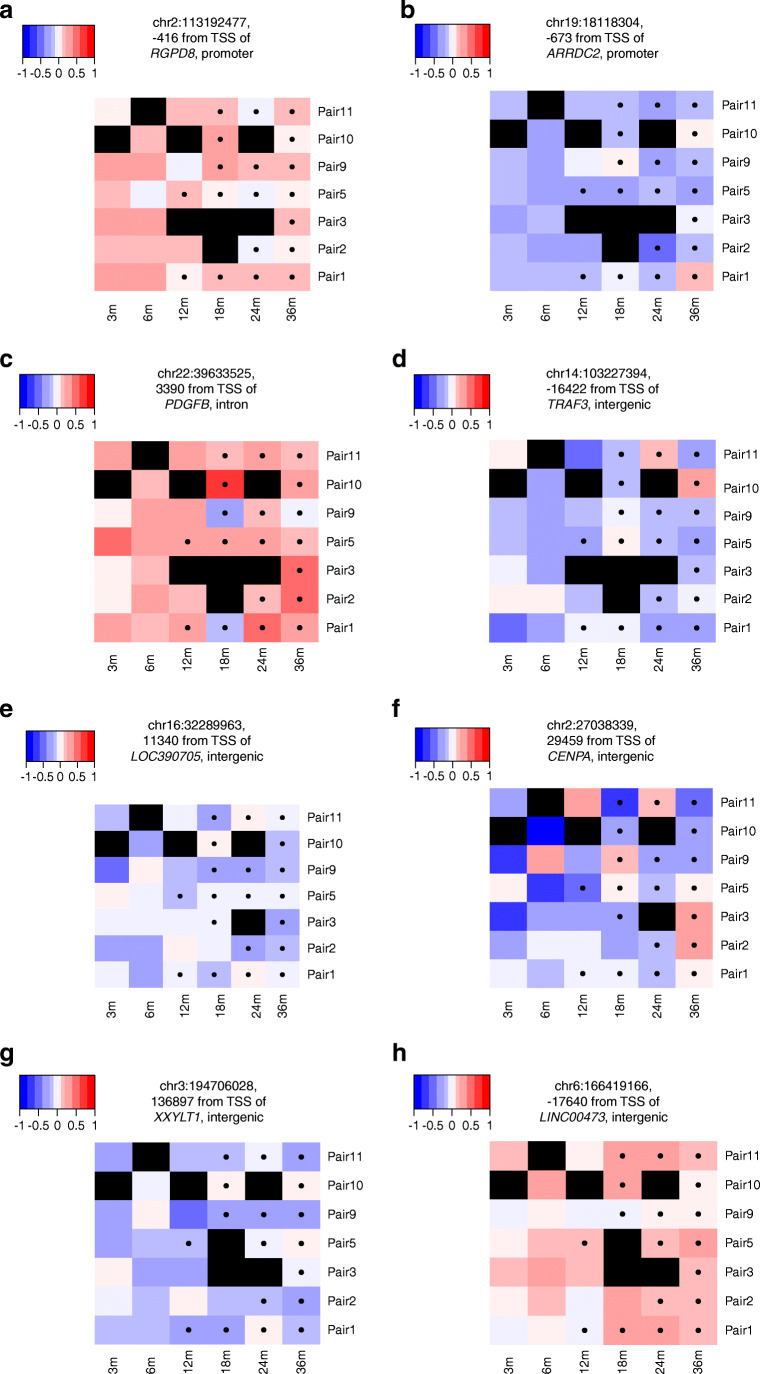
DMCs. Methylation profiles of top-ranked DMCs between cases and controls identified in CD4^+^ T cells (**a**–**d**), CD8^+^ T cells (**e**, **f**) and CD4^−^CD8^−^ fractions (**g**, **h**). The plots are coloured according to the pairwise methylation difference, as specified in the colour key. Blue and red indicate hypo- and hypermethylation of the CpG site in cases, compared with controls, respectively. Black represents missing values. The pairs of individuals are not in any particular order. A black dot in the boxes indicates a sample collected after seroconversion. m, months

RRBS data analysis was performed as we described previously [20]. The data analysis workflow is summarised in Fig. 1b. More detailed description is also provided in the ESM Methods. Most of the data analysis was done with R versions 3.6.1 and 4.0.4 [21]. The heatmaps in Fig. 2 were produced with the R package hamlet [22]. Annotation of differentially methylated CpG sites (DMCs) to genomic regions was carried out through R package genomation version 1.16.0 [23] using Genome Reference Consortium Human Build 37 (GRCh37/hg19).

### Expression quantitative trait methylation analysis

A list of human whole blood *cis*-expression quantitative trait methylation (*cis*-eQTM, false discovery rate-corrected *p* value [FDR] <0.1) effects was downloaded from the BIOS QTL database [24] (accessed 1 April 2020), which is based on 3841 Dutch peripheral blood samples.

### Methylation–expression correlation analysis

We utilised our published RNA-seq data from the same samples to calculate methylation–expression correlations. Spearman correlations across all samples within each cell fraction were calculated between each DMC and all genes with transcription start sites (TSSs) within a window of 250 kb in both directions from the CpG site’s genomic location. The Ensembl identifiers and gene names are from Ensembl versions 70 [25] and 75 [26].

### Pathway enrichment analysis

Gene Ontology (GO) terms were used in the pathway enrichment analysis. Only DMCs with adjusted *p* value (Benjamini–Hochberg-corrected *p* value) <0.1 and their corresponding nearest and correlating genes were used for the analysis. Pathways were considered to be significantly enriched at FDR <0.05 (Fisher’s exact test).

### Pyrosequencing

PyroMark assay design 2.0 software (Qiagen) was used to design the assays. Target-specific primers designed with the software include a primer pair with site-specific biotinylation for target amplification and a pyrosequencing primer. Sample preparations started with 200 ng of DNA from three selected case–control pairs. Samples were first sodium bisulphite-treated with EZ DNA Methylation-Gold Kit (Zymo Research, Irvine, CA, USA, cat. No. D5006). Selected targets were then amplified by PyroMark PCR Kit (Qiagen, cat. No. 978703). A biotin-labelled template strand was then used for the pyrosequencing reaction with a specific primer using PyroMark Q24 system (Qiagen) with PyroMark Q24 Advanced CpG Reagents (Qiagen, cat. No. 970922).

## Results

### RRBS analysis of different cellular fractions identified methylation differences in children at risk for type 1 diabetes

When including all the longitudinal samples into the differential methylation analysis model, we discovered 225, 114 and 87 DMCs in the CD4^+^ T cell, CD8^+^ T cell and CD4^−^CD8^−^ cell fractions, respectively, at FDR <0.1 and absolute coverage-corrected mean methylation difference >0.1 (Fig. 2, Table 1, ESM Table 3–5).

**Table 1:** Top-ranked DMCs at FDR <0.0001 identified between cases and controls in all longitudinal samples

DMC (hg19)	Fraction	Methylation difference	*p* value	FDR, <0.0001	Nearest gene	Genomic region
chr2:113192477	CD4^+^	0.130	8.45× 10^−13^	1.25× 10^−7^	*RGPD8*	Promoter
chr11:42897003	CD4^+^	0.456	1.42× 10^−12^	1.40× 10^−7^	*HNRNPKP3*	Intergenic
chr7:22861070	CD4^+^	−0.160	2.61× 10^−12^	1.93× 10^−7^	*TOMM7*	Intron
chr1:44878291	CD4^+^	−0.104	4.46× 10^−12^	2.64× 10^−7^	*RNF220*	Exon
chr11:118842572	CD4^+^	0.101	1.25× 10^−10^	3.71× 10^−6^	*FOXR1*	Promoter
chr14:103227394	CD4^+^	−0.150	5.86× 10^−10^	1.24× 10^−5^	*TRAF3*	Intergenic
chr6:121069653	CD4^+^	−0.124	6.88× 10^−10^	1.34× 10^−5^	*C6orf170*	Intergenic
chr9:137674085	CD4^+^	−0.113	7.25× 10^−10^	1.34× 10^−5^	*MIR3689C*	Intron
chr19:18118304	CD4^+^	−0.174	1.11× 10^−9^	1.94× 10^−5^	*ARRDC2*	Promoter
chr22:39633525	CD4^+^	0.189	1.77× 10^−9^	2.61× 10^−5^	*PDGFB*	Intron
chr16:32289963	CD8^+^	−0.130	5.21× 10^−11^	1.27× 10^−5^	*LOC390705*	Intergenic
chr2:27038339	CD8^+^	−0.186	2.78× 10^−10^	4.31× 10^−5^	*CENPA*	Intergenic
chr9:79557441	CD8^+^	0.154	5.37× 10^−10^	7.04× 10^−5^	*PRUNE2*	Intergenic
chr14:45343058	CD8^+^	−0.207	8.55× 10^−10^	9.11× 10^−5^	*C14orf28*	Intergenic
chr18:54333583	CD4^−^CD8^−^	0.131	1.26× 10^−17^	7.62× 10^−12^	*WDR7*	Intron
chr14:57523325	CD4^−^CD8^−^	−0.299	5.17× 10^−13^	1.25× 10^−7^	*EXOC5*	Intergenic
chr3:194706028	CD4^−^CD8^−^	−0.132	1.24× 10^−12^	2.51× 10^−7^	*XXYLT1*	Intergenic
chr5:173991159	CD4^−^CD8^−^	0.143	7.59× 10^−12^	1.32× 10^−6^	*MSX2*	Intergenic
chr19:38346420	CD4^−^CD8^−^	0.115	1.14× 10^−11^	1.72× 10^−6^	*LOC100631378*	Promoter
chr3:194705954	CD4^−^CD8^−^	−0.138	3.75× 10^−11^	5.06× 10^−6^	*XXYLT1*	Intergenic
chr6:166419166	CD4^−^CD8^−^	0.104	5.30× 10^−11^	6.44× 10^−6^	*LINC00473*	Intergenic
chr11:134709660	CD4^−^CD8^−^	0.215	2.73× 10^−10^	2.55× 10^−5^	*AK125040*	Intergenic

In the CD4^+^ T cells, the DMC located in the intergenic region between *TRAF3* and *RCOR1* was hypomethylated in cases. Furthermore, three highly significant DMCs were found at the promoters of protein-coding genes: *RGPD8*, *FOXR1* and *ARRDC2.* A variant in *ARRDC2* has been genetically associated with early-onset Crohn’s disease [27]. In CD8^+^ T cells, the most significant DMC was located in the intergenic region between the pseudogene *LOC390705* and a non-coding RNA, *LOC113002582.* In the CD4^−^CD8^−^ cell fraction, highly significant differences in methylation were observed near protein-coding genes *WDR7* and *EXOC5* (Table 1).

Further, to gain insights into biological functions of the genes near DMCs, we performed GO pathway enrichment analysis (Fisher’s exact test) on the sets of the nearest genes for each cell type. For CD8^+^ T cells, two GO terms were enriched: peptidyl-tyrosine phosphorylation (FDR = 0.035) and cell differentiation (FDR = 0.045). No GO terms were significantly enriched among the nearest genes for CD4^+^ T cells or CD4^−^CD8^−^ cell fractions.

### Most of the DMRs were cell type-specific

**Fig. 3:**
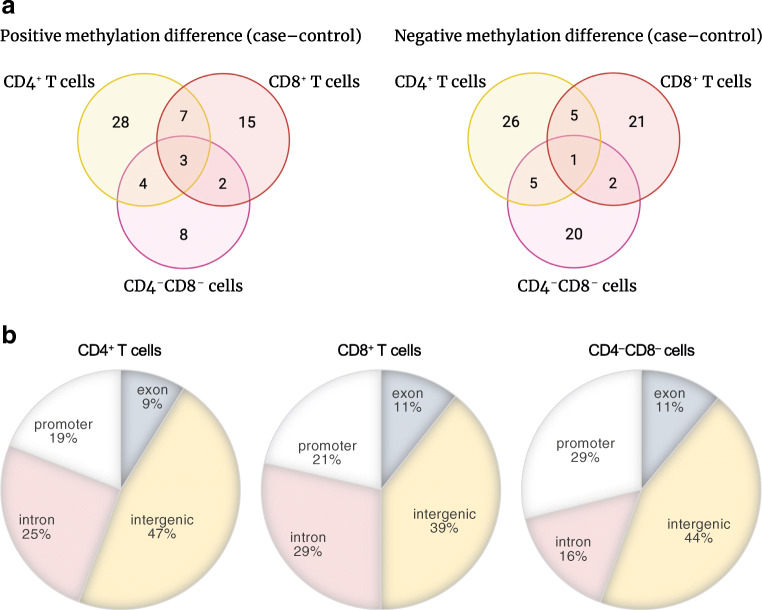
DMRs. (**a**) Venn diagrams of the numbers of DMRs detected in different cell fractions. A DMR is considered to be found in two cell fractions if at least one DMC within the DMR is found in both cell fractions. Positive methylation difference means the DMR was hypermethylated in cases, compared with controls; negative means hypomethylated in cases, compared with controls. The figures were created with BioRender. (**b**) Distribution of DMRs in genomic regions in CD4^+^ T cells, CD8^+^ T cells and CD4^−^CD8^−^ cell fractions

Although a single CpG site might affect gene expression, often adjacent CpG sites may act together in gene expression regulation [28]. Such DMCs can be combined into a differentially methylated region (DMR). Therefore, we combined individual DMCs within 2 kb with consistent methylation patterns into DMRs. We identified 79, 56 and 45 DMRs between the cases and controls in the CD4^+^ T cell, CD8^+^ T cell and CD4^−^CD8^−^ cell fractions, respectively (Fig. 3a, ESM Table 6–8). While the majority of DMRs were cell type-specific, three DMRs near the *FOXR1*, *RGPD8* and *LOC100128946* (also known as *LINC01310*) genes were hypermethylated and one DMR near the *LOC390705* pseudogene was hypomethylated in cases in all three cell fractions. These results suggest that differences in these three DMRs are conserved across cell types. It is also possible that DNA methylation at these DMRs regulates the expression of genes with a similar functional role in all three subsets.

### DMRs were distributed across different genomic regions

We characterised DMRs with respect to genomic regions (Fig. 3b). In all three fractions, the majority of DMRs were located in intergenic regions (47% in CD4^+^ T cells, 39% in CD8^+^ T cells and 44% in the CD4^−^CD8^−^ cell fraction). Introns accounted for 25% and 29% in CD4^+^ and CD8^+^ T cells, respectively, whereas approximately 20% of all DMRs were located in promoters in both fractions. However, in contrast to CD4^+^ and CD8^+^ T cells, the number of DMRs in promoter regions in CD4^−^CD8^−^ cell fractions constituted up to 30%. In total, 9–11% of DMRs were located in exons in all three cell fractions.

**Fig. 4:**
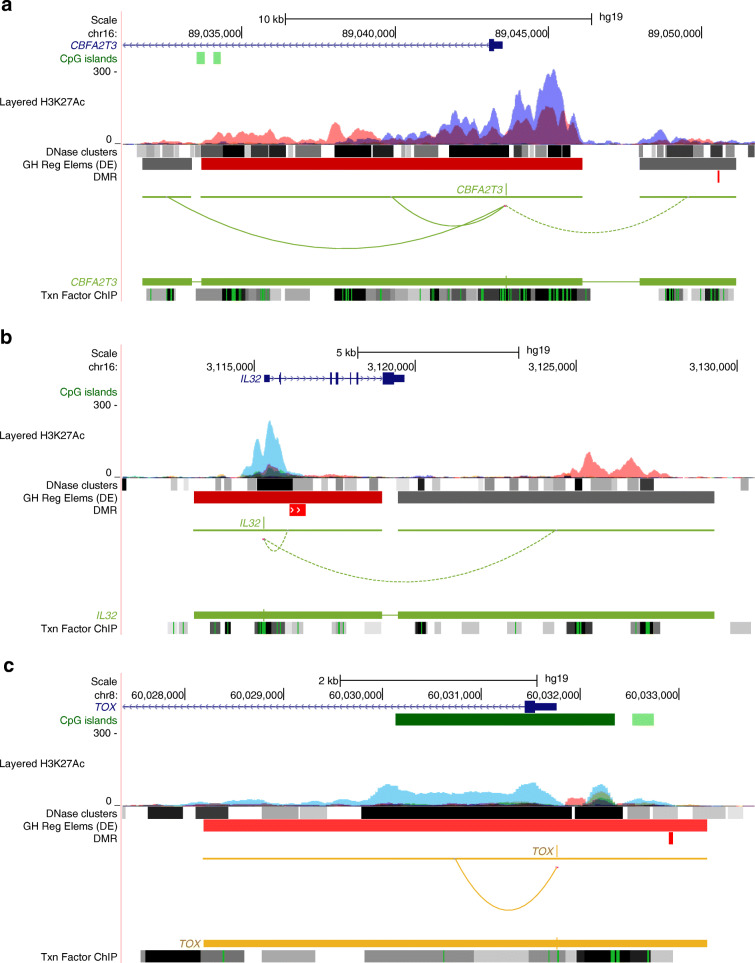
Annotations of the type 1 diabetes-associated DMRs. The genomic loci harbouring the DMRs at *CBFA2T3*, *IL32* and *TOX* were visualised with the UCSC Genome Browser. The DMRs are shown in red colour in the DMR track below the GeneHancer track in all of the panels. Six other reference tracks are displayed. (**a**) A DMR hypermethylated in cases vs controls (chr16:89050538–89,050,592) mapped in the intergenic region upstream of *CBFA2T3* in CD4^+^ T cells. It overlaps with an enhancer region. A long-distance chromatin interaction between this active regulatory element and *CBFA2T3* promoter is shown by a dashed line. (**b**) A DMR hypomethylated in cases vs controls at *IL32* gene locus (chr16:3116115–3,116,616) in CD8^+^ T cells is located at the promoter of the gene. (**c**) A DMR hypermethylated in cases vs controls in CD4^+^ T cells (chr8:60032900–60,032,942) is located approximately 1 kb upstream of *TOX* and overlaps with its promoter. GH Reg Elems (DE), GeneHancer regulatory elements and gene interactions (Double Elite); Txn Factor ChIP, Transcription factor chromatin immunoprecipitation sequencing clusters

The GeneHancer database was utilised to explore DMRs in the enhancer regions. Ten, four and seven DMRs were located in enhancer regions in CD4^+^ T cells, CD8^+^ T cells and the CD4^−^CD8^−^ cell fractions, respectively (ESM Tables 6–8). The DMR near *CBFA2T3* is a part of an enhancer that interacts with its promoter based on the GeneHancer database and may regulate its expression (Fig. 4a). *CBFA2T3* encodes a transcriptional repressor and plays a role in T cell development*.* A variant of this gene has been associated with autoimmune vitiligo [29]. We also found DMRs near extended promoter regions of several genes including *TOX* and *IL32* (Fig. 4b, c, ESM Tables 6–8) in the CD4^+^ and CD8^+^ T cell fractions, respectively. Interestingly, of the 47, 33 and 17 stand-alone DMCs, which were not part of DMRs, 12, ten and four were in the promoter or enhancer regions in CD4^+^ T cells, CD8^+^ T cells and the CD4^−^CD8^−^ cell fractions, respectively (ESM Table 3–5).

### High correlation between methylation and gene expression highlighted candidate genes of interest

**Fig. 5:**
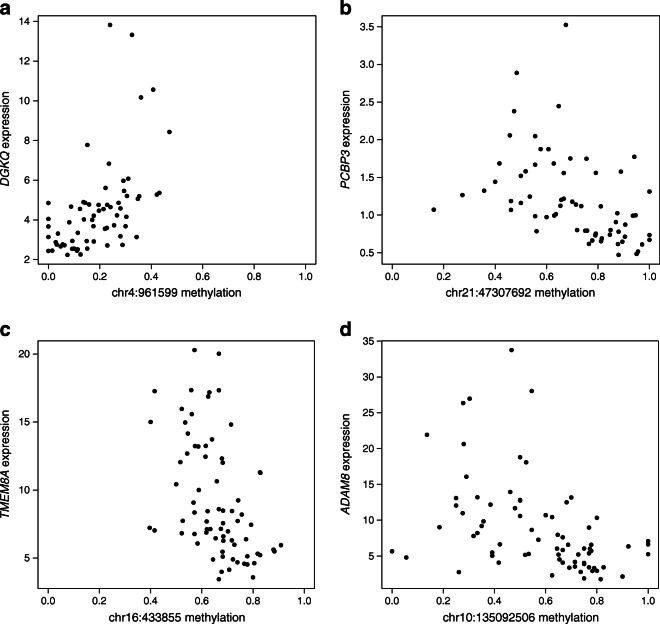
Methylation and gene expression correlation analysis. The most highly correlated expression–methylation pairs in CD4^+^ and CD8^+^ T cells. Spearman ρ (Spearman’s rank correlation coefficient) is between methylation proportion (*x*-axis) and reads per kb per million (RPKM)-normalised gene expression level (*y*-axis). (**a**, **b**) Expression–methylation pairs in the CD4^+^ T cells. The methylation of CpG site chr4:961599 at an intron of *DGKQ* gene positively correlated (Spearman *ρ* = 0.619) with gene expression. The methylation of CpG site chr21: 47307692 at an intron of *PCBP3* gene showed negative correlation (Spearman *ρ* = −0.591) with gene expression. (**c**, **d**) Expression–methylation pairs in the CD8^+^ T cells. The methylation of CpG site chr16:433855 near *TMEM8A* gene inversely correlated (Spearman *ρ* = −0.538) with gene expression. The methylation of CpG site chr10:135092506 at an exon of *ADAM8* gene showed negative correlation (Spearman *ρ* = −0.520) with gene expression. For a complete list of CpG sites and their correlation with gene expression, please see ESM Tables 3–5, 9–11

The availability of RNA-seq data from the purified cell fractions of the very same samples allowed us to assess a possible functional impact of the discovered DMCs on gene expression. We examined correlations between DNA methylation and gene expression, utilising our earlier published RNA-seq data [16] (ESM Tables 3–5, 9–11). We focused on DMCs that had a high Spearman rank correlation coefficient (>|0.5|) with gene expression (Table 2, Fig. 5). For example, in CD4^+^ T cells, we found two DMCs on the intron of *DGKQ* positively correlated with the expression of the gene (Table 2, Fig. 5a). Next, four DMCs on the intron of *PCBP3*, which is a probable enhancer region, showed an inverse correlation with its expression (Fig. 5b, Table 2). A DMC at the exon of *LOC642852* (also known as *LINC00205*) showed an inverse correlation with the expression of the *POFUT2* gene, which is approximately 30 kb away from the DMC. *POFUT2* encodes a fucosyltransferase (FUT), responsible for protein O-fucosylation, a post-translational modification process. Interestingly, a genetic risk variant for type 1 diabetes at *FUT2*, another FUT of a closely related family, was reported previously [30–32].

**Table 2:** DMCs across all longitudinal time points showing the highest Spearman rank correlation >|0.5| with gene expression levels

DMC (hg19)	Fraction	Methylation difference	*p* value	FDR	Nearest gene	Spearman correlation	Correlating gene
chr4:961599	CD4^+^	0.127	7.29 × 10^−5^	4.18 × 10^−2^	*DGKQ*	0.619	*DGKQ*
chr4:962384	CD4^+^	0.136	4.66 × 10^−5^	3.25 × 10^−2^	*DGKQ*	0.508	*DGKQ*
chr21:47307692	CD4^+^	−0.171	1.33 × 10^−5^	1.59 × 10^−2^	*PCBP3*	−0.591	*PCBP3*
chr21:47307758	CD4^+^	−0.210	2.25 × 10^−4^	7.82 × 10^−2^	*PCBP3*	−0.537	*PCBP3*
chr21:47308375	CD4^+^	−0.244	3.46 × 10^−4^	9.76 × 10^−2^	*PCBP3*	−0.537	*PCBP3*
chr21:47308422	CD4^+^	−0.208	3.80 × 10^−6^	7.98 × 10^−3^	*PCBP3*	−0.517	*PCBP3*
chr21:46714810	CD4^+^	−0.109	2.66 × 10^−6^	6.57 × 10^−3^	*LOC642852*	−0.561	*POFUT2*
chr7:76129434	CD4^+^	−0.165	1.29 × 10^−4^	5.64 × 10^−2^	*DTX2*	−0.539	*UPK3B*
chr16:433855	CD8^+^	−0.174	1.14 × 10^−9^	1.10 × 10^−4^	*LOC100134368*	−0.538	*TMEM8A*
chr10:135092506	CD8^+^	−0.118	5.12 × 10^−6^	3.96 × 10^−2^	*ADAM8*	−0.520	*ADAM8*
chr4:961576	CD4^−^CD8^−^	0.164	2.01 × 10^−6^	2.65 × 10^−2^	*DGKQ*	0.654	*DGKQ*
chr4:961573	CD4^−^CD8^−^	0.119	2.37 × 10^−5^	9.88 × 10^−2^	*DGKQ*	0.528	*DGKQ*

In CD8^+^ T cells, there were only two DMCs with a Spearman rank correlation >|0.5|. The DMC on chromosome 16 in the intron of *LOC100134368* had an inverse correlation with expression of *TMEM8A* (also known as *PGAP6)*, which is approximately 2 kb away from the DMC (Fig. 5c, Table 2). Another DMC in the exon of *ADAM8* correlated inversely with the expression of this gene (Fig. 5d, Table 2).

Further, we analysed CpG sites in our study for already published *cis-*eQTM associations, using data from a Dutch biobank of *cis-*eQTM effects based on 3841 whole blood samples [24] (ESM Tables 3–5, 9–11). The expression quantitative trait methylation (eQTM) effects were discovered with the 450 K DNA methylation array, which covers approximately 5% of CpG sites identified in our study. Among those available, we found three DMCs with known eQTM effects: chr4:81128398 near *PRDM8* (cg05474265) and chr8:144808965 at *FAM83H* (cg09380067) in CD4^+^ T cells, and chr2:73496203 at *FBXO41* (cg21918313) in the CD4^−^CD8^−^ cell fraction (ESM Tables 3–5, 9–11). The observed direction of expression–methylation correlation in our data was in line with the eQTM effect at CpG sites near *PRDM8* and *FBXO41*.

### Pre-seroconversion analysis revealed DNA methylation differences at a very early stage of type 1 diabetes

**Fig. 6:**
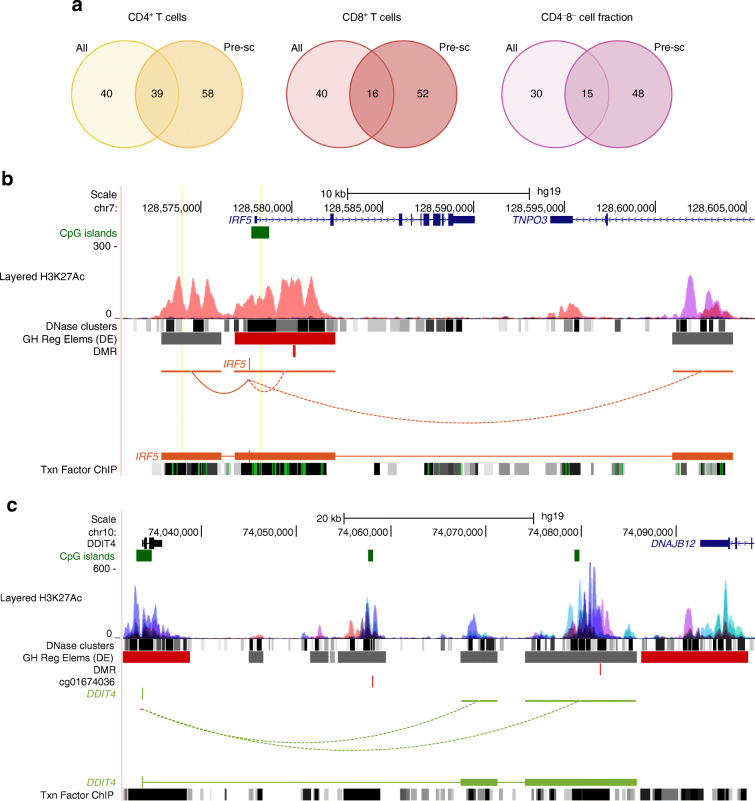
DNA methylation analysis prior to seroconversion. (**a**) Venn diagrams of the numbers of DMRs detected in different cell fractions for all longitudinal samples (indicated as All) and prior to seroconversion (indicated as Pre-sc). A DMR is considered to be found in two analyses if at least one DMC within the DMR is found in both cell fractions. Seroconversion was defined based on the measurements of autoantibodies IAA, GADA, IA-2A and ZnT8A in the participant’s serum (details in the Methods section). The figure was created with BioRender. (**b**) UCSC Genome Browser visualisation of a DMR hypermethylated in cases vs control participants at *IRF5* gene identified in CD4^+^ T cells prior to seroconversion. The genomic locus (chr7:128568936–128,609,758) includes the DMR (chr7:128580072–128,580,230). (**c**) The DMR (chr10:74082048–74,082,090) identified in this study and the DMC (chr10:74058002, cg01674036) found in the Paul et al study [12] located in the intergenic region between *DDIT4* and *DNAJB12* in CD4^+^ T cells prior to seroconversion; the DMC is shown below the DMR track, and the DMC and DMR are shown as vertical lines in respective tracks. Six other reference tracks are displayed. Long-distance chromatin interactions between the gene promoter and other active regulatory elements (indicated in grey colour in GeneHancer regulatory element track) are shown by curves connecting promoters and other regulatory elements. GH Reg Elems (DE), GeneHancer regulatory elements and gene interactions (Double Elite); Txn Factor ChIP, Transcription factor chromatin immunoprecipitation sequencing clusters

In addition to the differential methylation analysis of all longitudinal samples, we analysed DNA methylation in the samples taken before seroconversion to identify changes that occur prior to detection of islet autoantibodies. We refer to this analysis as pre-seroconversion analysis. Unless specified, the differential methylation analysis refers to the analysis where we take all the longitudinal time points into consideration. We identified 249, 144 and 143 DMCs between cases and controls in the CD4^+^ T cell, CD8^+^ T cell and CD4^−^CD8^−^ cell fractions, respectively (FDR <0.1 and coverage-corrected mean methylation difference >0.1) (ESM Tables 9–11). The numbers of DMRs between cases and controls discovered before seroconversion were 97, 68 and 63 in the CD4^+^ T cell, CD8^+^ T cell and CD4^−^CD8^−^ cell fractions, respectively (ESM Tables 12–14). Importantly, the overlay of DMRs identified in all longitudinal samples and DMRs discovered prior to the appearance of autoantibodies (Fig. 6a) revealed that the majority of DMRs were pre-seroconversion-specific. We found 58, 52 and 48 pre-seroconversion-specific DMRs in CD4^+^ T cell, CD8^+^ T cell and CD4^−^CD8^−^ cell fractions, respectively. These DMRs were not identified in the analysis across all longitudinal time points.

In CD4^+^ T cells, the most significant DMC was found at the promoter of *ARRDC2* gene and was hypomethylated in cases vs controls (Table 3). This DMC was also one of the most significant findings in the analysis of all longitudinal samples (Table 1). In CD8^+^ T cells, the most significant DMC was observed at the intron of the *PCBP3* gene. This CpG site is located in the plausible enhancer region (GeneHancer database) and may regulate the expression of *PCBP3*. Furthermore, methylation–expression analysis revealed a high correlation between methylation at *PCBP3* and expression of the gene (Table 4).

**Table 3:** Top-ranked DMCs at FDR <0.0001 identified between cases and controls prior to seroconversion

DMC (hg19)	Fraction	Methylation difference	*p* value	FDR, <0.0001	Nearest gene	Genomic region
chr19:18118304	CD4^+^	−0.185	4.22 × 10^−22^	1.64 × 10^−16^	*ARRDC2*	Promoter
chr7:3169658	CD4^+^	0.269	4.76 × 10^−13^	4.62 × 10^−8^	*BC038729*	Intergenic
chr10:99209697	CD4^+^	0.541	1.95 × 10^−12^	1.52 × 10^−7^	*ZDHHC16*	Intron
chr14:65542789	CD4^+^	−0.505	7.00 × 10^−12^	3.88 × 10^−7^	*LOC100506321*	Intron
chr8:530556	CD4^+^	−0.171	1.00 × 10^−11^	4.87 × 10^−7^	*TDRP*	Intergenic
chr21:47285711	CD8^+^	−0.124	9.16 × 10^−22^	4.78 × 10^−16^	*PCBP3*	Intron
chr17:71322457	CD8^+^	−0.113	1.92 × 10^−11^	2.28 × 10^−6^	*CDC42EP4*	Intergenic
chr19:12035022	CD8^+^	−0.150	3.10 × 10^−10^	2.31 × 10^−5^	*ZNF700*	Promoter
chr7:3169674	CD8^+^	−0.174	3.00 × 10^−10^	2.31× 10^−5^	*BC038729*	Intergenic
chr14:45343058	CD8^+^	0.189	6.22 × 10^−10^	4.33 × 10^−5^	*C14orf28*	Intergenic
chr11:134709660	CD4^−^CD8^−^	0.228	5.41 × 10^−17^	4.72 × 10^−11^	*AK125040*	Intergenic
chr2:113192534	CD4^−^CD8^−^	0.132	6.45 × 10^−13^	1.87 × 10^−7^	*RGPD8*	Promoter
chr8:144788521	CD4^−^CD8^−^	0.18	1.09 × 10^−12^	2.37 × 10^−7^	*CCDC166*	Intron
chr3:194706028	CD4^−^CD8^−^	−0.135	9.52 × 10^−12^	1.38 × 10^−6^	*XXYLT1*	Intergenic
chr6:163570666	CD4^−^CD8^−^	−0.219	5.53 × 10^−10^	4.25 × 10^−5^	*AK296276*	Intron

**Table 4:** DMCs in the analysis of only pre-seroconversion samples showing the highest Spearman rank correlation >|0.5| with gene expression levels

DMC (hg19)	Fraction	Methylation difference	*p* value	FDR	Nearest gene	Spearman correlation	Correlating gene
chr2:1817466	CD4^+^	0.269	1.88 × 10^−4^	8.42 × 10^−2^	*MYT1L*	−0.695	*PXDN*
chr2:1817753	CD4^+^	0.174	9.85 × 10^−5^	6.05 × 10^−2^	*MYT1L*	−0.629	*PXDN*
chr16:452426	CD4^+^	−0.124	7.79 × 10^−7^	3.36 × 10^−3^	*DECR2*	−0.623	*LA16c-OS12.2*
chr8:144659988	CD4^+^	0.104	1.43 × 10^−4^	7.35 × 10^−2^	*NAPRT1*	−0.600	*NAPRT1*
chr11:67351952	CD4^+^	−0.104	1.92 × 10^−5^	2.53 × 10^−2^	*GSTP1*	−0.567	*GSTP1*
chr5:179193600	CD4^+^	−0.197	4.95 × 10^−5^	4.17 × 10^−2^	*LTC4S*	−0.548	*RUFY1*
chr19:37807762	CD4^+^	0.138	1.49 × 10^−5^	2.17 × 10^−2^	*HKR1*	−0.540	*ZNF527*
chr7:76129273	CD4^+^	−0.174	3.28 × 10^−5^	3.34 × 10^−2^	*DTX2*	−0.536	*UPK3B*
chr2:131695535	CD4^+^	−0.214	6.79 × 10^−5^	5.00 × 10^−2^	*ARHGEF4*	0.600	*FAM168B*
chr4:961405	CD4^+^	0.325	1.29 × 10^−6^	4.84 × 10^−3^	*DGKQ*	0.504	*DGKQ*
chr8:144660722	CD8^+^	0.142	2.53 × 10^−6^	1.87 × 10^−2^	*NAPRT1*	−0.543	*NAPRT1*
chr21:47307825	CD8^+^	−0.142	5.84× 10^−5^	9.98× 10^−2^	*PCBP3*	−0.534	*PCBP3*
chr21:47285711	CD8^+^	−0.427	9.16 × 10^−22^	4.78 × 10^−16^	*PCBP3*	−0.512	*PCBP3*
chr21:47307753	CD8^+^	−0.129	6.48 × 10^−9^	3.38 × 10^−4^	*PCBP3*	−0.501	*PCBP3*
chr17:71322457	CD8^+^	0.548	1.92 × 10^−11^	2.28 × 10^−6^	*CDC42EP4*	0.516	*SLC39A11*
chr11:65359968	CD4^−^CD8^−^	−0.320	6.92 × 10^−9^	3.14 × 10^−4^	*KCNK7*	−0.545	*LTBP3*
chr16:1480869	CD4^−^CD8^−^	0.135	7.38 × 10^−6^	3.04 × 10^−2^	*C16orf91*	0.611	*TSR3*
chr1:3811367	CD4^−^CD8^−^	0.238	9.27 × 10^−10^	5.77 × 10^−5^	*C1orf174*	0.558	*DFFB*
chr19:50962487	CD4^−^CD8^−^	0.121	5.31 × 10^−5^	9.00 × 10^−2^	*EMC10*	0.527	*KCNC3*
chr19:1047251	CD4^−^CD8^−^	0.171	4.93 × 10^−5^	8.68 × 10^−2^	*ABCA7*	0.501	*ATP5D*

Notably, in CD4^+^ T cells we identified hypermethylation in case compared with control participants at the promoter of the transcription factor *IRF5* and *DNAJB12* (Fig. 6b, c, ESM Table 12)*.*

### Several DMCs were validated using pyrosequencing analysis in a subset of samples

The RRBS results were validated by bisulphite pyrosequencing of the CD4^+^ and CD8^+^ T cells of three case–control pairs from the same cohort. We selected nine DMCs in CD4^+^ T cells and one DMC in CD8^+^ T cells based on statistical significance and the consistency of pairwise methylation difference profiles.

The direction of methylation difference was concordant between RRBS and bisulphite pyrosequencing results for eight out of ten selected targets (Table 5). High positive correlations between RRBS and pyrosequencing results were observed for CpG sites near *ARRDC2* (chr19:18118304), *PCBP3* (chr21:47307815) and *TRAF3* (chr14:81128398) and also at the intergenic CpG site near *PRDM8* (chr4:81128398) with a known eQTM effect on the gene expression. In the CD8^+^ T cells, we validated the DMC near *IL32* (chr16:3116115), which was hypomethylated on the promoter region in type 1 diabetes, compared with control participants.

**Table 5:** Validation of the RRBS results by bisulphite pyrosequencing

Selected targets for validation						Validation results			
DMC (hg19)	Nearest gene	Methylation difference	*p* value	FDR	Fraction	Concordant direction of difference	Correlation	*p* value	FDR
chr19:18118304	*ARRDC2*	−0.174	1.11 × 10^−9^	1.94 × 10^−5^	CD4^+^	Yes	0.85	0.03	0.15
chr21:47307815	*PCBP3*	−0.207	7.69 × 10^−8^	6.48 × 10^−4^	CD4^+^	Yes	0.86	0.03	0.15
chr14:103227394	*TRAF3*	−0.15	5.86 × 10^−10^	1.24 × 10^−5^	CD4^+^	Yes	0.82	0.05	0.15
chr4:81128398	*PRDM8*	−0.143	4.71 × 10^−6^	8.92 × 10^−3^	CD4^+^	Yes	0.71	0.11	0.23
chr1:228659024	*Histone3*	0.147	3.33 × 10^−8^	3.40 × 10^−4^	CD4^+^	Yes	0.74	0.09	0.23
chr16:3116115	*IL32*	−0.141	1.68 × 10^−7^	3.82 × 10^−3^	CD8^+^	Yes	0.14	0.79	0.79
chr22:39633525	*PDGFB*	0.189	1.77 × 10^−9^	2.61 × 10^−5^	CD4^+^	Yes	0.62	0.19	0.32
chr8:60032942	*TOX*	0.113	3.20 × 10^−5^	2.63 × 10^−2^	CD4^+^	Yes	0.25	0.63	0.70
chr18:77398359	*CTDP1*	0.144	2.46 × 10^−7^	1.26 × 10^−3^	CD4^+^	No	0.46	0.36	0.49
chr2:113192477	*RGPD8*	0.13	8.45 × 10^−13^	1.25 × 10^−7^	CD4^+^	No	0.43	0.39	0.49

The first six columns contain information about selected targets. In column 7 we indicate whether the methylation difference between the cases and controls was concordant (to the same direction) in the pyrosequencing results compared with RRBS results. Column 8 contains the Pearson correlation coefficient between pyrosequencing and RRBS results (methylation proportions of the six samples that were pyrosequenced). Columns 9 and 10 contain raw and adjusted *p* values (FDR), respectively

## Discussion

In this study, we identified DNA methylation changes in children developing type 1 diabetes, compared with their matched control participants, before diabetes diagnosis and even before the appearance of autoantibodies associated with type 1 diabetes. Analysing fractionated PBMC samples allowed us to discover immune cell subset-specific DNA methylation changes. Importantly, DNA methylation changes were observed in genes associated with type 1 diabetes, including *IL32*, *TRAF3* and *DGKQ*, and in novel candidate genes, which have not been linked to the disease, such as *ARRDC2* and *PCBP3*. Using pyrosequencing we validated the direction of methylation difference identified by RRBS for eight out of ten selected targets. Since the number of case individuals in this prospective study cohort was limited (*n* = 7), we selected matched control individuals to control for effects of confounding factors, such as sex and place of birth. The risk of error was further minimised by analysing several time points for each individual. Even with these measures taken, the number of individuals is an important limitation of this study, and the results need to be validated in a larger cohort.

A strength of our study is the correlation of DNA methylation with gene expression from the purified cell type of the very same samples from our earlier report [16], which enabled us to highlight candidate genes whose expression might be influenced by differential methylation. For instance, an intron of *PCBP3* had a locus hypomethylated in cases in CD4^+^ T cells across all time points and in CD8^+^ T cells before seroconversion, and it had an inverse correlation with the gene expression. RNA binding proteins of the poly(rC) binding protein (PCBP) family are important in regulating gene expression at the post-transcriptional level. Further, another locus at the promoter of *IL32* was also hypomethylated in cases compared with controls in the analysis of all longitudinal samples in CD8^+^ T cells. Methylation–expression analysis showed a weak inverse correlation between *IL32* mRNA expression and promoter methylation, suggesting that epigenetic changes at the promoter of *IL32* lead to a higher expression of this cytokine. Interestingly, IL-32 was significantly upregulated at the mRNA level in cases compared with controls in this cohort. IL-32, a proinflammatory cytokine, was shown to be upregulated at either mRNA or protein level in several autoimmune disorders [33]; however, the precise role of this cytokine remains to be elucidated.

Next, we observed hypermethylation in cases vs controls at a locus near *DGKQ*, which correlated positively with the expression of this gene in CD4^+^ T cells and CD4^−^CD8^−^ cell fractions. This finding suggests that the differential methylation at *DGKQ* may result in its higher expression in type 1 diabetes cases. This observation is in line with the results of the transcriptomic analysis of CD4^+^ T cells from the same cohort [16]. Diacylglycerol kinase theta (DGKQ) is a member of a family of diacylglycerol (DAG) kinases, which have critical roles in T cell receptor (TCR) signalling [34]. The kinase inversely regulate T cell activation, by terminating DAG-mediating signalling [35]. Interestingly, a recent integrative analysis of genetic association, functional genomics data and protein–protein networks identified *DGKQ* as a potential novel type 1 diabetes therapeutic gene target along with other novel and known targets, e.g., *IL2RA*, *IL6ST*, *IL6R* and *TYK2* [3].

The type 1 interferon transcriptional signature is associated with the development of type 1 diabetes [36, 37]. In CD4^+^ T cells of pre-seroconversion samples, we observed hypermethylation at the promoter of *IRF5* (Fig. 6b, ESM Table 12). This DMR is located in the vicinity (less than 7 kb) of multiple SNPs that were previously associated with RA [38, 39], systemic lupus erythematosus (SLE) [40, 41] and IBD [42]. This finding suggests that the transcription factor may have a role at the very early stages of islet autoimmunity. Further, in CD8^+^ T cells, we found hypermethylation at an exon of *IRF1* gene (ESM Table 7). Interferon regulatory factor 1 (IRF1) is important in the host immune response to pathogens and CD8^+^ T cell maturation [43]. Further, we found hypomethylated DMRs at *TRAF3* in CD4^+^ T cells and CD4^−^CD8^−^ cell fractions. *TRAF3* is also critical in regulation of type 1 interferon production and plays a role in antiviral immune response [44]. In CD4^+^ T cells, hypomethylation at *TRAF3* had moderate positive correlation with the gene expression (Spearman *ρ* = 0.334), which might result in lower gene expression in cases vs controls. TNF receptor-associated factor 3 (TRAF3) was shown to be recruited in the TCR/CD28 signalling complex in murine CD4^+^ T cells and critical for T cell-mediated immunity to infection [45].

Thymocyte selection-associated high mobility group box protein (TOX) is a key transcriptional regulator indispensable for CD4^+^ T cell development in the thymus [46]. In addition, it drives epigenetic remodelling of CD8^+^ T cells towards an exhausted phenotype in chronic infections [47, 48]. Notably, in the CD4^+^ T cells, we identified a hypermethylation in cases at the intergenic region near the TSS of the *TOX* gene. The methylation at this region had a weak negative correlation (−0.157) with gene expression, suggesting that hypermethylation of this locus in cases may favour T cell differentiation towards an effector phenotype in type 1 diabetes patients. It was reported that autoreactive CD8^+^ T cells with an exhaustion-like profile associated with slow type 1 diabetes progression [49] and preservation of beta cell function in type 1 diabetes patients [50]. Further, genetic variants at *TOX* are among novel risk loci for type 1 diabetes [51].

Our recent study aimed at identifying umbilical cord blood DNA methylation associated with type 1 diabetes progression [19], as well as a previous study by another group [12], did not find any difference in DNA methylation, suggesting that significant DNA methylation differences in children progressing to the disease are not yet present at birth or that they are too subtle to be identified with the current methods and cohort size.

A recent study by Johnson et al examined DNA methylation changes in blood samples longitudinally collected before diagnosis of type 1 diabetes from children enrolled in the prospective DAISY cohort using Illumina 450 K and EPIC platforms [14]. They observed 28 DMRs associated with later development of the disease. We found no overlap with the CpG sites identified in the study by Johnson et al. This may perhaps be due to different methodologies and to the fact that Johnson et al analysed peripheral whole blood samples, whereas we examined DNA methylation in purified cell fractions of PBMCs. Despite these differences, both studies identified pre-seroconversion changes in DNA methylation, suggesting that these may predict disease progression before the appearance of autoantibodies. However, more studies are needed to validate the findings in independent, larger cohorts, with replication in other populations.

Interestingly, Paul et al [12] identified one DMC (hg19: chr10:74058002) in purified CD4^+^ T cells between individuals with type 1 diabetes and their healthy co-twins in the intergenic region between *DDIT4* and *DNAJB12*. In our study, we found a DMR (chr10:74082048-–74,082,090) region hypermethylated in cases in CD4^+^ T cells in pre-seroconverted samples located in the same intergenic region (Fig. 6c).

In conclusion, our results provide novel insights into cell type-specific DNA methylation changes associated with type 1 diabetes development. These findings provide the basis for further studies to find an early methylation signature, which may be useful for disease prediction and management.

## Supplementary Information

ESM (PDF 2229 kb)

## Data Availability

The analysed datasets are available through ArrayExpress accession E-MTAB-11088. Due to privacy reasons, the raw data are available through the corresponding author upon reasonable request.

## Abbreviations

*cis*-eQTM: *cis*-expression quantitative trait methylation
DAG: Diacylglycerol
DAISY: The Diabetes Autoimmunity Study in the Young
DMC: Differentially methylated CpG site
DMR: Differentially methylated region
eQTM: Expression quantitative trait methylation
FDR: False discovery rate-corrected *p* value
FUT: Fucosyltransferase
GADA: Autoantibodies targeting glutamic acid decarboxylase
GO: Gene Ontology
IA-2A: Autoantibodies targeting islet antigen-2
IAA: Autoantibodies targeting insulin
IBD: Inflammatory bowel disease
PBMC: Peripheral blood mononuclear cell
RA: Rheumatoid arthritis
RNA-seq: RNA sequencing
RRBS: Reduced representation bisulphite sequencing
RU: Relative units
TCR: T cell receptor
TSS: Transcription start site
ZnT8A: Autoantibodies targeting zinc transporter-8
