# Supplementary material for: Early DNA methylation changes in children developing beta cell autoimmunity at a young age
Source: Diabetologia. 2022 Feb 10;65(5):844–60. doi: 10.1007/s00125-022-05657-x (PMC8960578; doi:10.1007/s00125-022-05657-x)
Supplement: Supplementary file 1 — (PDF 2229 kb) [file 125_2022_5657_MOESM1_ESM.pdf]

ELECTRONIC SUPPLEMENTARY MATERIAL (ESM)

ESM METHODS

RRBS data analysis

First, the sequenced reads (fastq files) were trimmed using TrimGalore (https://www.bioinformatics.babraham.ac.uk/projects/trim_galore/) version 0.4.1 in paired end RRBS mode. Quality control was done by examining the fastQC version 0.11.4. Reads were aligned using Genome Reference Consortium Human Build 37 (GRCh37/hg19) (and the lambda phage genome simultaneously with Bowtie2 within Bismark version 0.21.0 [1] with Bismark's default parameters in paired end mode. End repair biases were trimmed at the methylation extraction call step. The detailed methodologies of methylation call extraction, conversion efficiency calculation, removal of M-biases, SNP removal, and other steps were as described [2]. To remove most PCR duplication biases, extremely high coverages (>99th percentile) were replaced by NA. Then, pairs of technical replicates were merged by summing their methylated and total numbers of reads. Further, a minimum coverage 10 was required in at least two matching time points in at least five (out of seven) case-control pairs. The coverage filtering was carried out separately for $CD4^+$, $CD8^+$ T cells, and $CD4^-CD8^-$ cell fractions. The numbers of CpG sites in the filtered matrices were 1.95, 2.46, and 1.79 million in the $CD4^+$, $CD8^+$ T cells, and $CD4^-CD8^-$ cell fractions, respectively. For principal component analysis, missing values at each CpG site were imputed by the median over samples with non-missing values. This was done separately for the $CD4^+$, $CD8^+$ T cells, and $CD4^-CD8^-$ cell fractions. Principal component (PC) analysis was performed as described earlier [2], and PC1 and PC2 were included in the differential methylation analysis to account for technical variation in the data. The detection of differentially methylated CpG sites (DMCs) was performed using a readily implemented generalized linear mixed effects model PQLseq [3] version 1.1. within R version 3.6.1 [4]. Class (case/control), age, principal components 1 and 2 and the pair of individuals were included as fixed effect covariates, and each individual was modeled as a random effect. The pairs of individuals had been matched according to sex, age, and place of birth and allocated to the same library preparation batches and sequencing lanes. In $CD8^+$ T cells and $CD4^-CD8^-$ cell fractions, most samples from each pair of individuals were in the same batch and set of lanes, whereas in $CD4^+$ T

cell data the different time points were mostly on different lanes. However, each pair of samples (such as the 3-month sample of case 1 and 3- month sample of control 1) was always in the same batch and set of lanes. Therefore, the sample pair was included as a covariate for $CD4^{+}$ T cell data, instead of age and pair of individuals. The modeling of $CD4^{+}$ T cell data was performed in a fully paired way. That is, at each CpG site both samples within a sample pair were excluded (and the sample pair removed from the design matrix) if coverage was 0 in one of them. The penalized quasi likelihood optimization procedure of PQLseq converged for 71.2% and 69.8% of CpG sites in $CD8^{+}$ T cell and $CD4^{-}CD8^{-}$ cell fraction data, respectively, when full data was used, and 44.1% and 50.3% when only pre-seroconversion samples were used. In $CD4^{+}$ T cell data the corresponding numbers were 15.7% and 20.5%, owing to the much larger number of binary covariates (sample pairs instead of pairs of individuals). The CpG sites for which the model did not converge were excluded from further analysis. The Wald test *p* values computed within PQLseq were false discovery rate (FDR) corrected [5] and CpG sites with FDR < 0.1 and absolute coverage-corrected mean methylation difference > 0.1 were considered as DMCs. Coverage-corrected mean methylation difference is calculated as sum(number of methylated reads in cases)/sum(number of total reads in cases) – sum(number of methylated reads in controls)/sum(number of total reads in controls). To detect differentially methylated regions (DMR), we utilized the adjust-function implemented in package RADMeth [6] within Methpipe version 3.4.3. on the uncorrected Wald test *p* values after sorting the CpG sites by chromosome and location. This function uses a weighted Z-test to combine spatially correlated *p* values and then performs a Benjamini-Hochberg correction. These spatially adjusted FDR-corrected *p* values were not used to determine the significance but only as one criterion to detect CpG sites that belong to the same DMR around a DMC. A DMR was defined as a genomic region of maximum length 2 kb, including two or more CpG sites, at least one of which had to be a DMC (FDR < 0.1 before any spatial adjustment), with spatially adjusted FDR-corrected P value < 0.05 and absolute coverage-corrected mean methylation difference > 0.1, at least 90% of which had to have the same direction of methylation difference.

References

1. Krueger F, Andrews SR (2011) Bismark: a flexible aligner and methylation caller for Bisulfite-Seq applications. Bioinformatics 27(11):1571–1572. https://doi.org/10.1093/bioinformatics/btr167
2. Laajala E, Halla-aho V, Grönroos T, et al (2021) Permutation-based significance analysis reduces the type 1 error rate in bisulfite sequencing data analysis of human umbilical cord blood samples. bioRxiv 2021.05.18.444359. (Preprint). 3 July 2021. Available from: https://doi.org/10.1101/2021.05.18.444359 (accessed 9 Dec 2021)
3. Sun S, Zhu J, Mozaffari S, Ober C, Chen M, Zhou X (2019) Heritability estimation and differential analysis of count data with generalized linear mixed models in genomic sequencing studies. Bioinformatics 35(3):487–496. https://doi.org/10.1093/bioinformatics/bty644
4. R Core Team (2020) R: A language and environment for statistical computing. R Foundation for Statistical Computing, Vienna, Austria.
5. Benjamini Y, Hochberg Y (1995) Controlling the False Discovery Rate: A Practical and Powerful Approach to Multiple Testing. Journal of the Royal Statistical Society Series B (Methodological) 57(1):289–300
6. Dolzhenko E, Smith AD (2014) Using beta-binomial regression for high-precision differential methylation analysis in multifactor whole-genome bisulfite sequencing experiments. BMC Bioinformatics 15(1):215. https://doi.org/10.1186/1471-2105-15-215

ESM Table 1. The information on the avalaible samples for each paticipant, cell fraction and timepoint

Fraction	CD4+	CD8+	CD4-CD8-	CD4+	CD8+	CD4-CD8-	CD4+	CD8+	CD4-CD8-	CD4+	CD8+	CD4-CD8-	CD4+	CD8+	CD4-CD8-	CD4+	CD8+	CD4-CD8-
Timepoint	3	3	3	6	6	6	12	12	12	18	18	18	24	24	24	36	36	36
Case 1																		
Case 2										x								
Case 3							x			x		x	x	x	x			
Case 5												x						
Case 9																		
Case 10	x	x	x										x	x	x			
Case 11				x	x	x												
Control 1-1																		
Control 2-1										x								
Control 3-2							x			x		x	x	x	x			
Control 5-2																		
Control 9-2																		
Control 10-1	x	x	x				x	x	x				x	x	x			
Control 10-2	x	x	x								x		x	x	x		x	
Control 11-2				x	x	x												

Gray color indicates samples which were analysed. X indicates missing samples.

ESM Table 2. Description of the study participants.

Case/Control	Study site	Sex	T1D Diagnosis	Diagnosis age	HLA risk category	Family T1D diagnosis history	Sampling month	IAA (> 2.80 RU)	GADA (> 5.36 RU)	IA-2A (> 0.77 RU)	ZnT8A (> 0.61 RU)	ICA (> 2.5 JDFU)
Case 1	FINLAND	Female	Yes	3,2	Moderately increased	Father age 9	0	0,32	0,18	0,06	0,07	3
							3	0,01	0	0,08	0,06	0
							6	0,45	0	0,16	0,08	0
							12	**14,08**	**21,75**	0,08	0,3	0
							18	**53,07**	**1933,3**	**88,23**	**6,37**	374
							24	**14,44**	**305,98**	**510,62**	**12,89**	747
							36	2,3	**119,94**	**678,3**	**22,46**	512
Case 2	FINLAND	Male	No		Moderately increased	Father age 27	0	0	0	0,13	0,05	0
							3	0	0	0,14	0,06	0
							6	0	0	0,15	0,06	0
							12	**13,46**	0	0,15	0,02	0
							18	**12,75**	3,46	0,07	0,1	6
							24	**7,28**	**22,16**	0,11	0,15	0
							36	**5,96**	**173,27**	0,15	**7,53**	512
Case 3	FINLAND	Male	Yes	3,67	Moderately increased	Maternal uncle age 12	0	0	0	0,07	0,12	0
							3	0	0	0,09	0,04	0
							6	0,42	0	0,08	0,03	0
							12	0,7	0,07	0,1	0,05	0
							18	**8,93**	0,71	0,08	0,14	6
							24	**30,08**	**195,49**	**1013,95**	**13,67**	1024
							36	**11,9**	**64,38**	**894,54**	**36,06**	2048
Case 5	FINLAND	Female	Yes	2,63	High	Maternal grandmother age 15	0	0,06	0	0,04	0,05	0
							3	0	0	0,05	0,06	0
							6	0,22	0	0,07	0,06	0
							12	0,09	1,1	0,1	0,03	0
							18	2,42	0	0,17	0,07	0
							24	**6,49**	2,64	**19,82**	**3,58**	47
							36	**3,97**	0,32	**74,61**	0,5	256
Case 9	FINLAND	Male	No, transient abs		Moderately increased		0	0	0	0,14	0,06	0
							3	0	0	0,08	0,13	0
							6	1,36	0	0,07	0,07	0
							12	0,39	1,43	0,08	0,06	0
							18	**12,28**	**11,57**	0,11	0,1	4
							24	1,55	0,99	0,16	0,1	0
							36	**4,7**	0	0,09	0,13	0
Case 10	ESTONIA	Female	No, transient abs		Slightly increased		0	0	0	0,13	0,15	0
							6	0,04	0	0,05	0,05	0
							12	**21,78**	**18,46**	0,07	0,09	0
							18	**34,01**	1,94	0,08	0,12	0
							36	**8,09**	0	0,14	0,1	0
Case 11	ESTONIA	Female	Yes	2,41	Moderately increased	Father age 5	0	0,53	0	0,09	0,04	0
							3	0,05	0	0,08	0,03	0
							12	0,98	3,95	0,09	0,11	0
							18	0,54	**131,68**	0,19	0,17	0
							24	**8,59**	**189,7**	0,39	0,13	512
							36	**32,98**	**26,36**	**3,48**	0,15	16
Control 1	FINLAND	Female	No		Moderately increased		0	0,19	0,26	0,08	0,06	-
							3	0	0	0,06	0,04	-
							6	0	0	0,08	0,06	-
							12	0,28	0,07	0,09	0,07	-

						18	0	0	0,07	0,07	-
						24	0,28	1,56	0,09	0,13	-
						36	0,92	0,71	0,11	0,09	-
Control 2	FINLAND	Male	No	Moderately increased		0	0,31	0	0,02	0,18	-
						3	0,14	0	0,02	0,1	-
						6	0,63	0	0	0,07	-
						12	0,2	0	0,06	0,06	-
						18	0	0	0,16	0,1	-
						24	0	0,93	0,1	0,05	-
						36	0,14	0	0,1	0,09	-
Control 3	FINLAND	Male	No	Moderately increased		0	0,62	0	0,09	0,02	-
						3	0	0	0,13	0,03	-
						6	0,53	0	0,11	0,03	-
						12	0	0	0,12	0,05	-
						18	0	0	0,09	0,07	-
						36	0,23	0	0,14	0,11	-
Control 5	FINLAND	Female	No	High	Father age 6	3	0,36	0	0,1	0,09	-
						6	0,34	0	0,07	0,07	-
						12	0,02	1,62	0,08	0,07	-
						18	0,46	1,57	0,07	0,15	-
						24	0,86	0,39	0,07	0,11	-
						36	0	0	0,07	0,07	-
Control 9	FINLAND	Male	No	Moderately increased		0	0	0	0,12	0,07	-
						3	0,77	0	0,32	0,1	-
						6	1,03	0	0,09	0,08	-
						12	1,41	0,21	0,06	0,1	-
						18	0,28	0,72	0,11	0,09	-
						24	0,38	0	0,11	0,09	-
						36	0	0	0,07	0,1	-
Control 10.1	ESTONIA	Female	No	Slightly increased		0	0	0,24	0,08	0,05	-
						18	0,49	1,14	0,1	0,14	-
						36	0,68	0	0,12	0,1	-
Control 10.2	ESTONIA	Female	No	Slightly increased		0	0	0	0,15	0,08	-
						6	0,17	0,09	0,08	0,09	-
						12	0,56	0,21	0,13	0,07	-
						18	0,39	0	0,08	0,12	-
						36	0,62	0	0,09	0,09	-
Control 11	ESTONIA	Female	No	Moderately increased		0	0,7	0	0,12	0,03	-
						3	0,35	0	0,06	0,05	-
						12	0,95	0,05	0,11	0,1	-
						18	0,12	0	0,13	0,08	-
						24	0,75	0	0,12	0,1	-
						36	0,53	0	0,13	0,11	-

The columns 9-12 contain information about autoantibodies values against insulin (IAA), glutamic acid decarboxylase (GADA), islet antigen-2 (IA-2A), zinc transporter 8 (ZnT8). A sample was considered seropositive (in bold) when any of the autoantobodies exceeded the tresholds (indicated in the column header). The column 13 contains information about islet cell antibodies measured in autoantibody-positive subjects. The detection limit in the assay was 2.5 JDFU. T1D, type 1 diabetes; RU, relative units; ICA, islet cell antibodies; JDFU, juvenile diabtes foundation unit.

ESM Table 3. Differentially methylated CpGs identified in the CD4$^+$ T cell fraction between cases and controls in all longitudinal samples

Methylation difference				Nearest gene			Methylation–expression correlation analysis			eQTM analysis				GeneHancer database	
CpG site	P value	FDR	Methylation difference	Nearest gene	Distance to nearest gene	Genomic part	Nearest gene correlation, Spearman rho	The highest observed correlation, Spearman rho	Correlating gene	CpG name	eQTM, FDR	Overall Z Score	CpG found on 450K	GeneHancer	Genes possibly regulated by standalone DMCs
chr2:113192477	8,45E-13	1,25E-07	0.13	*RGPD8*	-416	promoter	-0.293	-0.293	*RGPD8*	NA	NA	NA	FALSE		
chr11:42897003	1,42E-12	1,40E-07	0.456	*HNRNPKP3*	393917	intergenic	NA	NA	*NA*	NA	NA	NA	FALSE		
chr7:22861070	2,61E-12	1,93E-07	-0.16	*TOMM7*	1402	intron	0.183	-0.28	*FAM126A*	NA	NA	NA	FALSE		
chr1:44878291	4,46E-12	2,64E-07	-0.104	*RNF220*	7243	exon	-0.061	-0.277	*DMAP1*	NA	NA	NA	FALSE		
chr11:118842572	1,25E-10	3,71E-06	0.101	*FOXR1*	157	promoter	NA	0.291	*RPS25*	NA	NA	NA	FALSE		
chr14:103227394	5,86E-10	1,24E-05	-0.15	*TRAF3*	-16422	intergenic	0.307	0.307	*TRAF3*	NA	NA	NA	FALSE		
chr6:121069653	6,88E-10	1,34E-05	-0.124	*C6orf170*	488556	intergenic	NA	NA	*NA*	NA	NA	NA	FALSE		
chr9:137674085	7,25E-10	1,34E-05	-0.113	*MIR3689C*	67131	intron	NA	0.221	*FCN1*	NA	NA	NA	FALSE		
chr19:18118304	1,11E-09	1,94E-05	-0.174	*ARRDC2*	-673	promoter	-0.087	0.297	*JAK3*	NA	NA	NA	FALSE		
chr22:39633525	1,77E-09	2,61E-05	0.189	*PDGFB*	3390	intron	NA	-0.167	*APOBEC3D*	NA	NA	NA	FALSE		
chr7:157225446	3,31E-09	4,08E-05	-0.11	*DNAJB6*	21533	intergenic	0.122	0.18	*RP4-814D15.1*	NA	NA	NA	FALSE		
chr20:25847039	6,97E-09	7,11E-05	0.122	*FAM182B*	1748	intron	NA	0.007	*ZNF337*	NA	NA	NA	FALSE		
chr9:137673996	7,32E-09	7,22E-05	-0.173	*MIR3689C*	67220	intron	NA	0.276	*FCN1*	NA	NA	NA	FALSE		
chr20:47013333	8,16E-09	7,79E-05	-0.181	*LINC00494*	18945	intergenic	NA	NA	*NA*	NA	NA	NA	FALSE		
chr11:42894941	1,46E-08	1,23E-04	0.426	*HNRNPKP3*	395979	intergenic	NA	NA	*NA*	NA	NA	NA	FALSE		
chr19:1336301	1,94E-08	1,59E-04	0.143	*MUM1*	-18675	intergenic	-0.016	0.301	*REEP6*	NA	NA	NA	FALSE		
chr17:72916077	3,45E-08	2,55E-04	0.23	*USH1G*	3275	exon	NA	0.414	*KCTD2*	NA	NA	NA	FALSE		
chr6:142623625	6,26E-08	4,52E-04	-0.125	*GPR126*	571	promoter	NA	-0.024	*VTA1*	NA	NA	NA	FALSE		
chr19:46909138	7,53E-08	5,18E-04	0.121	*CCDC8*	7782	intergenic	NA	0.236	*CALM3*	NA	NA	NA	FALSE		
chr7:48686394	1,02E-07	6,57E-04	0.129	*ABCA13*	191762	exon	NA	NA	*NA*	NA	NA	NA	FALSE		
chr18:77398359	2,46E-07	1,26E-03	0.144	*CTDP1*	-41442	intergenic	-0.036	-0.084	*NFATC1*	NA	NA	NA	FALSE		
chr2:27038408	2,75E-07	1,34E-03	-0.157	*CENPA*	29528	intergenic	NA	-0.248	*TMEM214*	NA	NA	NA	FALSE		
chr9:46355235	2,74E-07	1,34E-03	-0.148	*Y_RNA*	-7370	intergenic	NA	NA	*NA*	NA	NA	NA	FALSE		
chr20:47013383	4,17E-07	1,89E-03	-0.166	*LINC00494*	18995	intergenic	NA	NA	*NA*	NA	NA	NA	FALSE		
chr21:45565981	5,53E-07	2,34E-03	0.134	*C21orf33*	12489	intergenic	-0.185	-0.44	*PFKL*	NA	NA	NA	FALSE		
chr7:75280121	5,79E-07	2,38E-03	-0.131	*HIP1*	88163	intron	NA	0.255	*RHBDD2*	NA	NA	NA	FALSE	enhancer	*HIP1*
chr7:154585741	7,44E-07	2,82E-03	-0.187	*LOC100132707*	-134486	intron	NA	-0.198	*RP11-5C23.1*	NA	NA	NA	FALSE		
chr1:43814484	7,64E-07	2,86E-03	-0.132	*CDC20*	-10142	intron	NA	0.274	*MED8*	NA	NA	NA	FALSE		
chr16:88453881	7,87E-07	2,91E-03	0.144	*ZNF469*	-39998	intergenic	NA	-0.175	*ZC3H18*	NA	NA	NA	FALSE		
chr19:18118337	1,09E-06	3,77E-03	-0.124	*ARRDC2*	-640	promoter	-0.074	-0.283	*IFI30*	NA	NA	NA	FALSE		
chr3:71804881	1,06E-06	3,77E-03	0.112	*EIF4E3*	-958	promoter	0.129	0.129	*EIF4E3*	NA	NA	NA	FALSE		
chr6:163570410	1,15E-06	3,87E-03	-0.16	*AK296276*	42431	intron	NA	NA	*NA*	NA	NA	NA	FALSE		
chr7:155832938	1,18E-06	3,92E-03	-0.112	*Mir_598*	-106265	intergenic	NA	NA	*NA*	NA	NA	NA	TRUE		
chr16:2848681	1,25E-06	4,07E-03	0.102	*PRSS41*	197	promoter	NA	0.431	*PRSS21*	NA	NA	NA	FALSE		
chr21:46714732	1,25E-06	4,07E-03	-0.108	*LOC642852*	6753	exon	NA	-0.44	*POFUT2*	NA	NA	NA	FALSE		
chr7:155832817	1,32E-06	4,26E-03	-0.115	*Mir_598*	-106144	intergenic	NA	NA	*NA*	NA	NA	NA	FALSE		
chr9:137674072	1,44E-06	4,55E-03	-0.137	*MIR3689C*	67144	intron	NA	0.183	*FCN1*	NA	NA	NA	FALSE		
chr2:27038313	1,62E-06	4,98E-03	-0.163	*CENPA*	29433	intergenic	NA	-0.212	*TMEM214*	NA	NA	NA	TRUE		
chr2:27038339	1,98E-06	5,73E-03	-0.177	*CENPA*	29459	intergenic	NA	0.191	*AGBL5*	NA	NA	NA	FALSE		
chr8:49309214	2,44E-06	6,40E-03	0.157	*EFCAB1*	338657	intergenic	NA	NA	*NA*	NA	NA	NA	FALSE		
chr4:39569133	2,50E-06	6,47E-03	-0.434	*SMIM14*	16446	intron	NA	-0.238	*LIAS*	NA	NA	NA	FALSE		
chr21:46714810	2,66E-06	6,57E-03	-0.109	*LOC642852*	6831	exon	NA	-0.561	*POFUT2*	NA	NA	NA	FALSE		
chr7:22860983	2,88E-06	6,98E-03	-0.191	*TOMM7*	1489	intron	0.24	0.24	*TOMM7*	NA	NA	NA	FALSE		
chr7:22860985	3,22E-06	7,30E-03	-0.231	*TOMM7*	1487	intron	0.189	-0.24	*FAM126A*	NA	NA	NA	FALSE		
chr7:3169658	3,13E-06	7,30E-03	0.296	*BC038729*	44630	intergenic	NA	0.075	*CARD11*	NA	NA	NA	FALSE		
chr7:64540960	3,23E-06	7,30E-03	-0.147	*BC044608*	679	promoter	NA	0.2	*ZNF273*	NA	NA	NA	FALSE		

chr18:60278521	3,46E-06	7,58E-03	0.105	*DKFZp451A185*	29485	intergenic	NA	0.295	*ZCCHC2*	NA	NA	NA	FALSE		
chr21:47308422	3,80E-06	7,98E-03	-0.208	*PCBP3*	-7700	intron	-0.517	-0.517	*PCBP3*	NA	NA	NA	FALSE		
chr19:48000364	3,89E-06	7,99E-03	-0.109	*NAPA-AS1*	12827	intron	-0.141	-0.141	*NAPA-AS1*	NA	NA	NA	TRUE		
chr16:88453824	4,23E-06	8,34E-03	0.152	*ZNF469*	-40055	intergenic	NA	-0.07	*ZC3H18*	NA	NA	NA	TRUE		
chr2:27038406	4,22E-06	8,34E-03	-0.155	*CENPA*	29526	intergenic	NA	-0.229	*TMEM214*	NA	NA	NA	FALSE		
chr4:186318341	4,31E-06	8,41E-03	-0.168	*ANKRD37*	503	promoter	0.151	0.304	*SNX25*	NA	NA	NA	FALSE		
chr2:198768428	4,56E-06	8,77E-03	0.382	*PLCL1*	99004	intron	0.139	0.139	*PLCL1*	NA	NA	NA	FALSE		
chr2:27038368	4,65E-06	8,88E-03	-0.166	*CENPA*	29488	intergenic	NA	0.17	*AGBL5*	NA	NA	NA	FALSE		
chr4:81128398	4,71E-06	8,92E-03	-0.143	*PRDM8*	9743	intergenic	0.101	0.101	*PRDM8*	cg05474265	0.0471994	3.63078	TRUE		
chr4:186318348	5,16E-06	9,49E-03	-0.18	*ANKRD37*	510	promoter	0.059	0.311	*SNX25*	NA	NA	NA	FALSE		
chr4:6107710	5,62E-06	1,00E-02	-0.14	*JAKMIP1*	88637	intron	NA	NA	*NA*	NA	NA	NA	FALSE		
chr18:77397978	6,34E-06	1,05E-02	0.107	*CTDP1*	-41823	intergenic	-0.357	-0.357	*CTDP1*	NA	NA	NA	FALSE		
chr1:228659024	6,42E-06	1,06E-02	0.166	*Histone3*	-7134	intergenic	NA	0.269	*RNF187*	NA	NA	NA	FALSE		
chr2:129160452	6,93E-06	1,11E-02	-0.244	*HS6ST1*	-84282	intergenic	-0.153	-0.153	*HS6ST1*	NA	NA	NA	FALSE		
chr1:43814660	7,37E-06	1,13E-02	-0.18	*CDC20*	-9966	exon	NA	0.345	*MED8*	NA	NA	NA	FALSE		
chr1:54869222	7,44E-06	1,13E-02	0.156	*SSBP3*	2847	intron	0.121	0.154	*SSBP3-AS1*	NA	NA	NA	FALSE	promoter	*SSBP3*
chr7:98029163	7,37E-06	1,13E-02	-0.105	*BAIAP2L1*	1265	intron	NA	0.453	*BRI3*	NA	NA	NA	FALSE	promoter	*BRI3, BAIAP2L1*
chr9:103238980	9,02E-06	1,30E-02	-0.123	*TMEFF1*	3462	intron	NA	-0.217	*TEX10*	NA	NA	NA	FALSE		
chr16:8960728	9,71E-06	1,36E-02	-0.109	*CARHSP1*	558	promoter	-0.125	0.365	*C16orf72*	NA	NA	NA	FALSE	promoter	*PMM2, CARHSP1*
chr11:134709660	1,02E-05	1,40E-02	0.166	*AK125040*	103823	intergenic	NA	NA	*NA*	NA	NA	NA	FALSE		
chr2:27038448	1,05E-05	1,42E-02	-0.102	*CENPA*	29568	intergenic	NA	-0.342	*MAPRE3*	NA	NA	NA	FALSE		
chr16:33039660	1,13E-05	1,47E-02	-0.135	*IGH*	19009	intron	NA	NA	*NA*	NA	NA	NA	FALSE		
chr19:1336307	1,14E-05	1,48E-02	0.112	*MUM1*	-18669	intergenic	-0.078	-0.216	*GPX4*	NA	NA	NA	FALSE		
chr11:75993457	1,15E-05	1,48E-02	0.168	*TRNA_Pro*	-46518	intergenic	NA	0.206	*PRKRIR*	NA	NA	NA	FALSE		
chr12:9892305	1,15E-05	1,48E-02	-0.108	*CLECL1*	-6411	intergenic	0.063	-0.465	*CLEC2D*	NA	NA	NA	FALSE		
chr14:65690537	1,16E-05	1,48E-02	0.116	*BX161428*	-1473	intergenic	NA	0.44	*MAX*	NA	NA	NA	FALSE		
chr3:193693011	1,16E-05	1,48E-02	-0.118	*DPPA2P3*	19017	intron	NA	0.168	*HES1*	NA	NA	NA	FALSE		
chr2:27038396	1,18E-05	1,50E-02	-0.163	*CENPA*	29516	intergenic	NA	0.205	*AGBL5*	NA	NA	NA	FALSE		
chr1:43814666	1,20E-05	1,51E-02	-0.175	*CDC20*	-9960	exon	NA	0.28	*MED8*	NA	NA	NA	FALSE		
chr8:144808965	1,24E-05	1,55E-02	0.151	*FAM83H*	4106	exon	NA	NA	*FAM83H*	cg09380067	0.012695273	4.020182	TRUE		
chr5:131607611	1,28E-05	1,58E-02	0.154	*PDLIM4*	14262	intron	NA	-0.326	*C5orf56*	NA	NA	NA	FALSE		
chr7:22861068	1,28E-05	1,58E-02	-0.143	*TOMM7*	1404	intron	0.142	-0.27	*FAM126A*	NA	NA	NA	FALSE		
chr21:47307692	1,33E-05	1,59E-02	-0.171	*PCBP3*	-8430	intron	-0.591	-0.591	*PCBP3*	NA	NA	NA	FALSE		
chr9:2211535	1,33E-05	1,59E-02	-0.564	*SMARCA2*	51273	intergenic	0.001	0.206	*RP11-264I13.2*	NA	NA	NA	FALSE		
chr19:46915445	1,55E-05	1,74E-02	0.143	*CCDC8*	1475	exon	NA	-0.253	*GNG8*	NA	NA	NA	FALSE		
chr21:46714738	1,57E-05	1,74E-02	-0.125	*LOC642852*	6759	exon	NA	-0.475	*POFUT2*	NA	NA	NA	FALSE		
chr19:1336246	1,68E-05	1,82E-02	0.11	*MUM1*	-18730	intergenic	0.08	0.214	*REEP6*	NA	NA	NA	FALSE		
chr10:71801791	1,74E-05	1,83E-02	-0.122	*H2AFY2*	-10566	intergenic	-0.06	0.32	*PPA1*	NA	NA	NA	FALSE		
chr14:65542789	1,76E-05	1,83E-02	-0.488	*LOC100506321*	-13847	exon	NA	0.315	*RP11-840I19.3*	NA	NA	NA	FALSE		
chr19:58571189	1,75E-05	1,83E-02	0.105	*ZNF135*	584	promoter	NA	0.334	*ZNF587B*	NA	NA	NA	FALSE		
chr2:27038329	1,76E-05	1,83E-02	-0.177	*CENPA*	29449	intergenic	NA	-0.189	*MAPRE3*	NA	NA	NA	FALSE		
chr20:3218611	1,78E-05	1,83E-02	-0.108	*SLC4A11*	225	promoter	NA	0.159	*UBOX5*	NA	NA	NA	FALSE		
chr1:43814473	1,83E-05	1,87E-02	-0.112	*CDC20*	-10153	intron	NA	0.333	*MED8*	NA	NA	NA	FALSE		
chr22:45099111	1,82E-05	1,87E-02	-0.102	*PRR5-ARHGAP8*	741	promoter	NA	0.218	*PRR5*	NA	NA	NA	FALSE	promoter	*PRR5*
chr3:96495772	1,86E-05	1,87E-02	-0.167	*EPHA6*	-37653	intergenic	NA	NA	*NA*	NA	NA	NA	FALSE		
chr10:79395265	1,90E-05	1,88E-02	0.108	*KCNMA1*	2313	intron	NA	NA	*NA*	NA	NA	NA	FALSE		
chr7:22860953	1,92E-05	1,89E-02	-0.153	*TOMM7*	1519	intron	0.133	-0.262	*FAM126A*	NA	NA	NA	FALSE		
chr11:67351944	1,97E-05	1,90E-02	-0.103	*GSTP1*	880	promoter	-0.302	-0.302	*GSTP1*	NA	NA	NA	FALSE		
chr1:43814462	2,02E-05	1,92E-02	-0.159	*CDC20*	-10164	intron	NA	0.258	*MED8*	NA	NA	NA	FALSE		
chr8:6884591	2,04E-05	1,94E-02	-0.111	*DEFA11P*	2421	intergenic	NA	NA	*NA*	NA	NA	NA	FALSE		
chr1:43814638	2,06E-05	1,95E-02	-0.19	*CDC20*	-9988	exon	NA	0.295	*MED8*	NA	NA	NA	FALSE		
chr16:88453894	2,25E-05	2,05E-02	0.153	*ZNF469*	-39985	intergenic	NA	-0.198	*ZC3H18*	NA	NA	NA	FALSE		

chr6:163570230	2,32E-05	2,09E-02	-0.158	*AK296276*	42611	intron	NA	NA	*NA*	NA	NA	NA	FALSE		
chr9:137673998	2,32E-05	2,09E-02	-0.16	*MIR3689C*	67218	intron	NA	0.219	*FCN1*	NA	NA	NA	FALSE		
chr19:10736170	2,36E-05	2,12E-02	-0.145	*SLC44A2*	0	promoter	-0.081	0.226	*PDE4A*	NA	NA	NA	FALSE		
chr6:163570371	2,39E-05	2,14E-02	-0.141	*AK296276*	42470	intron	NA	NA	*NA*	NA	NA	NA	TRUE		
chr4:961400	2,45E-05	2,18E-02	0.221	*DGKQ*	5949	exon	0.342	0.342	*DGKQ*	NA	NA	NA	FALSE		
chr20:55787524	2,78E-05	2,43E-02	-0.112	*BMP7*	54184	intron	NA	0.185	*RBM38*	NA	NA	NA	FALSE	enhancer	*BMP7*
chr3:118892496	2,85E-05	2,47E-02	0.166	*UPK1B*	73	promoter	NA	-0.033	*B4GALT4*	NA	NA	NA	FALSE		
chr11:67351952	2,88E-05	2,49E-02	-0.102	*GSTP1*	888	promoter	-0.433	-0.433	*GSTP1*	NA	NA	NA	FALSE		
chr22:31032485	2,98E-05	2,53E-02	0.143	*SLC35E4*	694	promoter	NA	-0.199	*PES1*	NA	NA	NA	FALSE		
chr3:196694100	2,99E-05	2,53E-02	-0.101	*PIGZ*	1605	intron	NA	0.408	*NCBP2-AS2*	NA	NA	NA	FALSE		
chr9:137674025	3,04E-05	2,55E-02	-0.153	*MIR3689C*	67191	intron	NA	0.319	*FCN1*	NA	NA	NA	FALSE		
chr1:2989648	3,14E-05	2,61E-02	-0.105	*PRDM16*	3908	intron	NA	NA	*NA*	NA	NA	NA	FALSE	promoter	*PRDM16*
chr6:26305203	3,17E-05	2,63E-02	0.11	*TRNA_Ser*	599	promoter	NA	0.253	*BTN3A3*	NA	NA	NA	FALSE	enhancer	*HIST1H3E, ZNF322*
chr19:46915553	3,21E-05	2,63E-02	0.152	*CCDC8*	1367	exon	NA	-0.179	*PPP5C*	NA	NA	NA	FALSE		
chr8:60032942	3,20E-05	2,63E-02	0.113	*TOX*	-1176	intergenic	0.011	-0.314	*RP11-25K19.1*	NA	NA	NA	FALSE		
chr14:19889020	3,37E-05	2,68E-02	-0.119	*LINC00516*	-5349	intron	NA	NA	*NA*	NA	NA	NA	FALSE		
chr19:46774424	3,36E-05	2,68E-02	0.16	*HIF3A*	-25879	intron	NA	-0.166	*PPP5C*	NA	NA	NA	FALSE	enhancer	*HIF3A*
chr19:37807939	3,74E-05	2,79E-02	0.163	*HKR1*	-874	promoter	-0.32	-0.387	*ZNF540*	NA	NA	NA	FALSE		
chr10:126475023	3,75E-05	2,79E-02	0.129	*FAM53B*	3885	intron	0.285	0.285	*FAM53B*	NA	NA	NA	FALSE		
chr8:60032900	3,94E-05	2,88E-02	0.103	*TOX*	-1134	intergenic	-0.157	-0.434	*RP11-25K19.1*	NA	NA	NA	FALSE		
chr20:62570570	4,02E-05	2,92E-02	0.151	*MIR1914*	2328	intergenic	NA	0.271	*RGS19*	NA	NA	NA	FALSE		
chr4:132897079	4,15E-05	3,00E-02	-0.103	*BC131768*	247828	intergenic	NA	NA	*NA*	NA	NA	NA	FALSE		
chr9:137673894	4,31E-05	3,07E-02	-0.18	*MIR3689C*	67322	intron	NA	0.378	*FCN1*	NA	NA	NA	TRUE		
chr17:72916021	4,45E-05	3,13E-02	0.174	*USH1G*	3331	exon	NA	0.367	*ATP5H*	NA	NA	NA	FALSE		
chr4:962384	4,66E-05	3,25E-02	0.136	*DGKQ*	4965	intron	0.508	0.508	*DGKQ*	NA	NA	NA	FALSE		
chr16:2848679	4,69E-05	3,26E-02	0.144	*PRSS41*	195	promoter	NA	0.457	*PRSS21*	NA	NA	NA	FALSE		
chr6:8436263	4,79E-05	3,30E-02	0.175	*LOC100506207*	409	promoter	NA	-0.035	*SLC35B3*	NA	NA	NA	TRUE		
chr19:37807753	5,14E-05	3,48E-02	0.181	*HKR1*	-1060	intron	-0.278	-0.345	*ZNF383*	NA	NA	NA	FALSE		
chr6:13860701	5,17E-05	3,48E-02	-0.171	*Mir_548*	45070	intergenic	NA	-0.204	*NA*	NA	NA	NA	FALSE		
chr15:20374137	5,31E-05	3,54E-02	-0.158	*BC107108*	11451	intergenic	NA	NA	*NA*	NA	NA	NA	FALSE		
chr10:79395468	5,34E-05	3,55E-02	0.108	*KCNMA1*	2110	intron	NA	NA	*NA*	NA	NA	NA	FALSE		
chr15:98195837	5,62E-05	3,68E-02	0.113	*BC024169*	-114382	intergenic	NA	NA	*NA*	NA	NA	NA	FALSE		
chr22:39633541	5,73E-05	3,72E-02	0.147	*PDGFB*	3374	intron	NA	-0.225	*APOBEC3D*	NA	NA	NA	FALSE		
chr22:26323710	5,99E-05	3,79E-02	-0.273	*MYO18B*	-27435	intron	NA	NA	*NA*	NA	NA	NA	FALSE		
chr1:43814632	6,11E-05	3,82E-02	-0.19	*CDC20*	-9994	exon	NA	0.319	*MED8*	NA	NA	NA	FALSE		
chr17:135302	6,17E-05	3,85E-02	0.118	*RPH3AL*	42069	intron	NA	-0.077	*FAM101B*	NA	NA	NA	FALSE		
chr8:145733678	6,30E-05	3,90E-02	0.109	*MFSD3*	-874	promoter	0.156	0.201	*RECQL4*	NA	NA	NA	FALSE	promoter	*MFSD3*
chr12:92029279	6,33E-05	3,91E-02	-0.132	*DCN*	-452474	intergenic	NA	NA	*NA*	NA	NA	NA	FALSE		
chr4:160303530	7,08E-05	4,17E-02	0.133	*RAPGEF2*	114534	intergenic	-0.055	NA	*NA*	NA	NA	NA	FALSE		
chr14:101195081	7,23E-05	4,18E-02	0.172	*DLK1*	1881	intron	NA	NA	*NA*	NA	NA	NA	FALSE		
chr4:961599	7,29E-05	4,18E-02	0.127	*DGKQ*	5750	intron	0.619	0.619	*DGKQ*	NA	NA	NA	FALSE		
chr7:22861019	7,40E-05	4,22E-02	-0.134	*TOMM7*	1453	intron	0.174	-0.325	*FAM126A*	NA	NA	NA	FALSE		
chr18:60278825	7,44E-05	4,23E-02	0.123	*DKFZp451A185*	29789	intergenic	NA	0.228	*ZCCHC2*	NA	NA	NA	FALSE		
chr18:77398374	7,44E-05	4,23E-02	0.121	*CTDP1*	-41427	intergenic	0.056	0.095	*NFATC1*	NA	NA	NA	FALSE		
chr22:49447830	7,62E-05	4,30E-02	0.244	*LOC100128946*	185250	intergenic	NA	NA	*NA*	NA	NA	NA	FALSE		
chr7:154585853	7,74E-05	4,36E-02	-0.164	*LOC100132707*	-134374	exon	NA	-0.313	*RP11-5C23.1*	NA	NA	NA	FALSE		
chr14:103227458	7,89E-05	4,41E-02	-0.134	*TRAF3*	-16358	intergenic	0.335	0.335	*TRAF3*	NA	NA	NA	FALSE		
chr20:47013321	8,38E-05	4,58E-02	-0.173	*LINC00494*	18933	intergenic	NA	NA	*NA*	NA	NA	NA	FALSE		
chr19:37807898	8,62E-05	4,66E-02	0.138	*HKR1*	-915	promoter	-0.237	-0.408	*ZNF383*	NA	NA	NA	FALSE		
chr8:42052734	8,74E-05	4,71E-02	-0.156	*PLAT*	-2006	intron	NA	0.288	*KAT6A*	NA	NA	NA	FALSE		
chr1:178456076	8,93E-05	4,76E-02	0.112	*TEX35*	-26136	intergenic	NA	-0.027	*RP11-428K3.1*	NA	NA	NA	FALSE		
chr18:60278823	8,92E-05	4,76E-02	0.127	*DKFZp451A185*	29787	intergenic	NA	0.233	*ZCCHC2*	NA	NA	NA	FALSE		

chr21:45565995	8,95E-05	4,76E-02	0.146	*C21orf33*	12503	intergenic	-0.056	-0.375	*PFKL*	NA	NA	NA	FALSE
chr22:25174843	9,08E-05	4,79E-02	-0.121	*PIWIL3*	-4157	intergenic	NA	-0.443	*GGT1*	NA	NA	NA	FALSE
chr21:46677404	9,13E-05	4,79E-02	0.231	*POFUT2*	19922	intron	0.013	-0.167	*ADARB1*	NA	NA	NA	FALSE
chr12:32626644	9,79E-05	4,94E-02	-0.28	*FGD4*	-12262	intergenic	NA	-0.325	*DNM1L*	NA	NA	NA	FALSE
chr16:89150862	9,85E-05	4,94E-02	-0.16	*ACSF3*	-9355	intergenic	-0.054	0.188	*AC137932.5*	NA	NA	NA	FALSE
chr19:37807937	9,93E-05	4,95E-02	0.176	*HKR1*	-876	promoter	-0.228	-0.404	*ZNF570*	NA	NA	NA	FALSE
chr1:178456064	1,08E-04	5,18E-02	0.103	*TEX35*	-26148	intergenic	NA	-0.039	*RALGPS2*	NA	NA	NA	FALSE
chr13:20711574	1,08E-04	5,18E-02	0.137	*GJA3*	5854	intergenic	NA	0.224	*GJB6*	NA	NA	NA	TRUE
chr8:144809551	1,09E-04	5,19E-02	0.202	*FAM83H*	3520	exon	NA	-0.467	*NAPRT1*	NA	NA	NA	FALSE
chr4:132897000	1,11E-04	5,24E-02	-0.139	*BC131768*	247749	intergenic	NA	NA	*NA*	NA	NA	NA	FALSE
chr7:22861114	1,12E-04	5,24E-02	-0.19	*TOMM7*	1358	intron	0.217	-0.337	*FAM126A*	NA	NA	NA	FALSE
chr16:89050592	1,15E-04	5,34E-02	0.162	*CBFA2T3*	-7089	intergenic	NA	0.221	*CDT1*	NA	NA	NA	FALSE
chr22:49447896	1,16E-04	5,34E-02	0.257	*LOC100128946*	185316	intergenic	NA	NA	*NA*	NA	NA	NA	FALSE
chr19:37807945	1,18E-04	5,38E-02	0.121	*HKR1*	-868	promoter	-0.241	-0.437	*ZNF383*	NA	NA	NA	FALSE
chr16:32290350	1,21E-04	5,46E-02	-0.108	*LOC390705*	10953	intergenic	NA	NA	*NA*	NA	NA	NA	FALSE
chr20:47013360	1,21E-04	5,46E-02	-0.119	*LINC00494*	18972	intergenic	NA	NA	*NA*	NA	NA	NA	FALSE
chr4:962204	1,22E-04	5,46E-02	0.226	*DGKQ*	5145	intron	0.43	0.43	*DGKQ*	NA	NA	NA	FALSE
chr6:163570381	1,22E-04	5,46E-02	-0.161	*AK296276*	42460	intron	NA	NA	*NA*	NA	NA	NA	FALSE
chr12:133414426	1,26E-04	5,56E-02	0.16	*GOLGA3*	-9001	intergenic	-0.098	-0.302	*CHFR*	NA	NA	NA	FALSE
chr18:60278889	1,25E-04	5,56E-02	0.144	*DKFZp451A185*	29853	intergenic	NA	0.209	*ZCCHC2*	NA	NA	NA	FALSE
chr16:89150998	1,29E-04	5,64E-02	-0.143	*ACSF3*	-9219	intergenic	-0.238	0.326	*TRAPPC2L*	NA	NA	NA	FALSE
chr7:76129434	1,29E-04	5,64E-02	-0.165	*DTX2*	317	promoter	-0.471	-0.539	*UPK3B*	NA	NA	NA	FALSE
chr17:15517405	1,38E-04	5,86E-02	0.298	*CDRT1*	5614	intron	NA	0.318	*ZNF286A*	NA	NA	NA	FALSE
chr21:46714776	1,42E-04	5,88E-02	-0.123	*LOC642852*	6797	exon	NA	-0.497	*POFUT2*	NA	NA	NA	FALSE
chr17:72916084	1,45E-04	5,96E-02	0.173	*USH1G*	3268	exon	NA	0.378	*ATP5H*	NA	NA	NA	FALSE
chr9:136075442	1,47E-04	6,01E-02	0.111	*OBP2B*	9187	intergenic	NA	-0.308	*CACFD1*	NA	NA	NA	FALSE
chr6:163570616	1,48E-04	6,05E-02	-0.117	*AK296276*	42225	intron	NA	NA	*NA*	NA	NA	NA	FALSE
chr21:46714693	1,53E-04	6,19E-02	-0.104	*LOC642852*	6714	exon	NA	-0.414	*POFUT2*	NA	NA	NA	FALSE
chr7:128561507	1,61E-04	6,37E-02	0.107	*KCP*	-10735	intergenic	NA	0.374	*CALU*	NA	NA	NA	FALSE
chr2:113192585	1,72E-04	6,65E-02	0.11	*RGPD8*	-524	promoter	0.164	0.311	*SLC20A1*	NA	NA	NA	FALSE
chr4:961427	1,72E-04	6,66E-02	0.185	*DGKQ*	5922	exon	0.259	0.259	*DGKQ*	NA	NA	NA	FALSE
chr22:49447907	1,83E-04	6,96E-02	0.288	*LOC100128946*	185327	intergenic	NA	NA	*NA*	NA	NA	NA	FALSE
chr16:32289963	1,84E-04	6,96E-02	-0.108	*LOC390705*	11340	intergenic	NA	NA	*NA*	NA	NA	NA	FALSE
chr4:8890133	1,89E-04	7,06E-02	0.151	*HMX1*	-16591	intergenic	NA	NA	*NA*	NA	NA	NA	FALSE
chr12:125242855	2,03E-04	7,39E-02	0.494	*JB074994*	-39454	intergenic	NA	-0.214	*DHX37*	NA	NA	NA	FALSE
chr22:49442398	2,03E-04	7,40E-02	-0.109	*LOC100128946*	179818	intergenic	NA	NA	*NA*	NA	NA	NA	FALSE
chr10:79376361	2,11E-04	7,56E-02	-0.319	*JB149426*	2204	intron	NA	NA	*NA*	NA	NA	NA	FALSE
chr19:1466099	2,13E-04	7,59E-02	0.176	*C19orf25*	13130	exon	0.132	0.287	*ATP5D*	NA	NA	NA	FALSE
chr5:3606057	2,14E-04	7,61E-02	0.101	*IRX1*	9891	intergenic	NA	NA	*NA*	NA	NA	NA	FALSE
chr18:60278808	2,15E-04	7,61E-02	0.11	*DKFZp451A185*	29772	intergenic	NA	0.406	*ZCCHC2*	NA	NA	NA	FALSE
chr19:58571386	2,16E-04	7,64E-02	0.107	*ZNF135*	781	promoter	NA	-0.327	*ZNF417*	NA	NA	NA	FALSE
chr19:37807657	2,23E-04	7,76E-02	0.136	*HKR1*	-1156	intron	-0.089	-0.267	*ZNF570*	NA	NA	NA	FALSE
chr21:47307758	2,25E-04	7,82E-02	-0.21	*PCBP3*	-8364	intron	-0.537	-0.537	*PCBP3*	NA	NA	NA	FALSE
chr1:149287263	2,30E-04	7,91E-02	-0.115	*BC023516*	-188	promoter	NA	0.15	*NA*	NA	NA	NA	FALSE
chr12:92029135	2,33E-04	7,93E-02	-0.138	*DCN*	-452330	intergenic	NA	NA	*NA*	NA	NA	NA	FALSE
chr13:99803949	2,37E-04	8,00E-02	0.149	*UBAC2*	-48730	intergenic	0.015	-0.271	*GPR18*	NA	NA	NA	FALSE
chr2:113192407	2,38E-04	8,02E-02	0.104	*RGPD8*	-346	promoter	-0.079	0.264	*TTL*	NA	NA	NA	FALSE
chr4:81128366	2,54E-04	8,32E-02	-0.109	*PRDM8*	9711	intergenic	0.093	-0.159	*ANTXR2*	NA	NA	NA	FALSE
chr3:194705981	2,55E-04	8,33E-02	-0.102	*XXYLT1*	136944	intergenic	0.043	NA	*NA*	NA	NA	NA	FALSE
chr7:158111329	2,57E-04	8,36E-02	0.128	*MIR595*	214177	intron	NA	NA	*NA*	NA	NA	NA	TRUE
chr22:26323697	2,64E-04	8,48E-02	-0.113	*MYO18B*	-27448	intron	NA	NA	*NA*	NA	NA	NA	FALSE
chr17:72916093	2,67E-04	8,51E-02	0.181	*USH1G*	3259	exon	NA	0.455	*KCTD2*	NA	NA	NA	FALSE

chr22:19751804	2,70E-04	8,56E-02	0.115	*TBX1*	7580	exon	NA	0.085	*COMT*	NA	NA	NA	FALSE	enhancer	*RPL7AP70, TBX1, RPL8P5*
chr6:163570442	2,71E-04	8,56E-02	-0.169	*AK296276*	42399	intron	NA	NA	*NA*	NA	NA	NA	FALSE		
chr16:89050558	2,74E-04	8,60E-02	0.14	*CBFA2T3*	-7055	intergenic	NA	0.119	*CDT1*	NA	NA	NA	FALSE		
chr22:17199120	2,78E-04	8,69E-02	-0.187	*BC038197*	30209	intergenic	NA	-0.088	*TPTEP1*	NA	NA	NA	FALSE		
chr19:37807762	2,79E-04	8,70E-02	0.139	*HKR1*	-1051	intron	-0.182	-0.344	*ZNF540*	NA	NA	NA	FALSE		
chr21:47307812	2,83E-04	8,77E-02	-0.273	*PCBP3*	-8310	intron	-0.49	-0.49	*PCBP3*	NA	NA	NA	FALSE		
chr21:47307815	2,83E-04	8,77E-02	-0.203	*PCBP3*	-8307	intron	-0.495	-0.495	*PCBP3*	NA	NA	NA	FALSE		
chr8:2670073	2,84E-04	8,77E-02	-0.116	*AK128880*	-84117	intergenic	NA	NA	*NA*	NA	NA	NA	FALSE		
chr12:116978050	2,87E-04	8,80E-02	-0.238	*LINC00173*	6812	intergenic	NA	-0.11	*C12orf49*	NA	NA	NA	FALSE	enhancer	*MED13L, LINC00173*
chr4:81128495	2,93E-04	8,95E-02	-0.126	*PRDM8*	9840	intergenic	0.024	-0.037	*ANTXR2*	NA	NA	NA	FALSE		
chr22:44530074	2,93E-04	8,95E-02	0.16	*TRNA_SeC*	-16463	intron	NA	0.064	*PARVB*	NA	NA	NA	FALSE		
chr6:110904825	3,00E-04	9,07E-02	0.452	*CDK19*	59697	intergenic	0.158	0.158	*CDK19*	NA	NA	NA	FALSE		
chr21:46714714	3,09E-04	9,18E-02	-0.103	*LOC642852*	6735	exon	NA	-0.365	*POFUT2*	NA	NA	NA	FALSE		
chr15:98195852	3,21E-04	9,36E-02	0.114	*BC024169*	-114397	intergenic	NA	NA	*NA*	NA	NA	NA	FALSE		
chr19:46915556	3,22E-04	9,37E-02	0.146	*CCDC8*	1364	exon	NA	0.289	*CALM3*	NA	NA	NA	FALSE		
chr21:47307753	3,25E-04	9,44E-02	-0.227	*PCBP3*	-8369	intron	-0.489	-0.489	*PCBP3*	NA	NA	NA	FALSE		
chr8:144788642	3,32E-04	9,52E-02	0.227	*CCDC166*	1638	intron	NA	-0.238	*RP11-661A12.7*	NA	NA	NA	FALSE		
chr5:131607542	3,33E-04	9,56E-02	0.142	*PDLIM4*	14193	exon	NA	-0.41	*C5orf56*	NA	NA	NA	FALSE		
chr7:76129418	3,35E-04	9,59E-02	-0.164	*DTX2*	301	promoter	-0.416	-0.466	*UPK3B*	NA	NA	NA	FALSE		
chr21:47307737	3,42E-04	9,71E-02	-0.223	*PCBP3*	-8385	intron	-0.476	-0.476	*PCBP3*	NA	NA	NA	FALSE		
chr21:47308375	3,46E-04	9,76E-02	-0.244	*PCBP3*	-7747	intron	-0.537	-0.537	*PCBP3*	NA	NA	NA	FALSE		
chr1:228659210	3,49E-04	9,79E-02	0.109	*Histone3*	-7320	intergenic	NA	0.154	*RHOU*	NA	NA	NA	FALSE		
chr1:55246878	3,54E-04	9,87E-02	0.123	*PARS2*	-16653	exon	NA	0.196	*TTC22*	NA	NA	NA	FALSE		

Standalone differentially methylated CpGs, which are not part of differentially methylated regions, are marked with red color.

ESM Table 4. Differentially methylated CpGs identified in the CD8$^+$ T cell fraction between cases and controls in all longitudinal samples

Methylation difference				Nearest gene			Methylation–expression correlation analysis			eQTM analysis				GeneHancer database	
CpG site	P value	FDR	Methylation difference	Nearest gene	Distance to nearest gene	Genomic part	Nearest gene correlation, Spearman rho	The highest observed correlation, Spearman rho	Correlating gene	CpG name	eQTM, FDR	Overall Z Score	CpG found on 450K	Genehancer	Genes possibly regulated by standalone DMCs
chr16:32289963	5,21E-11	1,27E-05	-0.13	LOC390705	11340	intergenic	NA	NA	NA	NA	NA	NA	FALSE		
chr2:27038339	2,78E-10	4,31E-05	-0.186	CENPA	29459	intergenic	NA	-0.092	TMEM214	NA	NA	NA	FALSE		
chr9:79557441	5,37E-10	7,04E-05	0.154	PRUNE2	-36439	intergenic	NA	-0.066	VPS13A	NA	NA	NA	FALSE		
chr14:45343058	8,55E-10	9,11E-05	-0.207	C14orf28	-23449	intergenic	0.085	-0.259	FAM179B	NA	NA	NA	FALSE		
chr16:433855	1,14E-09	1,08E-04	-0.13	LOC100134368	1616	intron	NA	-0.538	TMEM8A	NA	NA	NA	FALSE		
chr22:39633525	1,14E-09	1,08E-04	0.152	PDGFB	3390	intron	NA	-0.222	CBX7	NA	NA	NA	FALSE		
chr7:3169658	3,27E-09	2,65E-04	0.191	BC038729	44630	intergenic	NA	0.018	CARD11	NA	NA	NA	FALSE		
chr16:58534501	6,77E-09	4,44E-04	0.121	NDRG4	456	promoter	NA	-0.229	GOT2	NA	NA	NA	FALSE		
chr1:145385334	1,02E-08	5,82E-04	0.165	TRNA_Asn	-395	promoter	NA	-0.419	PIAS3	NA	NA	NA	TRUE		
chr16:8960779	1,59E-08	8,76E-04	-0.118	CARHSP1	507	promoter	0.128	0.146	TMEM186	NA	NA	NA	FALSE		
chr14:101194894	1,69E-08	8,85E-04	0.258	DLK1	1694	intron	NA	NA	NA	NA	NA	NA	FALSE		
chr17:16649246	1,82E-08	8,85E-04	-0.136	USP32P1	34255	intron	NA	-0.121	ZNF624	NA	NA	NA	TRUE		
chr3:196694364	1,81E-08	8,85E-04	-0.191	PIGZ	1341	intron	NA	-0.183	AC127904.2	NA	NA	NA	FALSE		
chr21:10597321	2,41E-08	1,05E-03	-0.16	AK311573	122	promoter	NA	NA	NA	NA	NA	NA	FALSE		
chr8:144788642	3,95E-08	1,38E-03	0.267	CCDC166	1638	intron	NA	0.216	GSDMD	NA	NA	NA	FALSE		
chr2:129160452	4,46E-08	1,52E-03	-0.213	HS6ST1	-84282	intergenic	-0.08	-0.08	HS6ST1	NA	NA	NA	FALSE		
chr2:27038368	7,85E-08	2,35E-03	-0.171	CENPA	29488	intergenic	NA	-0.105	TMEM214	NA	NA	NA	FALSE		
chr15:20374137	1,12E-07	2,99E-03	-0.105	BC107108	11451	intergenic	NA	NA	NA	NA	NA	NA	FALSE		
chr10:35102729	1,19E-07	3,13E-03	-0.109	PARD3	1525	intron	NA	NA	NA	NA	NA	NA	FALSE	promoter	PARD3, PARD3-AS1
chr5:177629492	1,23E-07	3,14E-03	-0.15	HNRNPAB	-2016	intergenic	0.041	0.267	N4BP3	NA	NA	NA	FALSE		
chr1:145385336	1,35E-07	3,40E-03	0.135	TRNA_Asn	-393	promoter	NA	-0.43	PIAS3	NA	NA	NA	FALSE		
chr12:116978050	1,43E-07	3,47E-03	-0.229	LINC00173	6812	intergenic	NA	0.063	C12orf49	NA	NA	NA	FALSE		
chr16:3116115	1,68E-07	3,82E-03	-0.141	IL32	332	promoter	-0.117	0.285	ZNF200	NA	NA	NA	FALSE		
chr5:3605712	2,16E-07	4,67E-03	0.139	IRX1	9546	intergenic	NA	NA	NA	NA	NA	NA	FALSE		
chr3:196694286	2,94E-07	5,90E-03	-0.183	PIGZ	1419	intron	NA	-0.218	AC127904.2	NA	NA	NA	FALSE		
chr18:844167	3,13E-07	6,14E-03	-0.134	YES1	-31626	intergenic	0.206	-0.394	ENOSF1	NA	NA	NA	FALSE		
chr16:3116143	3,26E-07	6,25E-03	-0.13	IL32	360	promoter	-0.101	-0.334	ZNF213-AS1	NA	NA	NA	FALSE		
chr2:27038313	3,31E-07	6,27E-03	-0.178	CENPA	29433	intergenic	NA	-0.074	TMEM214	NA	NA	NA	TRUE		
chr3:196694318	3,38E-07	6,33E-03	-0.166	PIGZ	1387	intron	NA	-0.235	AC127904.2	NA	NA	NA	FALSE		
chr7:2060040	4,39E-07	7,92E-03	0.13	MAD1L1	-79856	intron	0.071	-0.116	FTSJ2	NA	NA	NA	TRUE		
chr1:145385315	4,68E-07	8,23E-03	0.206	TRNA_Asn	-414	promoter	NA	-0.427	PIAS3	NA	NA	NA	FALSE		
chr16:121845	5,11E-07	8,81E-03	-0.162	RHBDF1	785	promoter	NA	-0.271	LUC7L	NA	NA	NA	FALSE	promoter	PRL3, MPG, RHBDF1
chr10:1416953	6,03E-07	1,00E-02	-0.147	ADARB2-AS1	-151872	intron	NA	NA	NA	NA	NA	NA	FALSE		
chr20:2280332	6,05E-07	1,00E-02	0.117	TGM3	3721	intron	NA	0.229	STK35	NA	NA	NA	FALSE		
chr6:158631165	6,72E-07	1,06E-02	-0.161	GTF2H5	41788	intergenic	-0.083	-0.21	SYNJ2	NA	NA	NA	FALSE		
chr20:2690396	8,53E-07	1,26E-02	-0.15	EBF4	16874	intron	0.034	0.233	VPS16	NA	NA	NA	FALSE		
chr22:49447718	8,76E-07	1,28E-02	0.118	LOC100128946	185138	intergenic	NA	NA	NA	NA	NA	NA	FALSE		
chr10:118885566	9,29E-07	1,34E-02	-0.144	KIAA1598	532	promoter	NA	-0.143	SLC18A2	NA	NA	NA	FALSE		
chr11:118842764	9,67E-07	1,37E-02	0.118	FOXR1	349	promoter	NA	0.316	RPS25	NA	NA	NA	FALSE		
chr2:113192534	1,08E-06	1,50E-02	0.128	RGPD8	-473	promoter	-0.127	-0.127	RGPD8	NA	NA	NA	FALSE		
chr17:42733527	1,11E-06	1,54E-02	-0.166	C17orf104	-235	promoter	NA	-0.109	DBF4B	NA	NA	NA	TRUE		
chr7:128708938	1,25E-06	1,62E-02	0.236	TPI1P2	13663	intergenic	NA	-0.281	ODCP	NA	NA	NA	FALSE		
chr9:139427025	1,36E-06	1,71E-02	0.173	MIR4673	-12948	intron	NA	0.33	RP11-611D20.2	NA	NA	NA	FALSE	enhancer	SEC16A, NOTCH1
chr17:80057621	1,39E-06	1,73E-02	0.179	FASN	-1516	intergenic	0.053	-0.332	ARHGDIA	NA	NA	NA	FALSE		
chr4:123286282	1,54E-06	1,87E-02	-0.123	ADAD1	-13839	intergenic	NA	-0.037	KIAA1109	NA	NA	NA	FALSE		
chr7:2903150	1,63E-06	1,95E-02	0.191	GNA12	-19192	intergenic	0.052	-0.11	CARD11	NA	NA	NA	FALSE		
chr6:158631123	1,83E-06	2,06E-02	-0.163	GTF2H5	41746	intergenic	-0.04	-0.219	SYNJ2	NA	NA	NA	FALSE		
chr5:178156736	1,91E-06	2,10E-02	-0.131	ZNF354A	968	promoter	0.091	-0.136	ZNF354B	NA	NA	NA	FALSE		
chr7:2903323	2,06E-06	2,20E-02	0.164	GNA12	-19365	intergenic	-0.28	-0.28	GNA12	NA	NA	NA	FALSE		

chr9:70937458	2,14E-06	2,24E-02	-0.156	*FOXD4L3*	19677	intergenic	NA	NA	*NA*	NA	NA	NA	FALSE		
chr19:56019597	2,18E-06	2,27E-02	-0.576	*SSC5D*	19729	intron	NA	0.233	*ZNF579*	NA	NA	NA	FALSE		
chr4:184602729	2,56E-06	2,56E-02	0.37	*TRAPPC11*	-2273	intron	0.092	-0.403	*RWDD4*	NA	NA	NA	FALSE		
chr6:158631168	2,62E-06	2,58E-02	-0.155	*GTF2H5*	41791	intergenic	-0.006	-0.199	*SYNJ2*	NA	NA	NA	FALSE		
chr19:4058128	2,74E-06	2,66E-02	0.156	*ZBTB7A*	7360	intron	-0.238	-0.245	*DAPK3*	NA	NA	NA	FALSE	promoter	*ZBTB7A*
chr8:11352019	3,07E-06	2,84E-02	-0.117	*BLK*	500	promoter	-0.429	-0.429	*BLK*	NA	NA	NA	FALSE	promoter	*FAM167A*
chr3:96495764	3,56E-06	3,13E-02	-0.156	*EPHA6*	-37661	intergenic	NA	NA	*NA*	NA	NA	NA	FALSE		
chr2:2923622	3,74E-06	3,23E-02	-0.152	*AK095310*	206177	intron	NA	NA	*NA*	NA	NA	NA	FALSE		
chr13:19183653	3,91E-06	3,34E-02	-0.179	*LINC00417*	130587	intergenic	NA	NA	*NA*	NA	NA	NA	FALSE		
chr19:54677584	4,45E-06	3,67E-02	0.128	*TMC4*	-641	promoter	NA	-0.231	*TSEN34*	NA	NA	NA	FALSE		
chr19:1466187	5,02E-06	3,94E-02	0.104	*C19orf25*	13042	exon	0.081	0.172	*REEP6*	NA	NA	NA	FALSE		
chr17:956622	5,07E-06	3,95E-02	-0.171	*Metazoa_SRP*	9015	intron	NA	0.103	*ABR*	NA	NA	NA	FALSE		
chr10:135092506	5,12E-06	3,96E-02	0.179	*ADAM8*	-2100	exon	-0.52	-0.52	*ADAM8*	NA	NA	NA	FALSE		
chr2:27038329	5,35E-06	4,09E-02	-0.184	*CENPA*	29449	intergenic	NA	-0.077	*TMEM214*	NA	NA	NA	FALSE		
chr16:8960607	5,46E-06	4,16E-02	-0.113	*CARHSP1*	679	promoter	0.047	0.287	*C16orf72*	NA	NA	NA	FALSE		
chr14:57019261	6,27E-06	4,44E-02	-0.159	*TMEM260*	-27077	intergenic	0.072	0.072	*TMEM260*	NA	NA	NA	FALSE		
chr17:80272875	6,23E-06	4,44E-02	0.143	*CD7*	2606	exon	-0.202	-0.228	*FASN*	NA	NA	NA	FALSE		
chr14:101195081	6,54E-06	4,55E-02	0.136	*DLK1*	1881	intron	NA	NA	*NA*	NA	NA	NA	FALSE		
chr21:46654292	7,23E-06	4,88E-02	-0.105	*C21orf89*	27	promoter	NA	-0.263	*POFUT2*	NA	NA	NA	FALSE		
chr21:10990482	7,39E-06	4,96E-02	-0.156	*TPTE*	439	promoter	NA	NA	*NA*	NA	NA	NA	FALSE		
chr8:11813054	7,81E-06	5,16E-02	0.131	*DEFB136*	19055	intergenic	NA	0.404	*FAM85A*	NA	NA	NA	FALSE		
chr12:128827635	8,25E-06	5,35E-02	-0.134	*MIR3612*	49000	intron	NA	NA	*NA*	NA	NA	NA	FALSE		
chr2:27038406	8,32E-06	5,38E-02	-0.179	*CENPA*	29526	intergenic	NA	-0.102	*TMEM214*	NA	NA	NA	FALSE		
chr19:37807788	8,56E-06	5,49E-02	0.169	*HKR1*	-1025	intron	-0.039	0.256	*ZNF569*	NA	NA	NA	FALSE		
chr3:196694243	9,15E-06	5,76E-02	-0.173	*PIGZ*	1462	intron	NA	-0.247	*AC127904.2*	NA	NA	NA	FALSE		
chr14:65839593	9,59E-06	5,85E-02	0.113	*MIR4708*	-37693	intergenic	NA	-0.16	*FUT8*	NA	NA	NA	FALSE		
chr17:25979102	9,47E-06	5,85E-02	-0.157	*DQ586005*	-15202	intergenic	NA	-0.167	*LGALS9*	NA	NA	NA	FALSE		
chr9:139426336	9,53E-06	5,85E-02	0.142	*MIR4673*	-12259	intron	NA	0.154	*SEC16A*	NA	NA	NA	FALSE	enhancer	*SEC16A, NOTCH1*
chr18:40176497	9,80E-06	5,93E-02	-0.136	*Mir_544*	-203586	intron	NA	NA	*NA*	NA	NA	NA	FALSE		
chr2:27038408	9,96E-06	5,98E-02	-0.176	*CENPA*	29528	intergenic	NA	-0.074	*TMEM214*	NA	NA	NA	FALSE		
chr5:131822736	1,03E-05	6,07E-02	0.193	*IRF1*	2440	exon	-0.318	-0.325	*AC116366.6*	NA	NA	NA	FALSE		
chr3:118892467	1,04E-05	6,08E-02	0.196	*UPK1B*	44	promoter	NA	0.125	*B4GALT4*	NA	NA	NA	FALSE		
chr4:39569133	1,10E-05	6,28E-02	-0.429	*SMIM14*	16446	intron	NA	0.188	*UBE2K*	NA	NA	NA	FALSE		
chr5:83928	1,12E-05	6,29E-02	0.317	*PLEKHG4B*	-56445	intergenic	NA	-0.175	*SDHA*	NA	NA	NA	FALSE		
chr4:184602661	1,12E-05	6,31E-02	0.371	*TRAPPC11*	-2341	intron	0.104	-0.386	*RWDD4*	NA	NA	NA	FALSE		
chr10:135092554	1,15E-05	6,41E-02	0.137	*ADAM8*	-2148	exon	-0.425	-0.425	*ADAM8*	NA	NA	NA	FALSE		
chr10:55458783	1,24E-05	6,67E-02	0.15	*MBL2*	-927324	intergenic	NA	NA	*NA*	NA	NA	NA	FALSE		
chr7:331219	1,25E-05	6,70E-02	0.106	*LOC100288524*	1085	exon	NA	-0.111	*PDGFA*	NA	NA	NA	FALSE		
chr20:44451904	1,31E-05	6,77E-02	0.15	*TNNC2*	4050	exon	-0.383	-0.383	*TNNC2*	NA	NA	NA	FALSE	enhancer	*WFDC3, PLTP*
chr4:1522024	1,32E-05	6,77E-02	0.211	*AX748388*	55943	intergenic	NA	-0.403	*SLBP*	NA	NA	NA	FALSE		
chr9:46355134	1,29E-05	6,77E-02	-0.112	*Y_RNA*	-7471	intergenic	NA	NA	*NA*	NA	NA	NA	FALSE		
chr16:433927	1,33E-05	6,77E-02	-0.226	*LOC100134368*	1688	intron	NA	-0.255	*TMEM8A*	NA	NA	NA	FALSE		
chr6:158636092	1,33E-05	6,77E-02	-0.209	*GTF2H5*	46715	intergenic	-0.147	-0.298	*SYNJ2*	NA	NA	NA	FALSE		
chr2:37382150	1,34E-05	6,78E-02	0.149	*EIF2AK2*	2041	intron	-0.243	-0.335	*PRKD3*	NA	NA	NA	FALSE	promoter	*EIF2AK2, NDUFAF7*
chr18:74118259	1,43E-05	6,95E-02	-0.156	*ZNF516*	-26001	intron	0.101	0.101	*ZNF516*	NA	NA	NA	FALSE		
chr10:118885541	1,47E-05	7,07E-02	-0.137	*KIAA1598*	557	promoter	NA	0.178	*PDZD8*	NA	NA	NA	FALSE		
chr3:118892305	1,50E-05	7,15E-02	0.204	*UPK1B*	-120	promoter	NA	0.28	*B4GALT4*	NA	NA	NA	TRUE		
chr15:27139216	1,53E-05	7,22E-02	0.146	*GABRA5*	26945	intron	NA	NA	*NA*	NA	NA	NA	FALSE		
chr16:3116120	1,54E-05	7,22E-02	-0.137	*IL32*	337	promoter	-0.154	-0.297	*ZNF213-AS1*	NA	NA	NA	FALSE		
chr16:21200438	1,67E-05	7,58E-02	0.228	*ZP2*	22630	intergenic	NA	0.392	*NPIPB3*	NA	NA	NA	FALSE		
chr1:145385344	1,70E-05	7,66E-02	0.119	*TRNA_Asn*	-385	promoter	NA	-0.46	*PIAS3*	NA	NA	NA	FALSE		
chr2:113192585	1,70E-05	7,66E-02	0.177	*RGPD8*	-524	promoter	-0.193	0.213	*TTL*	NA	NA	NA	FALSE		
chr9:129262301	1,75E-05	7,74E-02	0.121	*Mir_1302*	30948	intron	NA	-0.133	*MVB12B*	NA	NA	NA	FALSE	enhancer	*MVB12B*
chr7:52341721	1,85E-05	8,07E-02	-0.156	*POM121L12*	-761628	intergenic	NA	NA	*NA*	NA	NA	NA	FALSE		
chr19:575524	1,92E-05	8,31E-02	-0.132	*BSG*	3072	intron	-0.223	-0.223	*BSG*	NA	NA	NA	FALSE		

chr10:134597567	1,94E-05	8,34E-02	0.142	*NKX6-2*	1971	intergenic	NA	-0.009	*INPP5A*	NA	NA	NA	FALSE		
chr8:128891631	1,96E-05	8,42E-02	-0.103	*PVT1*	-11204	intron	0	-0.151	*MYC*	NA	NA	NA	FALSE	enhancer	*MIR1204, PVT1*
chr2:113192563	2,18E-05	9,03E-02	0.108	*RGPD8*	-502	promoter	-0.145	-0.145	*RGPD8*	NA	NA	NA	FALSE		
chr4:190906058	2,46E-05	9,67E-02	-0.137	*TUBB4Q*	-31	promoter	NA	0.184	*FRG1*	NA	NA	NA	FALSE		
chr9:137675433	2,54E-05	9,78E-02	-0.136	*MIR3689C*	65783	intron	NA	-0.052	*FCN1*	NA	NA	NA	FALSE		
chr17:5026771	2,61E-05	9,83E-02	0.134	*ZNF232*	-375	promoter	0.245	0.296	*RABEP1*	NA	NA	NA	FALSE		
chr22:49448436	2,59E-05	9,83E-02	0.267	*LOC100128946*	185856	intergenic	NA	NA	*NA*	NA	NA	NA	FALSE		
chr4:56023764	2,67E-05	9,91E-02	0.152	*KDR*	-32003	intergenic	NA	-0.086	*TMEM165*	NA	NA	NA	FALSE		
chr7:154683942	2,70E-05	9,96E-02	-0.124	*LOC100132707*	-36285	intron	NA	-0.151	*PAXIP1-AS1*	NA	NA	NA	FALSE		
chr4:81128426	2,73E-05	9,97E-02	-0.11	*PRDM8*	9771	intergenic	0.152	0.152	*PRDM8*	NA	NA	NA	FALSE		

Standalone differentially methylated CpGs, which are not part of differentially methylated regions, are marked with red color.

ESM Table 5. Differentially methylated CpGs identified in the $CD4^{-}CD8^{-}$ cell fraction between cases and controls in all longitudinal samples

Methylation difference				Nearest gene			Methylation–expression correlation analysis			eQTM analysis				GeneHancer database	
CpG site	P value	FDR	Methylation difference	Nearest gene	Distance to nearest gene	Genomic part	Nearest gene correlation, Spearman rho	The highest observed correlation, Spearman rho	Correlating gene	CpG name	eQTM, FDR	Overall Z Score	CpG found on 450K	Genehancer	Genes possibly regulated by standalone DMCs
chr18:54333583	1,26E-17	7,62E-12	0.131	*WDR7*	14969	intron	0.259	0.259	*WDR7*	NA	NA	NA	FALSE		
chr14:57523325	5,17E-13	1,25E-07	-0.299	*EXOC5*	175766	intergenic	-0.17	-0.188	*AP5M1*	NA	NA	NA	FALSE		
chr3:194706028	1,24E-12	2,51E-07	-0.132	*XXYLT1*	136897	intergenic	-0.036	NA	*NA*	NA	NA	NA	FALSE		
chr5:173991159	7,59E-12	1,32E-06	0.143	*MSX2*	-160416	intergenic	NA	NA	*NA*	NA	NA	NA	FALSE		
chr19:38346420	1,14E-11	1,72E-06	0.115	*LOC100631378*	-585	promoter	NA	-0.19	*ZFP30*	NA	NA	NA	FALSE		
chr3:194705954	3,75E-11	5,06E-06	-0.138	*XXYLT1*	136971	intergenic	-0.012	NA	*NA*	NA	NA	NA	TRUE		
chr6:166419166	5,30E-11	6,44E-06	0.104	*LINC00473*	-17640	intergenic	NA	NA	*NA*	NA	NA	NA	FALSE		
chr11:134709660	2,73E-10	2,55E-05	0.215	*AK125040*	103823	intergenic	NA	NA	*NA*	NA	NA	NA	FALSE		
chr1:161582872	7,83E-09	5,94E-04	0.12	*TRNA_Glu*	366	promoter	NA	-0.487	*FCGR2C*	NA	NA	NA	FALSE		
chr15:20374137	9,67E-09	6,90E-04	-0.122	*BC107108*	11451	intergenic	NA	NA	*NA*	NA	NA	NA	FALSE		
chr4:1522045	3,83E-08	1,94E-03	0.182	*AX748388*	55922	intergenic	NA	0.218	*TMEM129*	NA	NA	NA	FALSE		
chr22:49448575	4,97E-08	2,30E-03	0.199	*LOC100128946*	185995	intergenic	NA	NA	*NA*	NA	NA	NA	FALSE		
chr19:38346382	1,17E-07	3,73E-03	0.115	*LOC100631378*	-547	promoter	NA	-0.188	*ZFP30*	NA	NA	NA	FALSE		
chr6:2940740	1,16E-07	3,73E-03	-0.107	*SERPINB6*	17664	intergenic	0.148	0.33	*HTATSF1P2*	NA	NA	NA	FALSE		
chr19:46774424	1,28E-07	3,89E-03	0.149	*HIF3A*	-25879	intron	NA	-0.14	*PPP5C*	NA	NA	NA	FALSE	enhancer	*HIF3A*
chr2:113192477	1,45E-07	4,18E-03	0.129	*RGPD8*	-416	promoter	-0.041	0.114	*TTL*	NA	NA	NA	FALSE		
chr4:8890133	1,45E-07	4,18E-03	0.141	*HMX1*	-16591	intergenic	NA	NA	*NA*	NA	NA	NA	FALSE		
chr14:96362128	2,13E-07	5,88E-03	-0.181	*LINC00617*	19021	intron	NA	0.059	*TCL1A*	NA	NA	NA	FALSE		
chr16:32289963	2,29E-07	6,19E-03	-0.137	*LOC390705*	11340	intergenic	NA	NA	*NA*	NA	NA	NA	FALSE		
chr16:8960523	3,71E-07	8,34E-03	-0.101	*CARHSP1*	763	promoter	0.026	0.277	*ABAT*	NA	NA	NA	FALSE		
chr3:193678373	3,63E-07	8,34E-03	-0.152	*DPPA2P3*	33655	intron	NA	0.156	*HES1*	NA	NA	NA	FALSE		
chr5:167285675	3,42E-07	8,34E-03	-0.151	*TENM2*	103684	intron	NA	NA	*NA*	NA	NA	NA	FALSE		
chr7:64540916	3,69E-07	8,34E-03	-0.148	*BC044608*	723	promoter	NA	0.276	*ERV3-1*	NA	NA	NA	FALSE		
chr19:54677584	5,12E-07	1,04E-02	0.141	*TMC4*	-641	promoter	NA	0.342	*VSTM1*	NA	NA	NA	FALSE		
chr9:136075222	5,51E-07	1,08E-02	0.138	*OBP2B*	9407	intergenic	NA	0.238	*REXO4*	NA	NA	NA	FALSE		
chr9:136075028	6,02E-07	1,11E-02	0.112	*OBP2B*	9601	intergenic	NA	0.229	*SURF4*	NA	NA	NA	FALSE		
chr14:19888831	6,33E-07	1,13E-02	-0.109	*LINC00516*	-5538	intron	NA	NA	*NA*	NA	NA	NA	FALSE		
chr9:136063981	6,82E-07	1,18E-02	-0.165	*OBP2B*	20648	intergenic	NA	-0.153	*RPL7A*	NA	NA	NA	FALSE		
chr9:136075435	7,58E-07	1,30E-02	0.127	*OBP2B*	9194	intergenic	NA	0.215	*GBGT1*	NA	NA	NA	FALSE		
chr1:2370993	7,83E-07	1,31E-02	0.106	*PEX10*	-26984	intergenic	0.09	0.163	*PANK4*	NA	NA	NA	FALSE		
chr2:205393977	9,20E-07	1,49E-02	-0.102	*PARD3B*	-16539	intergenic	NA	NA	*NA*	NA	NA	NA	FALSE		
chr1:43425490	9,57E-07	1,53E-02	0.14	*SLC2A1*	-644	promoter	-0.148	0.195	*EBNA1BP2*	NA	NA	NA	FALSE		
chr5:177921624	1,06E-06	1,63E-02	-0.356	*COL23A1*	95933	intron	NA	0.227	*RN7SKP70*	NA	NA	NA	FALSE		
chr5:1004150	1,15E-06	1,74E-02	0.104	*NKD2*	-4927	intergenic	NA	-0.068	*BRD9*	NA	NA	NA	FALSE		
chr10:71801791	1,30E-06	1,88E-02	-0.131	*H2AFY2*	-10566	intergenic	NA	-0.282	*TYSND1*	NA	NA	NA	FALSE		
chr9:136075476	1,41E-06	1,99E-02	0.142	*OBP2B*	9153	intergenic	NA	0.274	*SURF1*	NA	NA	NA	FALSE		
chr4:961576	2,01E-06	2,65E-02	0.164	*DGKQ*	5773	exon	0.654	0.654	*DGKQ*	NA	NA	NA	FALSE		
chr11:187909	2,42E-06	2,99E-02	0.122	*SCGB1C1*	-5171	intergenic	NA	0.412	*RP11-326C3.2*	NA	NA	NA	FALSE		
chr19:35758166	2,57E-06	3,16E-02	0.108	*USF2*	-1730	exon	-0.04	0.207	*FXYD5*	NA	NA	NA	FALSE	promoter	*LSR*
chr19:19976235	2,71E-06	3,29E-02	0.138	*ZNF253*	-479	promoter	0.056	0.244	*ATP13A1*	NA	NA	NA	FALSE		
chr2:107200953	3,13E-06	3,52E-02	-0.205	*RGPD3*	-116153	intergenic	NA	NA	*NA*	NA	NA	NA	FALSE		
chr16:58534501	3,33E-06	3,61E-02	0.13	*NDRG4*	456	promoter	NA	-0.311	*SETD6*	NA	NA	NA	FALSE	promoter	*NDRG4*
chr17:63267120	3,30E-06	3,61E-02	-0.121	*RGS9*	82300	intergenic	-0.316	-0.316	*RGS9*	NA	NA	NA	FALSE		
chr19:519384	3,25E-06	3,61E-02	-0.148	*Mir_324*	11504	exon	NA	0.166	*BSG*	NA	NA	NA	FALSE		
chr7:155327253	3,59E-06	3,68E-02	-0.103	*CNPY1*	-715	promoter	NA	0.129	*RBM33*	NA	NA	NA	FALSE		
chr7:100304538	4,05E-06	4,06E-02	-0.131	*POP7*	86	promoter	-0.044	0.138	*SLC12A9*	NA	NA	NA	FALSE		
chr13:106062717	4,11E-06	4,09E-02	-0.153	*DAOA*	-55499	intergenic	NA	NA	*NA*	NA	NA	NA	FALSE		

chr1:151870743	4,39E-06	4,23E-02	-0.106	*THEM4*	11619	intron	-0.383	-0.383	*THEM4*	NA	NA	NA	FALSE		
chr6:52172154	5,00E-06	4,67E-02	0.219	*MCM3*	-22476	intergenic	-0.053	0.215	*EFHC1*	NA	NA	NA	FALSE		
chr18:8526512	5,45E-06	4,86E-02	-0.106	*Metazoa_SRP*	54685	intergenic	NA	0.165	*RAB12*	NA	NA	NA	FALSE		
chr11:118842392	6,61E-06	5,40E-02	0.108	*FOXR1*	-25	promoter	NA	-0.32	*DDX6*	NA	NA	NA	TRUE		
chr6:52172175	6,63E-06	5,40E-02	0.187	*MCM3*	-22497	intergenic	-0.041	-0.041	*MCM3*	NA	NA	NA	FALSE		
chr4:8890312	7,54E-06	5,79E-02	0.153	*HMX1*	-16770	intergenic	NA	NA	*NA*	NA	NA	NA	FALSE		
chr10:79395281	7,82E-06	5,90E-02	0.122	*KCNMA1*	2297	intron	NA	NA	*NA*	NA	NA	NA	FALSE		
chr3:194706077	8,47E-06	6,23E-02	-0.145	*XXYLT1*	136848	intergenic	0.046	NA	*NA*	NA	NA	NA	FALSE		
chr17:72916077	8,85E-06	6,35E-02	0.152	*USH1G*	3275	exon	NA	0.366	*NT5C*	NA	NA	NA	FALSE		
chr5:172110756	8,95E-06	6,39E-02	-0.108	*NEURL1B*	42482	exon	NA	0.245	*DUSP1*	NA	NA	NA	FALSE		
chr10:13341244	9,25E-06	6,53E-02	0.142	*PHYH*	503	promoter	0.176	0.176	*PHYH*	NA	NA	NA	FALSE	promoter	*PHYH*
chr13:106062745	1,01E-05	6,81E-02	-0.141	*DAOA*	-55471	intergenic	NA	NA	*NA*	NA	NA	NA	FALSE		
chr3:194705950	1,03E-05	6,85E-02	-0.133	*XXYLT1*	136975	intergenic	0.09	NA	*NA*	NA	NA	NA	FALSE		
chr14:19888820	1,14E-05	7,07E-02	-0.103	*LINC00516*	-5549	intron	NA	NA	*NA*	NA	NA	NA	FALSE		
chr2:176121814	1,10E-05	7,07E-02	-0.148	*ATP5G3*	-75325	intergenic	0.01	0.011	*ATF2*	NA	NA	NA	FALSE		
chr4:1522024	1,13E-05	7,07E-02	0.193	*AX748388*	55943	intergenic	NA	0.298	*UVSSA*	NA	NA	NA	FALSE		
chr4:186318341	1,13E-05	7,07E-02	-0.18	*ANKRD37*	503	promoter	0.102	-0.243	*KIAA1430*	NA	NA	NA	FALSE		
chr4:961649	1,11E-05	7,07E-02	0.128	*DGKQ*	5700	intron	0.44	0.44	*DGKQ*	NA	NA	NA	FALSE		
chr1:145385338	1,21E-05	7,33E-02	0.116	*TRNA_Asn*	-391	promoter	NA	-0.297	*RNVU1-6*	NA	NA	NA	FALSE		
chr10:118885594	1,29E-05	7,33E-02	-0.171	*KIAA1598*	504	promoter	0.14	-0.198	*SLC18A2*	NA	NA	NA	FALSE		
chr10:1974873	1,24E-05	7,33E-02	-0.15	*LINC00700*	81670	intergenic	NA	NA	*NA*	NA	NA	NA	FALSE		
chr14:19889575	1,29E-05	7,33E-02	-0.166	*LINC00516*	-4794	intron	NA	NA	*NA*	NA	NA	NA	FALSE		
chr21:28215532	1,25E-05	7,33E-02	-0.139	*ADAMTS1*	2197	intron	NA	NA	*NA*	NA	NA	NA	FALSE		
chr22:49447913	1,30E-05	7,33E-02	0.143	*LOC100128946*	185333	intergenic	NA	NA	*NA*	NA	NA	NA	FALSE		
chr19:519572	1,37E-05	7,64E-02	-0.154	*Mir_324*	11692	exon	NA	0.311	*CDC34*	NA	NA	NA	FALSE		
chr11:55640124	1,42E-05	7,75E-02	-0.105	*TRIM51*	-10649	intergenic	NA	NA	*NA*	NA	NA	NA	FALSE		
chr9:70647363	1,47E-05	7,90E-02	-0.219	*AK056618*	1312	exon	NA	0.251	*CBWD5*	NA	NA	NA	FALSE		
chr13:106062692	1,52E-05	7,97E-02	-0.15	*DAOA*	-55524	intergenic	NA	NA	*NA*	NA	NA	NA	FALSE		
chr14:103227458	1,82E-05	8,61E-02	-0.154	*TRAF3*	-16358	intergenic	0.101	0.104	*RCOR1*	NA	NA	NA	FALSE		
chr5:1004130	1,82E-05	8,61E-02	0.132	*NKD2*	-4947	intergenic	NA	0.158	*SLC12A7*	NA	NA	NA	FALSE		
chr9:136075525	1,82E-05	8,61E-02	0.107	*OBP2B*	9104	intergenic	NA	-0.198	*SURF6*	NA	NA	NA	FALSE		
chr20:61642387	2,02E-05	9,18E-02	0.12	*LOC63930*	1654	intron	NA	0.333	*COL9A3*	NA	NA	NA	FALSE		
chr2:73496203	2,04E-05	9,26E-02	-0.146	*FBXO41*	646	promoter	-0.096	-0.096	*FBXO41*	cg21918313	0.001179981	-4.619156	TRUE		
chr4:81119274	2,12E-05	9,47E-02	-0.133	*PRDM8*	619	promoter	0.219	0.219	*PRDM8*	NA	NA	NA	FALSE		
chr6:2940599	2,24E-05	9,69E-02	-0.152	*SERPINB6*	17805	intergenic	0.11	0.226	*SERPINB1*	NA	NA	NA	FALSE		
chr9:136075242	2,26E-05	9,69E-02	0.108	*OBP2B*	9387	intergenic	NA	-0.137	*RALGDS*	NA	NA	NA	FALSE		
chr14:19889577	2,39E-05	9,88E-02	-0.198	*LINC00516*	-4792	intron	NA	NA	*NA*	NA	NA	NA	FALSE		
chr4:961573	2,37E-05	9,88E-02	0.119	*DGKQ*	5776	exon	0.528	0.528	*DGKQ*	NA	NA	NA	FALSE		
chr2:73496148	2,44E-05	9,95E-02	-0.146	*FBXO41*	701	promoter	-0.136	0.342	*SMYD5*	NA	NA	NA	FALSE		
chr5:172110762	2,45E-05	9,95E-02	-0.104	*NEURL1B*	42488	exon	NA	0.256	*RP11-779O18.1*	NA	NA	NA	FALSE		

Standalone differentially methylated CpGs, which are not part of differentially methylated regions, are marked with red color.

ESM Table 6. Differentially methylated regions identified in the CD4$^+$ T cell fraction between cases and controls in all longitudinal samples

Differentially methylated region							Nearest gene			GeneHancer analysis	
DMR	Area size, bp	Number of significant CpGs	Number of positive CpGs	Number of negative CpGs	Best P value within DMR	Best FDR within FDR	Nearest gene	Distance to nearest gene	Genomic part	GeneHancer database	Genes possibly regulated by enhacer or promoter
chr2:113192161-chr2:113192585	424	6	6	0	8,45E-13	1,25E-07	*RGPD8*	-100	promoter		
chr7:22860953-chr7:22861119	166	13	0	13	2,61E-12	1,93E-07	*TOMM7*	1487	intron	promoter	*TOMM7*
chr11:118842572-chr11:118842764	192	3	3	0	1,25E-10	3,71E-06	*FOXR1*	349	promoter		
chr14:103227394-chr14:103227458	64	2	0	2	5,86E-10	1,24E-05	*TRAF3*	-16422	intergenic		
chr6:121069653-chr6:121069703	50	4	0	4	6,88E-10	1,34E-05	*C6orf170*	488506	intergenic		
chr9:137673743-chr9:137674085	342	15	0	15	7,25E-10	1,34E-05	*MIR3689C*	67367	intron		
chr19:18118304-chr19:18118337	33	2	0	2	1,11E-09	1,94E-05	*ARRDC2*	-673	promoter		
chr22:39633525-chr22:39633541	16	2	2	0	1,77E-09	2,61E-05	*PDGFB*	3390	intron	promoter	*SYNGR1, PDGFB*
chr20:47013292-chr20:47013415	123	8	0	8	8,16E-09	7,79E-05	*LINC00494*	18945	intergenic		
chr19:1336246-chr19:1336307	61	3	3	0	1,94E-08	1,59E-04	*MUM1*	-18675	intergenic		
chr17:72916000-chr17:72916113	113	13	13	0	3,45E-08	2,55E-04	*USH1G*	3275	exon		
chr6:142623602-chr6:142623625	23	2	0	2	6,26E-08	4,52E-04	*GPR126*	571	promoter		
chr18:77397978-chr18:77398380	402	6	6	0	2,46E-07	1,26E-03	*CTDP1*	-41442	intergenic		
chr9:46355235-chr9:46355291	56	2	0	2	2,74E-07	1,34E-03	*Y_RNA*	-7370	intergenic		
chr2:27038313-chr2:27038448	135	8	0	8	2,75E-07	1,34E-03	*CENPA*	29449	intergenic		
chr21:45565981-chr21:45566008	27	3	3	0	5,53E-07	2,34E-03	*C21orf33*	12503	intergenic	enhancer	*TRAPPC10, PWP2*
chr7:154585741-chr7:154585853	112	3	0	3	7,44E-07	2,82E-03	*LOC100132707*	-134486	intron		
chr1:43814462-chr1:43814672	210	15	0	15	7,64E-07	2,86E-03	*CDC20*	-9994	exon		
chr16:88453817-chr16:88453894	77	5	5	0	7,87E-07	2,91E-03	*ZNF469*	-39985	intergenic	enhancer	*ZNF469*
chr6:163570179-chr6:163570677	498	19	0	19	1,15E-06	3,87E-03	*AK296276*	42399	intron		
chr7:155832817-chr7:155832938	121	2	0	2	1,18E-06	3,92E-03	*Mir_598*	-106144	intergenic		
chr21:46714693-chr21:46714810	117	6	0	6	1,25E-06	4,07E-03	*LOC642852*	6759	exon	enhancer	*LINC00205*
chr16:2848427-chr16:2848681	254	11	11	0	1,25E-06	4,07E-03	*PRSS41*	111	promoter		
chr8:49309188-chr8:49309214	26	2	2	0	2,44E-06	6,40E-03	*EFCAB1*	338657	intergenic	enhancer	*LOC101929268*
chr7:64540896-chr7:64540960	64	3	0	3	3,23E-06	7,30E-03	*BC044608*	679	promoter	enhancer	*GTF2IP14*
chr18:60278521-chr18:60278915	394	10	10	0	3,46E-06	7,58E-03	*DKFZp451A185*	29700	intergenic		
chr21:47307634-chr21:47308422	788	13	0	13	3,80E-06	7,98E-03	*PCBP3*	-8310	intron	enhancer	*PCBP3*
chr19:48000357-chr19:48000450	93	3	0	3	3,89E-06	7,99E-03	*NAPA-AS1*	12820	intron		
chr4:186318341-chr4:186318348	7	2	0	2	4,31E-06	8,41E-03	*ANKRD37*	510	promoter	promoter	*LRP2BP, UFSP2, C4orf47*
chr4:81128344-chr4:81128539	195	5	0	5	4,71E-06	8,92E-03	*PRDM8*	9743	intergenic		
chr4:6107559-chr4:6107719	160	6	0	6	5,62E-06	1,00E-02	*JAKMIP1*	88637	intron		
chr1:228658815-chr1:228659210	395	8	8	0	6,42E-06	1,06E-02	*Histone3*	-6925	intergenic		
chr11:75993457-chr11:75993521	64	2	2	0	1,15E-05	1,48E-02	*TRNA_Pro*	-46518	intergenic		
chr12:9892250-chr12:9892305	55	2	0	2	1,15E-05	1,48E-02	*CLECL1*	-6411	intergenic	promoter	*CLEC7A, CLECL1, KLRF1*
chr8:144808903-chr8:144809619	716	8	8	0	1,24E-05	1,55E-02	*FAM83H*	3452	exon		
chr5:131607372-chr5:131607629	257	16	16	0	1,28E-05	1,58E-02	*PDLIM4*	14262	intron		
chr19:46915417-chr19:46915595	178	14	14	0	1,55E-05	1,74E-02	*CCDC8*	1369	exon	promoter	*CCDC8*
chr19:58571181-chr19:58571388	207	4	4	0	1,75E-05	1,83E-02	*ZNF135*	576	promoter	promoter	*ZNF135*
chr3:96495764-chr3:96495854	90	6	0	6	1,86E-05	1,87E-02	*EPHA6*	-37653	intergenic		
chr10:79395265-chr10:79395468	203	2	2	0	1,90E-05	1,88E-02	*KCNMA1*	2313	intron		
chr11:67351944-chr11:67351952	8	2	0	2	1,97E-05	1,90E-02	*GSTP1*	880	promoter	promoter	*NDUFV1, NUDT8, ENSG00000255119*
chr4:961400-chr4:962384	984	27	27	0	2,45E-05	2,18E-02	*DGKQ*	5944	exon		
chr3:118892305-chr3:118892496	191	3	3	0	2,85E-05	2,47E-02	*UPK1B*	-120	promoter		

chr22:31032485-chr22:31032517	32	2	2	0	2,98E-05	2,53E-02	*SLC35E4*	694	promoter	promoter	*SLC35E4*
chr3:196694100-chr3:196694374	274	8	0	8	2,99E-05	2,53E-02	*PIGZ*	1341	intron		
chr8:60032900-chr8:60032942	42	2	2	0	3,20E-05	2,63E-02	*TOX*	-1176	intergenic	promoter	*TOX*
chr14:19888706-chr14:19889020	314	2	0	2	3,37E-05	2,68E-02	*LINC00516*	-5663	intron		
chr19:37807567-chr19:37807945	378	19	19	0	3,74E-05	2,79E-02	*HKR1*	-907	promoter	promoter	*ZNF527, ZNF793, LINC01535, HKR1, ENSG00000267682*
chr20:62570508-chr20:62570635	127	8	8	0	4,02E-05	2,92E-02	*MIR1914*	2344	intergenic	enhancer	*UCKL1*
chr4:132897000-chr4:132897079	79	2	0	2	4,15E-05	3,00E-02	*BC131768*	247749	intergenic		
chr6:8436218-chr6:8436273	55	3	3	0	4,79E-05	3,30E-02	*LOC100506207*	409	promoter	promoter	*SLC35B3*
chr15:98195837-chr15:98195904	67	6	6	0	5,62E-05	3,68E-02	*BC024169*	-114410	intergenic		
chr22:26323697-chr22:26323710	13	3	0	3	5,99E-05	3,79E-02	*MYO18B*	-27435	intron		
chr17:135245-chr17:135302	57	4	4	0	6,17E-05	3,85E-02	*RPH3AL*	42126	intron		
chr12:92029135-chr12:92029279	144	5	0	5	6,33E-05	3,91E-02	*DCN*	-452362	intergenic		
chr4:160303481-chr4:160303611	130	8	8	0	7,08E-05	4,17E-02	*RAPGEF2*	114485	intergenic		
chr14:101194894-chr14:101195081	187	5	5	0	7,23E-05	4,18E-02	*DLK1*	1694	intron	promoter	*DLK1*
chr22:49447601-chr22:49448575	974	22	22	0	7,62E-05	4,30E-02	*LOC100128946*	185265	intergenic		
chr8:42052604-chr8:42052870	266	7	0	7	8,74E-05	4,71E-02	*PLAT*	-2081	intron		
chr1:178456064-chr1:178456108	44	4	4	0	8,93E-05	4,76E-02	*TEX35*	-26134	intergenic		
chr21:46677404-chr21:46677632	228	9	9	0	9,13E-05	4,79E-02	*POFUT2*	19846	intron		
chr12:32626634-chr12:32626743	109	3	0	3	9,79E-05	4,94E-02	*FGD4*	-12272	intergenic		
chr16:89150862-chr16:89151029	167	3	0	3	9,85E-05	4,94E-02	*ACSF3*	-9355	intergenic		
chr13:20711574-chr13:20711584	10	2	2	0	0,00010818	5,18E-02	*GJA3*	5854	intergenic		
chr16:89050538-chr16:89050592	54	5	5	0	0,00011464	5,34E-02	*CBFA2T3*	-7043	intergenic	enhancer	*CBFA2T3*
chr16:32289963-chr16:32290350	387	3	0	3	0,0001208	5,46E-02	*LOC390705*	11238	intergenic		
chr12:133414368-chr12:133414497	129	9	9	0	0,00012569	5,56E-02	*GOLGA3*	-9001	intergenic	enhancer	*CHFR*
chr7:76129266-chr7:76129434	168	8	0	8	0,00012915	5,64E-02	*DTX2*	156	promoter		
chr9:136075242-chr9:136075539	297	8	8	0	0,00014671	6,01E-02	*OBP2B*	9194	intergenic		
chr4:8890133-chr4:8891755	1622	3	3	0	0,00018867	7,06E-02	*HMX1*	-16591	intergenic		
chr22:49442398-chr22:49442415	17	2	0	2	0,00020347	7,40E-02	*LOC100128946*	179835	intergenic		
chr19:1466099-chr19:1466162	63	8	8	0	0,00021329	7,59E-02	*C19orf25*	13130	exon	enhancer	*C19orf25*
chr5:3605658-chr5:3606057	399	3	3	0	0,00021449	7,61E-02	*IRX1*	9561	intergenic		
chr1:149287263-chr1:149287303	40	2	0	2	0,00022958	7,91E-02	*BC023516*	-188	promoter		
chr3:194705981-chr3:194706091	110	2	0	2	0,0002553	8,33E-02	*XXYLT1*	136834	intergenic		
chr7:158111329-chr7:158111331	2	2	2	0	0,00025746	8,36E-02	*MIR595*	214177	intron		
chr22:17198946-chr22:17199120	174	8	0	8	0,00027805	8,69E-02	*BC038197*	30355	intergenic		
chr22:44529983-chr22:44530101	118	5	5	0	0,00029345	8,95E-02	*TRNA_SeC*	-16509	intron		
chr8:144787916-chr8:144788674	758	9	9	0	0,00033152	9,52E-02	*CCDC166*	1638	intron		

ESM Table 7. Differentially methylated regions identified in the CD8$^+$ T cell fraction between cases and controls in all longitudinal samples

Differentially methylated region							Nearest gene			GeneHancer analysis	
DMR	Area size, bp	Number of significant CpGs	Number of positive CpGs	Number of negative CpGs	Best P value within DMR	Best FDR within FDR	Nearest gene	Distance to nearest gene	Genomic part	GeneHancer database	Genes possibly regulated by enhancer or promoter
chr16:32289928-chr16:32290543	615	12	0	12	5,21E-11	1,27E-05	*LOC390705*	11375	intergenic		
chr2:27038313-chr2:27038482	169	11	1	10	2,78E-10	4,31E-05	*CENPA*	29459	intergenic		
chr14:45343058-chr14:45343116	58	2	0	2	8,55E-10	9,11E-05	*C14orf28*	-23449	intergenic		
chr16:433855-chr16:434103	248	4	0	4	1,14E-09	1,08E-04	*LOC100134368*	1688	intron	Promoter	*TMEM8A, MRPL28*
chr22:39633525-chr22:39633541	16	2	2	0	1,14E-09	1,08E-04	*PDGFB*	3390	intron	Promoter	*CBX7, SYNGR1, PDGFB*
chr7:3169658-chr7:3169674	16	2	2	0	3,27E-09	2,65E-04	*BC038729*	44630	intergenic		
chr16:58534501-chr16:58534640	139	2	2	0	6,77E-09	4,44E-04	*NDRG4*	456	promoter	Promoter	*NDRG4*
chr1:145385266-chr1:145385422	156	15	14	1	1,02E-08	5,82E-04	*TRNA_Asn*	-430	promoter		
chr16:8960454-chr16:8960833	379	10	0	10	1,59E-08	8,76E-04	*CARHSP1*	772	promoter	Promoter	*PMM2, CARHSP1, ENSG00000260276*
chr14:101194267-chr14:101195081	814	7	7	0	1,69E-08	8,85E-04	*DLK1*	1694	intron	Promoter	*DLK1*
chr3:196693993-chr3:196694364	371	18	0	18	1,81E-08	8,85E-04	*PIGZ*	1341	intron		
chr8:144788607-chr8:144788806	199	7	7	0	3,95E-08	1,38E-03	*CCDC166*	1638	intron		
chr12:116978050-chr12:116978111	61	2	0	2	1,43E-07	3,47E-03	*LINC00173*	6812	intergenic	Enhancer	*MED13L, LINC00173*
chr16:3116115-chr16:3116616	501	11	0	11	1,68E-07	3,82E-03	*IL32*	580	promoter	Promoter	*IL32*
chr5:3605658-chr5:3605727	69	4	4	0	2,16E-07	4,67E-03	*IRX1*	9546	intergenic		
chr18:844055-chr18:844167	112	2	0	2	3,13E-07	6,14E-03	*YES1*	-31626	intergenic		
chr7:2060040-chr7:2060130	90	2	2	0	4,39E-07	7,92E-03	*MAD1L1*	-79856	intron		
chr10:1416791-chr10:1416983	192	17	0	17	6,03E-07	1,00E-02	*ADARB2-AS1*	-151997	intron		
chr6:158630758-chr6:158631170	412	12	0	12	6,72E-07	1,06E-02	*GTF2H5*	41392	intergenic		
chr20:2690382-chr20:2690396	14	2	0	2	8,53E-07	1,26E-02	*EBF4*	16860	intron		
chr22:49447601-chr22:49448575	974	26	26	0	8,76E-07	1,28E-02	*LOC100128946*	185265	intergenic		
chr10:118885541-chr10:118885760	219	11	0	11	9,29E-07	1,34E-02	*KIAA1598*	459	promoter		
chr11:118842603-chr11:118842764	161	2	2	0	9,67E-07	1,37E-02	*FOXR1*	349	promoter		
chr2:113192114-chr2:113192585	471	9	9	0	1,08E-06	1,50E-02	*RGPD8*	-524	promoter		
chr17:80057499-chr17:80057639	140	6	6	0	1,39E-06	1,73E-02	*FASN*	-1526	intergenic	Promoter	*HGS, DCXR, GPS1, FOXK2, HEXDC, CCDC57, FASN*
chr4:123286278-chr4:123286282	4	2	0	2	1,54E-06	1,87E-02	*ADAD1*	-13843	intergenic		
chr7:2903150-chr7:2903323	173	4	4	0	1,63E-06	1,95E-02	*GNA12*	-19192	intergenic	Enhancer	*GNA12*
chr5:178156527-chr5:178156747	220	7	0	7	1,91E-06	2,10E-02	*ZNF354A*	1052	intron	Promoter	*ZNF354A*
chr9:70937429-chr9:70937516	87	4	0	4	2,14E-06	2,24E-02	*FOXD4L3*	19677	intergenic		
chr4:184602661-chr4:184602729	68	2	2	0	2,56E-06	2,56E-02	*TRAPPC11*	-2341	intron		
chr3:96495758-chr3:96495824	66	6	0	6	3,56E-06	3,13E-02	*EPHA6*	-37661	intergenic		
chr2:2923575-chr2:2923622	47	2	0	2	3,74E-06	3,23E-02	*AK095310*	206177	intron		
chr13:19183634-chr13:19183707	73	12	0	12	3,91E-06	3,34E-02	*LINC00417*	130533	intergenic		
chr19:1466022-chr19:1466187	165	5	5	0	5,02E-06	3,94E-02	*C19orf25*	13112	exon	Enhancer	*C19orf25*
chr10:135092506-chr10:135092612	106	5	5	0	5,12E-06	3,96E-02	*ADAM8*	-2100	exon	Promoter	*TUBGCP2, ADAM8*
chr17:80272367-chr17:80272875	508	2	2	0	6,23E-06	4,44E-02	*CD7*	2606	exon		
chr21:10989990-chr21:10990928	938	4	0	4	7,39E-06	4,96E-02	*TPTE*	439	promoter		
chr8:11812867-chr8:11813054	187	2	2	0	7,81E-06	5,16E-02	*DEFB136*	19242	intergenic		
chr19:37807633-chr19:37807945	312	20	20	0	8,56E-06	5,49E-02	*HKR1*	-993	promoter	promoter	*ZNF527, LINC01535, ZNF793, HKR1, ENSG00000267682*
chr18:40176479-chr18:40176497	18	3	0	3	9,80E-06	5,93E-02	*Mir_544*	-203586	intron		
chr5:131822690-chr5:131822736	46	2	2	0	1,03E-05	6,07E-02	*IRF1*	2440	exon	promoter	*RAD50, IRF1, AFF4, LOC105379175*
chr3:118892305-chr3:118892482	177	8	8	0	1,04E-05	6,08E-02	*UPK1B*	-120	promoter		
chr7:331214-chr7:331219	5	2	2	0	1,25E-05	6,70E-02	*LOC100288524*	1080	exon		
chr4:1521984-chr4:1522395	411	21	21	0	1,32E-05	6,77E-02	*AX748388*	55928	intergenic		
chr6:158636025-chr6:158636092	67	2	0	2	1,33E-05	6,77E-02	*GTF2H5*	46715	intergenic		

chr18:74118107-chr18:74118303	196	8	0	8	1,43E-05	6,95E-02	*ZNF516*	-25939	intron		
chr15:27139216-chr15:27139242	26	2	2	0	1,53E-05	7,22E-02	*GABRA5*	26945	intron		
chr16:21200414-chr16:21200547	133	10	10	0	1,67E-05	7,58E-02	*ZP2*	22630	intergenic	Enhancer	*TMEM159*
chr7:52341601-chr7:52341729	128	11	0	11	1,85E-05	8,07E-02	*POM121L12*	-761748	intergenic		
chr19:575488-chr19:575524	36	2	0	2	1,92E-05	8,31E-02	*BSG*	3072	intron	Promoter	*LOC105372233, BSG*
chr4:190906006-chr4:190906084	78	4	0	4	2,46E-05	9,67E-02	*TUBB4Q*	-31	promoter		
chr9:137673743-chr9:137675473	1730	23	0	23	2,54E-05	9,78E-02	*MIR3689C*	67218	intron		
chr17:5026743-chr17:5026771	28	2	2	0	2,61E-05	9,83E-02	*ZNF232*	-375	promoter		
chr4:56023608-chr4:56023764	156	4	4	0	2,67E-05	9,91E-02	*KDR*	-31990	intergenic		
chr7:154683942-chr7:154684327	385	2	0	2	2,70E-05	9,96E-02	*LOC100132707*	-35900	exon		
chr4:81128364-chr4:81128544	180	6	0	6	2,73E-05	9,97E-02	*PRDM8*	9884	intergenic		

ESM Table 8. Differentially methylated regions identified in the $CD4^{-}CD8^{-}$ cell fraction between cases and controls in all longitudinal samples

Differentially methylated region							Nearest gene			GeneHancer analysis	
DMR	Area size, bp	Number of significant CpGs	Number of positive CpGs	Number of negative CpGs	Best P value within DMR	Best FDR within FDR	Nearest gene	Distance to nearest gene	Genomic part	GeneHancer database	Genes possibly regulated by enhacer or promoter
chr3:194705950-chr3:194706097	147	9	0	9	1,24E-12	2,51E-07	*XXYLT1*	136848	intergenic		
chr19:38346382-chr19:38346420	38	2	2	0	1,14E-11	1,72E-06	*LOC100631378*	-547	promoter		
chr6:166419049-chr6:166419166	117	3	3	0	5,30E-11	6,44E-06	*LINC00473*	-17530	intergenic		
chr4:1521934-chr4:1522045	111	7	7	0	3,83E-08	1,94E-03	*AX748388*	55943	intergenic		
chr22:49447601-chr22:49448582	981	20	20	0	4,97E-08	2,30E-03	*LOC100128946*	185265	intergenic		
chr6:2940599-chr6:2940746	147	8	0	8	1,16E-07	3,73E-03	*SERPINB6*	17805	intergenic	enhancer	*SERPINB9, SERPINB6*
chr4:8890133-chr4:8891755	1622	5	5	0	1,45E-07	4,18E-03	*HMX1*	-16770	intergenic		
chr2:113192477-chr2:113192585	108	2	2	0	1,45E-07	4,18E-03	*RGPD8*	-416	promoter		
chr14:96362128-chr14:96362157	29	2	0	2	2,13E-07	5,88E-03	*LINC00617*	19021	intron		
chr16:32289963-chr16:32290281	318	8	0	8	2,29E-07	6,19E-03	*LOC390705*	11107	intergenic		
chr5:167285661-chr5:167285753	92	14	0	14	3,42E-07	8,34E-03	*TENM2*	103679	intron		
chr3:193678336-chr3:193678373	37	2	0	2	3,63E-07	8,34E-03	*DPPA2P3*	33655	intron		
chr7:64540916-chr7:64540960	44	3	0	3	3,69E-07	8,34E-03	*BC044608*	723	promoter	enhancer	*GTF2IP14*
chr16:8960514-chr16:8960728	214	6	0	6	3,71E-07	8,34E-03	*CARHSP1*	587	promoter		
chr9:136074985-chr9:136075525	540	15	15	0	5,51E-07	1,08E-02	*OBP2B*	9153	intergenic		
chr14:19888706-chr14:19889624	918	13	0	13	6,33E-07	1,13E-02	*LINC00516*	-4792	intron		
chr9:136063893-chr9:136063981	88	2	0	2	6,82E-07	1,18E-02	*OBP2B*	20648	intergenic		
chr1:43425385-chr1:43425514	129	7	7	0	9,57E-07	1,53E-02	*SLC2A1*	-644	promoter		*SVBP, SLC2A1; SLC2A1-AS1*
chr5:1004130-chr5:1004250	120	6	6	0	1,15E-06	1,74E-02	*NKD2*	-4827	intergenic		
chr10:71801559-chr10:71801791	232	2	0	2	1,30E-06	1,88E-02	*H2AFY2*	-10798	intergenic		
chr4:961370-chr4:961649	279	21	21	0	2,01E-06	2,65E-02	*DGKQ*	5944	exon		
chr11:187771-chr11:187909	138	3	3	0	2,42E-06	2,99E-02	*SCGB1C1*	-5309	intergenic	enhancer	*SCGB1C1, BET1L*
chr19:19976235-chr19:19976265	30	3	3	0	2,71E-06	3,29E-02	*ZNF253*	-479	promoter		
chr2:107200907-chr2:107200963	56	9	0	9	3,13E-06	3,52E-02	*RGPD3*	-116153	intergenic		
chr19:519384-chr19:519572	188	2	0	2	3,25E-06	3,61E-02	*Mir_324*	11692	exon	enhancer	*TPGS1*
chr7:155327253-chr7:155327298	45	2	0	2	3,59E-06	3,68E-02	*CNPY1*	-760	promoter		
chr13:106062680-chr13:106062745	65	6	0	6	4,11E-06	4,09E-02	*DAOA*	-55499	intergenic		
chr1:151870613-chr1:151870743	130	4	0	4	4,39E-06	4,23E-02	*THEM4*	11749	intron	enhancer	*THEM4*
chr6:52172154-chr6:52172175	21	2	2	0	5,00E-06	4,67E-02	*MCM3*	-22476	intergenic		
chr18:8526512-chr18:8526669	157	2	0	2	5,45E-06	4,86E-02	*Metazoa_SRP*	54842	intergenic		
chr11:118842392-chr11:118842764	372	3	3	0	6,61E-06	5,40E-02	*FOXR1*	347	promoter		
chr17:72916021-chr17:72916113	92	7	7	0	8,85E-06	6,35E-02	*USH1G*	3275	exon		
chr5:172110665-chr5:172110770	105	7	0	7	8,95E-06	6,39E-02	*NEURL1B*	42391	exon	enhancer	*NEURL1B*
chr2:176121784-chr2:176121927	143	6	0	6	1,10E-05	7,07E-02	*ATP5G3*	-75360	intergenic		
chr4:186318341-chr4:186318356	15	3	0	3	1,13E-05	7,07E-02	*ANKRD37*	503	promoter		
chr1:145385315-chr1:145385536	221	4	4	0	1,21E-05	7,33E-02	*TRNA_Asn*	-414	promoter		
chr10:1974873-chr10:1975086	213	5	0	5	1,24E-05	7,33E-02	*LINC00700*	81670	intergenic		
chr21:28215532-chr21:28215691	159	3	0	3	1,25E-05	7,33E-02	*ADAMTS1*	2197	intron		

chr10:118885541-chr10:118885787	246	16	0	16	1,29E-05	7,33E-02	*KIAA1598*	468	promoter		
chr11:55640097-chr11:55640479	382	15	0	15	1,42E-05	7,75E-02	*TRIM51*	-10401	intergenic		
chr9:70647209-chr9:70647363	154	11	0	11	1,47E-05	7,90E-02	*AK056618*	1220	exon		
chr14:103227394-chr14:103227569	175	7	0	7	1,82E-05	8,61E-02	*TRAF3*	-16422	intergenic		
chr20:61642269-chr20:61642401	132	9	9	0	2,02E-05	9,18E-02	*LOC63930*	1626	intron	enhancer	*BHLHE23; LINC01749*
chr2:73496132-chr2:73496413	281	21	0	21	2,04E-05	9,26E-02	*FBXO41*	670	promoter		
chr4:81118267-chr4:81119481	1214	14	0	14	2,12E-05	9,47E-02	*PRDM8*	826	promoter		

ESM Table 9. Differentially methylated CpGs identified in the $CD4^+$ T cell fraction between cases and controls prior to seroconversion

Methylation difference				Nearest gene			Methylation-expression correlation analysis			eQTM analysis			
CpG site	P value	FDR	Methylation difference	Nearest gene	Distance to nearest gene	Genomic part	Nearest gene correlation, Spearman rho	The highest observed correlation, Spearman rho	Correlating gene	CpG name	eQTM, FDR	Overall Z Score	CpG found on 450K
chr19:18118304	4,22E-22	1,64E-16	-0.185	*ARRDC2*	-673	promoter	0.169	-0.402	*IFI30*	NA	NA	NA	FALSE
chr7:3169658	4,76E-13	4,62E-08	0.269	*BC038729*	44630	intergenic	NA	0.216	*CARD11*	NA	NA	NA	FALSE
chr10:99209697	1,95E-12	1,52E-07	0.541	*ZDHHC16*	3769	intron	0.306	0.484	*MMS19*	NA	NA	NA	FALSE
chr14:65542789	7,00E-12	3,88E-07	-0.505	*LOC100506321*	-13847	intron	NA	0.326	*RP11-840I19.*	NA	NA	NA	FALSE
chr8:530556	1,00E-11	4,87E-07	-0.171	*TDRP*	-34776	intergenic	NA	0.283	*FBXO25*	NA	NA	NA	FALSE
chr2:27038329	1,50E-11	6,25E-07	-0.226	*CENPA*	29449	intergenic	NA	0.248	*AGBL5*	NA	NA	NA	FALSE
chr19:37807898	1,61E-11	6,25E-07	0.149	*HKR1*	-915	promoter	0.057	-0.509	*ZNF527*	NA	NA	NA	FALSE
chr2:113192477	6,97E-11	2,25E-06	0.145	*RGPD8*	-416	promoter	-0.248	0.467	*TTL*	NA	NA	NA	FALSE
chr2:27038339	8,15E-11	2,43E-06	-0.23	*CENPA*	29459	intergenic	NA	0.238	*AGBL5*	NA	NA	NA	FALSE
chr4:4355602	1,11E-10	3,08E-06	0.189	*NSG1*	5735	intron	NA	0.228	*TMEM128*	NA	NA	NA	FALSE
chr2:27038313	1,44E-10	3,49E-06	-0.216	*CENPA*	29433	intergenic	NA	-0.251	*TMEM214*	NA	NA	NA	TRUE
chr4:2307965	1,52E-10	3,49E-06	0.237	*ZFYVE28*	34844	intron	-0.244	-0.244	*ZFYVE28*	NA	NA	NA	FALSE
chr2:27038396	1,81E-10	3,90E-06	-0.224	*CENPA*	29516	intergenic	NA	0.251	*AGBL5*	NA	NA	NA	FALSE
chr2:27038406	2,55E-10	4,94E-06	-0.214	*CENPA*	29526	intergenic	NA	-0.266	*TMEM214*	NA	NA	NA	FALSE
chr15:94335575	2,49E-10	4,94E-06	0.274	*BC037497*	108254	intron	NA	NA	*NA*	NA	NA	NA	FALSE
chr2:27038408	4,85E-09	8,18E-05	-0.21	*CENPA*	29528	intergenic	NA	-0.303	*TMEM214*	NA	NA	NA	FALSE
chr19:37807915	6,87E-09	1,07E-04	0.147	*HKR1*	-898	promoter	0.083	-0.489	*ZNF527*	NA	NA	NA	FALSE
chr2:27038368	7,72E-09	1,15E-04	-0.222	*CENPA*	29488	intergenic	NA	0.222	*AGBL5*	NA	NA	NA	FALSE
chr22:39633525	1,04E-08	1,49E-04	0.198	*PDGFB*	3390	intron	NA	-0.342	*APOBEC3G*	NA	NA	NA	FALSE
chr19:58571349	1,36E-08	1,75E-04	0.121	*ZNF135*	744	promoter	NA	-0.512	*ZNF417*	NA	NA	NA	FALSE
chr19:37807709	1,70E-08	2,06E-04	0.114	*HKR1*	-1104	intron	0.028	-0.445	*ZNF527*	NA	NA	NA	FALSE
chr5:270987	2,15E-08	2,53E-04	0.206	*PDCD6*	-749	promoter	0.082	0.394	*CTD-2083E4.4*	NA	NA	NA	FALSE
chr22:39633541	2,94E-08	3,25E-04	0.171	*PDGFB*	3374	intron	NA	-0.379	*APOBEC3D*	NA	NA	NA	FALSE
chr8:98812754	3,12E-08	3,27E-04	0.695	*LAPTM4B*	24947	intron	-0.182	-0.446	*MTDH*	NA	NA	NA	FALSE
chr18:77398359	3,06E-08	3,27E-04	0.18	*CTDP1*	-41442	intergenic	0.093	0.093	*CTDP1*	NA	NA	NA	FALSE
chr1:228659024	3,33E-08	3,40E-04	0.147	*Histone3*	-7134	intergenic	NA	0.136	*TRIM11*	NA	NA	NA	FALSE
chr19:37807906	3,45E-08	3,43E-04	0.224	*HKR1*	-907	promoter	0.086	-0.504	*ZNF527*	NA	NA	NA	FALSE
chr7:22860953	3,68E-08	3,56E-04	-0.154	*TOMM7*	1519	intron	0.14	-0.158	*IL6*	NA	NA	NA	FALSE
chr21:47285711	4,88E-08	4,61E-04	-0.551	*PCBP3*	15838	intron	-0.494	-0.494	*PCBP3*	NA	NA	NA	FALSE
chr16:85522260	5,07E-08	4,68E-04	-0.155	*GSE1*	-122769	intergenic	-0.05	-0.05	*GSE1*	NA	NA	NA	FALSE
chr18:77398198	6,31E-08	5,69E-04	0.255	*CTDP1*	-41603	intergenic	-0.071	-0.341	*NFATC1*	NA	NA	NA	FALSE
chr17:21294720	6,68E-08	5,89E-04	-0.116	*KCNJ12*	-13728	intron	NA	0.318	*TMEM11*	NA	NA	NA	FALSE
chr19:37807945	7,23E-08	6,23E-04	0.129	*HKR1*	-868	promoter	0.046	-0.521	*ZNF527*	NA	NA	NA	FALSE
chr21:47307815	7,69E-08	6,48E-04	-0.207	*PCBP3*	-8307	intron	-0.496	-0.496	*PCBP3*	NA	NA	NA	FALSE
chr17:55095408	1,13E-07	9,16E-04	0.283	*RNF126P1*	-27431	intergenic	NA	-0.356	*AKAP1*	NA	NA	NA	FALSE
chr14:103227394	1,19E-07	9,42E-04	-0.205	*TRAF3*	-16422	intergenic	0.4	0.4	*TRAF3*	NA	NA	NA	FALSE

chr2:130794726	1,29E-07	1,00E-03	-0.186	*LOC440905*	-5236	intron	NA	0.232	*SMPD4*	NA	NA	NA		FALSE
chr12:32626634	1,42E-07	1,08E-03	-0.387	*FGD4*	-12272	intergenic	NA	-0.239	*DNM1L*	NA	NA	NA		FALSE
chr6:13873766	1,56E-07	1,10E-03	-0.112	*RNF182*	-50911	intergenic	NA	0.195	*MCUR1*	NA	NA	NA		FALSE
chr9:136075435	1,56E-07	1,10E-03	0.178	*OBP2B*	9194	intergenic	NA	-0.393	*SURF2*	NA	NA	NA		FALSE
chr11:116506019	1,67E-07	1,14E-03	0.143	*BUD13*	137696	intergenic	-0.044	-0.23	*ZPR1*	NA	NA	NA		FALSE
chr22:47130395	1,94E-07	1,21E-03	0.149	*CERK*	3758	intron	0.136	-0.34	*TBC1D22A*	NA	NA	NA		FALSE
chr19:37807937	2,11E-07	1,30E-03	0.19	*HKR1*	-876	promoter	0.024	-0.475	*ZNF527*	NA	NA	NA		FALSE
chr22:49447830	2,27E-07	1,33E-03	0.259	*LOC100128946*	185250	intergenic	NA	NA	*NA*	NA	NA	NA		FALSE
chr7:22861070	3,74E-07	2,01E-03	-0.179	*TOMM7*	1402	intron	0.296	0.296	*TOMM7*	NA	NA	NA		FALSE
chr9:137674072	4,05E-07	2,15E-03	-0.198	*MIR3689C*	67144	intron	NA	-0.07	*FCN1*	NA	NA	NA		FALSE
chr19:2561793	4,47E-07	2,34E-03	0.273	*BC022568*	82057	intron	NA	0.345	*THOP1*	NA	NA	NA		FALSE
chr1:7908887	5,23E-07	2,48E-03	0.484	*UTS2*	4292	intron	NA	0.124	*PARK7*	NA	NA	NA		FALSE
chr10:71801791	5,16E-07	2,48E-03	-0.147	*H2AFY2*	-10566	intergenic	-0.066	0.352	*PPA1*	NA	NA	NA		FALSE
chr4:186808118	5,70E-07	2,66E-03	-0.159	*SORBS2*	69753	intron	NA	NA	*NA*	NA	NA	NA		FALSE
chr15:93277255	7,08E-07	3,19E-03	0.211	*FAM174B*	50	promoter	-0.228	-0.265	*AC013394.2*	NA	NA	NA		FALSE
chr19:12035043	7,03E-07	3,19E-03	0.105	*ZNF700*	-840	promoter	0.239	-0.342	*CTD-2006C1.2*	NA	NA	NA		FALSE
chr19:58571295	7,18E-07	3,20E-03	0.116	*ZNF135*	690	promoter	NA	-0.356	*ZNF417*	NA	NA	NA		FALSE
chr16:452426	7,79E-07	3,36E-03	-0.124	*DECR2*	535	promoter	-0.496	-0.623	*LA16c-OS12.2*	NA	NA	NA		FALSE
chr5:43000457	8,34E-07	3,52E-03	-0.108	*AK056817*	-7023	intergenic	NA	0.21	*ANXA2R*	NA	NA	NA		FALSE
chr9:137673894	1,08E-06	4,21E-03	-0.271	*MIR3689C*	67322	intron	NA	0.267	*FCN1*	NA	NA	NA		TRUE
chr13:48892804	1,09E-06	4,22E-03	-0.134	*RB1*	14923	intron	-0.086	0.259	*MED4*	NA	NA	NA		FALSE
chr16:14637963	1,25E-06	4,75E-03	-0.405	*PARN*	86166	intron	0.029	0.029	*PARN*	NA	NA	NA		FALSE
chr1:30911320	1,30E-06	4,84E-03	-0.129	*MATN1-AS1*	-280299	intergenic	0.174	NA	*NA*	NA	NA	NA		FALSE
chr4:961405	1,29E-06	4,84E-03	0.325	*DGKQ*	5944	exon	0.504	0.504	*DGKQ*	NA	NA	NA		FALSE
chr4:132897079	1,44E-06	5,18E-03	-0.127	*BC131768*	247828	intergenic	NA	NA	*NA*	NA	NA	NA		FALSE
chr3:171464841	1,62E-06	5,65E-03	-0.102	*PLD1*	63664	intron	NA	NA	*NA*	NA	NA	NA		FALSE
chr4:39569133	1,72E-06	5,84E-03	-0.371	*SMIM14*	16446	intron	NA	0.398	*RFC1*	NA	NA	NA		FALSE
chr19:1336301	1,77E-06	5,90E-03	0.183	*MUM1*	-18675	intergenic	-0.016	-0.372	*C19orf24*	NA	NA	NA		FALSE
chr10:74082076	1,84E-06	6,01E-03	0.158	*DNAJB12*	32832	intergenic	-0.078	0.268	*SPOCK2*	NA	NA	NA		FALSE
chr10:134499085	2,33E-06	7,17E-03	-0.229	*INPP5A*	77668	intron	-0.318	-0.318	*INPP5A*	NA	NA	NA		FALSE
chr19:609136	2,44E-06	7,34E-03	-0.138	*HCN2*	19245	intron	NA	-0.416	*POLRMT*	NA	NA	NA		FALSE
chr7:64540896	2,66E-06	7,76E-03	-0.184	*BC044608*	743	promoter	NA	0.408	*ZNF273*	NA	NA	NA		FALSE
chr22:44530074	2,95E-06	8,40E-03	0.161	*TRNA_SeC*	-16463	intron	NA	0.217	*SAMM50*	NA	NA	NA		FALSE
chr3:196694364	3,06E-06	8,65E-03	-0.258	*PIGZ*	1341	intron	NA	-0.381	*AC127904.2*	NA	NA	NA		FALSE
chr16:4751653	3,14E-06	8,75E-03	-0.126	*ANKS3*	-1350	intron	-0.064	0.377	*NUDT16L1*	NA	NA	NA		FALSE
chr11:122427550	3,37E-06	9,14E-03	-0.203	*TRNA_Lys*	-3105	intergenic	NA	-0.024	*UBASH3B*	NA	NA	NA		FALSE
chr17:14362402	3,81E-06	9,72E-03	-0.113	*MGC12916*	155347	intergenic	0.039	-0.186	*HS3ST3B1*	NA	NA	NA		FALSE
chr18:77398164	3,96E-06	1,00E-02	0.174	*CTDP1*	-41637	intergenic	0.109	-0.129	*NFATC1*	NA	NA	NA		FALSE
chr11:122427606	4,02E-06	1,01E-02	-0.214	*TRNA_Lys*	-3049	intergenic	NA	-0.041	*UBASH3B*	NA	NA	NA		FALSE
chr4:961427	4,86E-06	1,16E-02	0.246	*DGKQ*	5922	exon	0.434	0.434	*DGKQ*	NA	NA	NA		FALSE
chr22:23552492	4,88E-06	1,16E-02	-0.168	*BCR*	-6444	intron	-0.391	-0.391	*BCR*	NA	NA	NA		FALSE
chr2:113192407	5,26E-06	1,22E-02	0.139	*RGPD8*	-346	promoter	-0.022	0.336	*TTL*	NA	NA	NA		FALSE
chr8:95962383	5,46E-06	1,22E-02	-0.103	*TP53INP1*	-769	promoter	-0.428	-0.428	*TP53INP1*	NA	NA	NA		TRUE

chr6:28459146	5,92E-06	1,30E-02	-0.107	*TRNA_Thr*	-2304	intergenic	NA	-0.177	*ZSCAN26*	NA	NA	NA	FALSE
chr9:136075389	5,97E-06	1,31E-02	0.111	*OBP2B*	9240	intergenic	NA	0.3	*RPL7A*	NA	NA	NA	FALSE
chr9:136075393	6,07E-06	1,32E-02	0.149	*OBP2B*	9236	intergenic	NA	-0.306	*CACFD1*	NA	NA	NA	TRUE
chr13:21900452	6,16E-06	1,34E-02	-0.102	*MIPEPP3*	28190	intron	NA	0.482	*ZDHHC20*	NA	NA	NA	FALSE
chr16:89050592	6,80E-06	1,43E-02	0.247	*CBFA2T3*	-7089	intergenic	NA	0.197	*ACSF3*	NA	NA	NA	FALSE
chr20:56247315	6,78E-06	1,43E-02	-0.101	*PMEPA1*	18366	intron	0.105	-0.253	*ZBP1*	NA	NA	NA	FALSE
chr19:37807939	8,29E-06	1,65E-02	0.167	*HKR1*	-874	promoter	0.018	-0.436	*ZNF570*	NA	NA	NA	FALSE
chr19:30544746	8,44E-06	1,66E-02	0.2	*URI1*	68647	intergenic	0.116	-0.234	*CCNE1*	NA	NA	NA	FALSE
chr7:98029163	8,81E-06	1,67E-02	-0.123	*BAIAP2L1*	1265	intron	NA	0.243	*BRI3*	NA	NA	NA	FALSE
chr10:15038016	8,55E-06	1,67E-02	0.109	*DCLRE1C*	25847	intron	0.232	0.232	*DCLRE1C*	NA	NA	NA	FALSE
chr18:60278811	8,66E-06	1,67E-02	0.157	*DKFZp451A185*	29775	intergenic	NA	0.246	*ZCCHC2*	NA	NA	NA	FALSE
chr3:193693011	9,32E-06	1,68E-02	-0.144	*DPPA2P3*	19017	intron	NA	-0.014	*HES1*	NA	NA	NA	FALSE
chr15:101661806	9,37E-06	1,68E-02	-0.259	*CHSY1*	66461	intergenic	0.069	0.23	*VIMP*	NA	NA	NA	FALSE
chr15:93277269	9,62E-06	1,71E-02	0.204	*FAM174B*	36	promoter	-0.194	-0.269	*AC013394.2*	NA	NA	NA	FALSE
chr17:48586137	9,66E-06	1,71E-02	0.168	*MYCBPAP*	394	promoter	NA	-0.51	*ANKRD40*	NA	NA	NA	FALSE
chr18:77474920	1,02E-05	1,75E-02	-0.244	*CTDP1*	33492	exon	-0.418	-0.446	*PQLC1*	NA	NA	NA	FALSE
chr11:43321894	1,03E-05	1,76E-02	-0.299	*API5*	-11611	intergenic	0.135	-0.139	*TTC17*	NA	NA	NA	FALSE
chr2:113192512	1,05E-05	1,76E-02	0.142	*RGPD8*	-451	promoter	0.107	0.375	*POLR1B*	NA	NA	NA	FALSE
chr5:176874966	1,08E-05	1,80E-02	-0.195	*PRR7-AS1*	-22	promoter	-0.151	-0.475	*RGS14*	NA	NA	NA	FALSE
chr22:17199041	1,09E-05	1,80E-02	-0.116	*BC038197*	30288	intergenic	NA	0.182	*TPTEP1*	NA	NA	NA	FALSE
chr14:103227458	1,17E-05	1,87E-02	-0.159	*TRAF3*	-16358	intergenic	0.324	0.324	*TRAF3*	NA	NA	NA	FALSE
chr18:60278889	1,18E-05	1,87E-02	0.163	*DKFZp451A185*	29853	intergenic	NA	0.069	*PHLPP1*	NA	NA	NA	FALSE
chr13:48892827	1,23E-05	1,91E-02	-0.149	*RB1*	14946	intron	-0.119	0.256	*MED4*	NA	NA	NA	FALSE
chr19:18118337	1,23E-05	1,91E-02	-0.122	*ARRDC2*	-640	promoter	0.14	-0.465	*IFI30*	NA	NA	NA	FALSE
chr19:58521598	1,30E-05	1,99E-02	0.13	*ZNF606*	-6885	intergenic	-0.228	-0.372	*ZNF417*	NA	NA	NA	FALSE
chr12:133304192	1,38E-05	2,08E-02	-0.142	*ANKLE2*	7084	intron	-0.22	-0.248	*FBRSL1*	NA	NA	NA	FALSE
chr5:13939870	1,41E-05	2,11E-02	0.118	*DNAH5*	4720	intron	NA	-0.24	*TRIO*	NA	NA	NA	FALSE
chr16:2892482	1,48E-05	2,17E-02	-0.12	*PRSS30P*	-10	promoter	NA	-0.449	*AC141586.5*	NA	NA	NA	FALSE
chr19:37807762	1,49E-05	2,17E-02	0.138	*HKR1*	-1051	intron	0.078	-0.54	*ZNF527*	NA	NA	NA	FALSE
chr2:74643306	1,50E-05	2,18E-02	0.139	*DQ588163*	734	promoter	NA	-0.335	*INO80B*	NA	NA	NA	FALSE
chr15:70871818	1,56E-05	2,23E-02	-0.122	*UACA*	122803	intergenic	NA	NA	*NA*	NA	NA	NA	FALSE
chr5:78337539	1,58E-05	2,25E-02	-0.188	*DMGDH*	12633	intron	NA	0.404	*JMY*	NA	NA	NA	FALSE
chr11:67297539	1,59E-05	2,26E-02	-0.199	*CABP2*	-6641	intergenic	NA	-0.513	*RAD9A*	NA	NA	NA	FALSE
chr19:5131204	1,63E-05	2,30E-02	-0.154	*BC032415*	17313	exon	NA	-0.336	*KDM4B*	NA	NA	NA	FALSE
chr10:31040696	1,64E-05	2,30E-02	0.143	*AK302694*	59495	intergenic	NA	0.193	*ZNF438*	NA	NA	NA	FALSE
chr3:50231090	1,67E-05	2,32E-02	0.133	*GNAT1*	2049	exon	NA	-0.53	*RASSF1*	NA	NA	NA	FALSE
chr4:132897092	1,68E-05	2,32E-02	-0.121	*BC131768*	247841	intergenic	NA	NA	*NA*	NA	NA	NA	FALSE
chr16:32289963	1,68E-05	2,32E-02	-0.122	*LOC390705*	11340	intergenic	NA	NA	*NA*	NA	NA	NA	FALSE
chr12:281357	1,76E-05	2,40E-02	-0.145	*LOC574538*	-23026	intron	NA	0.226	*CCDC77*	NA	NA	NA	FALSE
chr6:163570179	1,86E-05	2,50E-02	-0.294	*AK296276*	42662	intron	NA	NA	*NA*	NA	NA	NA	FALSE
chr10:110075780	1,88E-05	2,50E-02	-0.143	*7SK*	625289	intergenic	NA	NA	*NA*	NA	NA	NA	FALSE
chr11:67351952	1,92E-05	2,53E-02	-0.104	*GSTP1*	888	promoter	-0.567	-0.567	*GSTP1*	NA	NA	NA	FALSE
chr18:709266	1,99E-05	2,59E-02	-0.121	*ENOSF1*	3252	intron	0.049	0.049	*ENOSF1*	NA	NA	NA	FALSE

chr9:137673849	2,01E-05	2,60E-02	-0.31	*MIR3689C*	67367	intron	NA	0.202	*FCN1*	NA	NA	NA	FALSE
chr14:65542732	2,05E-05	2,64E-02	0.172	*LOC100506321*	-13904	intron	NA	-0.375	*RP11-840I19.*	NA	NA	NA	FALSE
chr14:19888778	2,07E-05	2,64E-02	-0.133	*LINC00516*	-5591	intron	NA	NA	*NA*	NA	NA	NA	FALSE
chr1:54869222	2,17E-05	2,71E-02	0.171	*SSBP3*	2847	intron	0.051	0.205	*SSBP3-AS1*	NA	NA	NA	FALSE
chr7:76129418	2,18E-05	2,71E-02	-0.186	*DTX2*	301	promoter	-0.36	-0.491	*UPK3B*	NA	NA	NA	FALSE
chr22:49447747	2,16E-05	2,71E-02	0.229	*LOC100128946*	185167	intergenic	NA	NA	*NA*	NA	NA	NA	FALSE
chr10:102467237	2,21E-05	2,74E-02	-0.116	*PAX2*	-38231	intergenic	NA	-0.338	*SEC31B*	NA	NA	NA	FALSE
chr19:19778825	2,23E-05	2,75E-02	0.122	*ZNF101*	-838	promoter	-0.104	-0.415	*ZNF506*	NA	NA	NA	FALSE
chr8:41188402	2,27E-05	2,76E-02	-0.132	*SFRP1*	-21413	intergenic	NA	0.453	*GOLGA7*	NA	NA	NA	FALSE
chr1:7843461	2,30E-05	2,77E-02	0.102	*PER3*	-919	promoter	-0.301	-0.301	*PER3*	NA	NA	NA	FALSE
chr10:82131783	2,39E-05	2,83E-02	0.425	*DYDC2*	15261	intergenic	NA	0.118	*TSPAN14*	NA	NA	NA	FALSE
chr16:88453894	2,40E-05	2,83E-02	0.198	*ZNF469*	-39985	intergenic	NA	-0.149	*ZC3H18*	NA	NA	NA	FALSE
chr7:1371590	2,42E-05	2,84E-02	-0.124	*UNCX*	98938	intergenic	NA	-0.36	*INTS1*	NA	NA	NA	FALSE
chr9:137674085	2,45E-05	2,84E-02	-0.125	*MIR3689C*	67131	intron	NA	-0.047	*FCN1*	NA	NA	NA	FALSE
chr5:26326478	2,61E-05	2,98E-02	0.116	*TRNA_Lys*	-127868	intergenic	NA	NA	*NA*	NA	NA	NA	FALSE
chr19:22700771	2,65E-05	3,01E-02	0.166	*LOC440518*	-78288	intergenic	NA	NA	*NA*	NA	NA	NA	FALSE
chr1:43814632	2,69E-05	3,03E-02	-0.175	*CDC20*	-9994	exon	NA	0.147	*MED8*	NA	NA	NA	FALSE
chr2:130794732	2,71E-05	3,05E-02	-0.204	*LOC440905*	-5242	intron	NA	0.241	*MZT2B*	NA	NA	NA	FALSE
chr12:32626644	2,80E-05	3,10E-02	-0.35	*FGD4*	-12262	intergenic	NA	-0.223	*DNM1L*	NA	NA	NA	FALSE
chr2:204103125	3,05E-05	3,18E-02	-0.121	*CYP20A1*	-39	promoter	-0.031	0.045	*ABI2*	NA	NA	NA	FALSE
chr1:178456070	3,11E-05	3,22E-02	0.114	*TEX35*	-26142	intergenic	NA	-0.1	*RP11-428K3.1*	NA	NA	NA	FALSE
chr7:76129273	3,28E-05	3,34E-02	-0.174	*DTX2*	156	promoter	-0.501	-0.536	*UPK3B*	NA	NA	NA	FALSE
chr8:11086991	3,68E-05	3,57E-02	-0.186	*XKR6*	-28117	intergenic	NA	-0.052	*AF131216.6*	NA	NA	NA	FALSE
chr2:1817426	4,12E-05	3,80E-02	0.203	*MYT1L*	29233	intron	NA	-0.476	*PXDN*	NA	NA	NA	FALSE
chr8:120951820	4,12E-05	3,80E-02	0.175	*DEPTOR*	65922	intron	NA	-0.011	*TAF2*	NA	NA	NA	FALSE
chr7:128580230	4,14E-05	3,82E-02	0.216	*IRF5*	-542	promoter	-0.169	-0.319	*TNPO3*	NA	NA	NA	FALSE
chr1:178456064	4,19E-05	3,83E-02	0.121	*TEX35*	-26148	intergenic	NA	-0.059	*RALGPS2*	NA	NA	NA	FALSE
chr4:961370	4,18E-05	3,83E-02	0.156	*DGKQ*	5979	exon	0.438	0.438	*DGKQ*	NA	NA	NA	FALSE
chr2:73496134	4,29E-05	3,84E-02	-0.105	*FBXO41*	715	promoter	0.016	-0.266	*ALMS1-IT1*	NA	NA	NA	FALSE
chr12:118421892	4,56E-05	4,02E-02	0.269	*KSR2*	-15865	intergenic	NA	-0.278	*RFC5*	NA	NA	NA	FALSE
chr22:26323697	4,61E-05	4,03E-02	-0.149	*MYO18B*	-27448	intron	NA	NA	*NA*	NA	NA	NA	FALSE
chr19:1097295	4,77E-05	4,07E-02	-0.342	*POLR2E*	-1905	intergenic	0.152	0.406	*GPX4*	NA	NA	NA	FALSE
chr5:179193600	4,95E-05	4,17E-02	-0.197	*LTC4S*	-27386	exon	NA	-0.548	*RUFY1*	NA	NA	NA	FALSE
chr11:68782089	5,24E-05	4,36E-02	-0.11	*MRGPRF*	-1240	intron	NA	-0.219	*TPCN2*	NA	NA	NA	FALSE
chr16:67997921	5,24E-05	4,36E-02	-0.187	*SLC12A4*	48	promoter	-0.08	-0.257	*DUS2*	NA	NA	NA	TRUE
chr6:121069653	5,26E-05	4,36E-02	-0.118	*C6orf170*	488556	intergenic	NA	NA	*NA*	NA	NA	NA	FALSE
chr7:75280121	5,32E-05	4,40E-02	-0.117	*HIP1*	88163	intron	NA	0.273	*RHBDD2*	NA	NA	NA	FALSE
chr22:25233624	5,51E-05	4,49E-02	0.164	*SGSM1*	-5803	intron	NA	-0.212	*NA*	NA	NA	NA	FALSE
chr22:44529998	5,58E-05	4,53E-02	0.131	*TRNA_SeC*	-16539	intron	NA	0.249	*SAMM50*	NA	NA	NA	FALSE
chr1:21380201	6,06E-05	4,74E-02	0.208	*EIF4G3*	-2715	intron	0.149	0.207	*RP5-1071N3.*	NA	NA	NA	FALSE
chr19:728131	6,06E-05	4,74E-02	-0.151	*PALM*	347	promoter	NA	-0.388	*POLRMT*	NA	NA	NA	FALSE
chr1:178456078	6,22E-05	4,77E-02	0.105	*TEX35*	-26134	intergenic	NA	-0.141	*RP11-428K3.1*	NA	NA	NA	FALSE
chr19:48000364	6,19E-05	4,77E-02	-0.122	*NAPA-AS1*	12827	intron	0.026	0.35	*GLTSCR2*	NA	NA	NA	TRUE

chr4:132897121	6,63E-05	4,93E-02	-0.106	*BC131768*	247870	intergenic	NA	NA	*NA*	NA	NA	NA	FALSE
chr2:131695535	6,79E-05	5,00E-02	-0.214	*ARHGEF4*	7012	intron	-0.052	0.6	*FAM168B*	NA	NA	NA	FALSE
chr3:171464959	7,44E-05	5,33E-02	-0.115	*PLD1*	63546	intron	NA	NA	*NA*	NA	NA	NA	FALSE
chr22:49447896	7,76E-05	5,45E-02	0.258	*LOC100128946*	185316	intergenic	NA	NA	*NA*	NA	NA	NA	FALSE
chr11:122427556	7,86E-05	5,46E-02	-0.202	*TRNA_Lys*	-3099	intergenic	NA	-0.059	*UBASH3B*	NA	NA	NA	FALSE
chr4:675792	7,92E-05	5,46E-02	-0.133	*MFSD7*	3099	exon	NA	-0.392	*ATP5I*	NA	NA	NA	FALSE
chr4:186318341	8,23E-05	5,63E-02	-0.151	*ANKRD37*	503	promoter	0.127	0.35	*SNX25*	NA	NA	NA	FALSE
chr6:9971838	8,50E-05	5,72E-02	-0.103	*OFCC1*	5995	intron	NA	NA	*NA*	NA	NA	NA	FALSE
chr13:48892838	8,78E-05	5,79E-02	-0.142	*RB1*	14957	intron	0.01	0.227	*ITM2B*	NA	NA	NA	FALSE
chr16:89050546	8,82E-05	5,80E-02	0.235	*CBFA2T3*	-7043	intergenic	NA	-0.294	*APRT*	NA	NA	NA	FALSE
chr22:49448504	8,87E-05	5,81E-02	0.114	*LOC100128946*	185924	intergenic	NA	NA	*NA*	NA	NA	NA	FALSE
chr4:961400	9,04E-05	5,84E-02	0.239	*DGKQ*	5949	exon	0.339	0.339	*DGKQ*	NA	NA	NA	FALSE
chr1:110939144	9,56E-05	5,95E-02	-0.211	*SLC16A4*	-5441	intergenic	NA	0.338	*RBM15*	NA	NA	NA	FALSE
chr3:171464839	9,40E-05	5,95E-02	-0.102	*PLD1*	63666	intron	NA	NA	*NA*	NA	NA	NA	FALSE
chr6:28459170	9,78E-05	6,03E-02	-0.102	*TRNA_Thr*	-2328	intergenic	NA	-0.237	*PGBD1*	NA	NA	NA	FALSE
chr7:22860983	9,83E-05	6,04E-02	-0.224	*TOMM7*	1489	intron	0.12	-0.287	*AC005682.5*	NA	NA	NA	FALSE
chr2:1817753	9,85E-05	6,05E-02	0.174	*MYT1L*	28906	intron	NA	-0.629	*PXDN*	NA	NA	NA	FALSE
chr10:132961346	9,90E-05	6,06E-02	-0.15	*TCERG1L*	97362	intron	NA	NA	*NA*	NA	NA	NA	FALSE
chr4:961408	9,96E-05	6,07E-02	0.291	*DGKQ*	5941	exon	0.276	0.276	*DGKQ*	NA	NA	NA	FALSE
chr12:127631111	0,00010069	6,12E-02	-0.154	*BC032874*	-86170	intergenic	NA	NA	*NA*	NA	NA	NA	FALSE
chr17:28563513	0,00010457	6,27E-02	0.158	*SLC6A4*	-528	promoter	NA	-0.218	*BLMH*	NA	NA	NA	FALSE
chr1:152161883	0,00010489	6,27E-02	0.114	*RPTN*	-30180	intergenic	NA	0.338	*S100A10*	NA	NA	NA	FALSE
chr3:196694374	0,00010563	6,30E-02	-0.246	*PIGZ*	1331	intron	NA	-0.153	*AC127904.2*	NA	NA	NA	FALSE
chr4:3835991	0,00010941	6,38E-02	-0.164	*ADRA2C*	67697	intergenic	NA	NA	*NA*	NA	NA	NA	FALSE
chr4:2062464	0,00010972	6,39E-02	-0.145	*NAT8L*	1227	intron	NA	-0.255	*LETM1*	NA	NA	NA	FALSE
chr8:99017504	0,00011092	6,40E-02	0.104	*SNORA72*	36942	intron	0.07	-0.208	*HRSP12*	NA	NA	NA	FALSE
chr7:95181231	0,00011299	6,44E-02	-0.142	*PDK4*	40842	intergenic	NA	0.045	*PON2*	NA	NA	NA	FALSE
chr11:472217	0,00011345	6,44E-02	0.107	*PTDSS2*	21939	intron	0.399	0.419	*RP11-326C3.1*	NA	NA	NA	FALSE
chr18:77398054	0,00011344	6,44E-02	0.102	*CTDP1*	-41747	intergenic	0.042	-0.184	*NFATC1*	NA	NA	NA	FALSE
chr1:96689954	0,00011943	6,66E-02	0.505	*7SK*	471770	intergenic	NA	NA	*NA*	NA	NA	NA	FALSE
chr7:69896013	0,000121	6,71E-02	0.17	*AUTS2*	-335235	intron	0.303	NA	*NA*	NA	NA	NA	FALSE
chr11:818429	0,00012181	6,74E-02	-0.128	*PNPLA2*	-472	promoter	-0.024	-0.488	*TALDO1*	NA	NA	NA	FALSE
chr1:62752894	0,00012368	6,79E-02	-0.248	*KANK4*	-14432	intron	NA	0.275	*USP1*	NA	NA	NA	FALSE
chr2:241143204	0,00012368	6,79E-02	-0.174	*OTOS*	-63132	intergenic	NA	0.167	*NDUFA10*	NA	NA	NA	FALSE
chr19:1047217	0,00012413	6,80E-02	0.266	*ABCA7*	4558	exon	-0.28	-0.458	*WDR18*	NA	NA	NA	FALSE
chr22:44530028	0,00013526	7,14E-02	0.196	*TRNA_SeC*	-16509	intron	NA	0.305	*PARVB*	NA	NA	NA	FALSE
chr9:137674016	0,0001358	7,15E-02	-0.216	*MIR3689C*	67200	intron	NA	-0.079	*FCN1*	NA	NA	NA	FALSE
chr5:1246448	0,00014109	7,30E-02	0.173	*SLC6A18*	20980	intergenic	NA	0.22	*CLPTM1L*	NA	NA	NA	FALSE
chr8:144659988	0,00014284	7,35E-02	0.104	*NAPRT1*	526	promoter	-0.6	-0.6	*NAPRT1*	NA	NA	NA	FALSE
chr1:42105342	0,00014554	7,44E-02	-0.171	*HIVEP3*	-54354	intron	0.124	NA	*NA*	NA	NA	NA	FALSE
chr1:181944035	0,00014563	7,44E-02	-0.158	*ZNF648*	83111	intergenic	NA	NA	*NA*	NA	NA	NA	FALSE
chr7:76129434	0,00014897	7,54E-02	-0.174	*DTX2*	317	promoter	-0.409	-0.514	*UPK3B*	NA	NA	NA	FALSE
chr2:47015505	0,00015579	7,73E-02	-0.27	*LOC388948*	-28302	intergenic	NA	-0.449	*RHOQ*	NA	NA	NA	FALSE

chr2:232348540	0,00015672	7,73E-02	0.139	NCL	-19336	intergenic	-0.177	-0.177	NCL	NA	NA	NA	FALSE
chr14:19889020	0,00015671	7,73E-02	-0.166	LINC00516	-5349	intron	NA	NA	NA	NA	NA	NA	FALSE
chr20:56247302	0,00015696	7,73E-02	-0.109	PMEPA1	18379	intron	0.161	-0.334	ZBP1	NA	NA	NA	TRUE
chr22:49447907	0,00015645	7,73E-02	0.294	LOC100128946	185327	intergenic	NA	NA	NA	NA	NA	NA	FALSE
chr21:46677404	0,00016064	7,83E-02	0.215	POFUT2	19922	intron	-0.078	-0.375	ADARB1	NA	NA	NA	FALSE
chr10:1416894	0,00016394	7,96E-02	-0.234	ADARB2-AS1	-151931	intron	NA	NA	NA	NA	NA	NA	FALSE
chr6:163570442	0,00016635	8,05E-02	-0.143	AK296276	42399	intron	NA	NA	NA	NA	NA	NA	FALSE
chr1:43425452	0,00017051	8,13E-02	0.164	SLC2A1	-606	promoter	-0.144	-0.309	EBNA1BP2	NA	NA	NA	FALSE
chr11:42895488	0,00017076	8,13E-02	0.308	HNRNPKP3	395432	intergenic	NA	NA	NA	NA	NA	NA	FALSE
chr10:70979777	0,00017152	8,13E-02	0.116	HKDC1	-282	promoter	0.173	-0.336	SRGN	NA	NA	NA	TRUE
chr16:32290010	0,00017305	8,16E-02	-0.108	LOC390705	11293	intergenic	NA	NA	NA	NA	NA	NA	FALSE
chr17:7758222	0,00017378	8,19E-02	-0.11	TMEM88	-162	promoter	-0.13	-0.394	SAT2	NA	NA	NA	FALSE
chr8:12401905	0,00017717	8,24E-02	-0.109	AX747586	-25074	intron	NA	0.183	LONRF1	NA	NA	NA	FALSE
chr18:76003069	0,00017741	8,24E-02	-0.225	SALL3	-737206	intergenic	NA	NA	NA	NA	NA	NA	FALSE
chr22:28200203	0,00017809	8,24E-02	0.104	MN1	-2718	intergenic	-0.069	-0.41	PITPNB	NA	NA	NA	FALSE
chr22:44529945	0,00017967	8,27E-02	0.146	TRNA_SeC	-16592	intron	NA	0.184	SAMM50	NA	NA	NA	FALSE
chr19:1047215	0,0001832	8,35E-02	0.233	ABCA7	4556	exon	-0.202	0.391	CNN2	NA	NA	NA	FALSE
chr2:74643321	0,00018422	8,36E-02	0.139	DQ588163	749	promoter	NA	0.266	MTHFD2	NA	NA	NA	FALSE
chr4:132896374	0,00018486	8,37E-02	-0.133	BC131768	247123	intergenic	NA	NA	NA	NA	NA	NA	TRUE
chr21:46714738	0,0001848	8,37E-02	-0.166	LOC642852	6759	exon	NA	-0.529	POFUT2	NA	NA	NA	FALSE
chr4:961402	0,00018569	8,37E-02	0.337	DGKQ	5947	exon	0.403	0.403	DGKQ	NA	NA	NA	FALSE
chr6:39798623	0,00018558	8,37E-02	0.133	DAAM2	-17719	intron	NA	NA	NA	NA	NA	NA	FALSE
chr20:25847039	0,00018541	8,37E-02	0.105	FAM182B	1748	intron	NA	0.204	ZNF337	NA	NA	NA	FALSE
chr2:150177111	0,00018618	8,37E-02	0.136	LYPD6	-9388	intergenic	NA	NA	NA	NA	NA	NA	FALSE
chr2:1817466	0,00018786	8,42E-02	0.269	MYT1L	29193	intron	NA	-0.695	PXDN	NA	NA	NA	FALSE
chr17:135250	0,00019037	8,48E-02	0.161	RPH3AL	42121	intron	NA	-0.178	FAM101B	NA	NA	NA	FALSE
chr7:1857068	0,00019087	8,49E-02	-0.118	MIR4655	26822	intron	NA	-0.051	MAD1L1	NA	NA	NA	FALSE
chr18:60278825	0,00019831	8,68E-02	0.127	DKFZp451A185	29789	intergenic	NA	0.238	ZCCHC2	NA	NA	NA	FALSE
chr8:94129854	0,00020777	8,91E-02	-0.152	C8orf87	49226	intergenic	NA	NA	NA	NA	NA	NA	FALSE
chr11:78614302	0,00021165	9,02E-02	0.139	ODZ4	59097	intron	NA	NA	NA	NA	NA	NA	FALSE
chr18:77398380	0,00022233	9,29E-02	0.167	CTDP1	-41421	intergenic	0.128	0.128	CTDP1	NA	NA	NA	FALSE
chr2:241484089	0,00022347	9,31E-02	-0.227	ANKMY1	13317	intron	-0.009	-0.5	RNPEPL1	NA	NA	NA	FALSE
chr7:128580153	0,00022653	9,39E-02	0.167	IRF5	-619	promoter	-0.25	-0.379	TNPO3	NA	NA	NA	FALSE
chr4:132897090	0,00022976	9,44E-02	-0.11	BC131768	247839	intergenic	NA	NA	NA	NA	NA	NA	FALSE
chr18:11972554	0,00023449	9,56E-02	0.119	IMPA2	-8873	intergenic	0.008	-0.164	CHMP1B	NA	NA	NA	FALSE
chr11:400234	0,00023534	9,57E-02	0.12	PKP3	6019	intron	NA	-0.382	LRRC56	NA	NA	NA	FALSE
chr10:134648734	0,00023591	9,58E-02	0.195	NKX6-2	-49198	intron	NA	NA	NA	NA	NA	NA	FALSE
chr22:19751804	0,00024641	9,84E-02	0.124	TBX1	7580	exon	NA	0.184	C22orf29	NA	NA	NA	FALSE
chr2:232347976	0,00025181	9,92E-02	0.118	NCL	-18772	intergenic	-0.26	-0.26	NCL	NA	NA	NA	FALSE
chr15:79092841	0,00025189	9,92E-02	0.136	ADAMTS7	10933	exon	NA	-0.206	MORF4L1	NA	NA	NA	FALSE
chr19:5131447	0,00025155	9,92E-02	-0.131	BC032415	17556	exon	NA	0.319	ARRDC5	NA	NA	NA	FALSE

ESM Table 10. Differentially methylated CpGs identified in the CD8$^+$ T cell fraction between cases and controls prior to seroconversion

Methylation difference				Nearest gene			Methylation-expression correlation analysis			eQTM analysis			
CpG site	P value	FDR	Methylation difference	Nearest gene	Distance to nearest gene	Genomic part	Nearest gene correlation, Spearman rho	The highest observed correlation, Spearman rho	Correlating gene	CpG name	eQTM, FDR	Overall Z Score	CpG found on 450K
chr21:47285711	9,16E-22	4,78E-16	-0.427	*PCBP3*	15838	intron	-0.512	-0.512	*PCBP3*	NA	NA	NA	FALSE
chr17:71322457	1,92E-11	2,28E-06	0.548	*CDC42EP4*	-14315	intergenic	-0.04	0.516	*SLC39A11*	NA	NA	NA	FALSE
chr7:3169674	3,00E-10	2,31E-05	0.162	*BC038729*	44614	intergenic	NA	0.056	*CARD11*	NA	NA	NA	FALSE
chr19:12035022	3,10E-10	2,31E-05	0.115	*ZNF700*	-861	promoter	0.195	0.364	*ZNF439*	NA	NA	NA	FALSE
chr14:45343058	6,22E-10	4,33E-05	-0.259	*C14orf28*	-23449	intergenic	-0.065	-0.163	*FAM179B*	NA	NA	NA	FALSE
chr7:3169658	4,11E-09	2,38E-04	0.218	*BC038729*	44630	intergenic	NA	0.013	*CARD11*	NA	NA	NA	FALSE
chr21:47307753	6,48E-09	3,38E-04	-0.129	*PCBP3*	-8369	intron	-0.501	-0.501	*PCBP3*	NA	NA	NA	FALSE
chr14:65839593	7,98E-09	3,97E-04	0.155	*MIR4708*	-37693	intergenic	NA	-0.118	*FUT8*	NA	NA	NA	FALSE
chr16:58534501	8,70E-09	4,13E-04	0.135	*NDRG4*	456	promoter	NA	0.432	*CNOT1*	NA	NA	NA	FALSE
chr21:10597321	2,94E-08	1,09E-03	-0.174	*AK311573*	122	promoter	NA	NA	*NA*	NA	NA	NA	FALSE
chr9:137673887	3,92E-08	1,35E-03	-0.303	*MIR3689C*	67329	intron	NA	-0.267	*FCN1*	NA	NA	NA	FALSE
chr18:60278825	4,29E-08	1,35E-03	0.11	*DKFZp451A185*	29789	intergenic	NA	0.273	*ZCCHC2*	NA	NA	NA	FALSE
chr4:56023764	8,68E-08	2,45E-03	0.168	*KDR*	-32003	intergenic	NA	0.1	*TMEM165*	NA	NA	NA	FALSE
chr19:37807937	1,40E-07	3,61E-03	0.127	*HKR1*	-876	promoter	0.043	-0.326	*ZNF540*	NA	NA	NA	FALSE
chr7:64516680	1,77E-07	4,40E-03	-0.385	*CCT6P3*	17950	intron	0.002	0.09	*ERV3-1*	NA	NA	NA	FALSE
chr21:46714732	1,98E-07	4,59E-03	-0.145	*LOC642852*	6753	exon	NA	-0.286	*POFUT2*	NA	NA	NA	FALSE
chr7:95008632	2,76E-07	6,00E-03	0.126	*PON1*	17056	intron	NA	0.031	*PON2*	NA	NA	NA	FALSE
chr6:41720213	3,05E-07	6,25E-03	0.162	*PGC*	-5075	intergenic	NA	-0.308	*FRS3*	NA	NA	NA	FALSE
chr16:8960779	3,16E-07	6,34E-03	-0.155	*CARHSP1*	507	promoter	0.103	-0.245	*ABAT*	NA	NA	NA	FALSE
chr1:145385344	3,51E-07	6,42E-03	0.134	*TRNA_Asn*	-385	promoter	NA	-0.364	*PIAS3*	NA	NA	NA	FALSE
chr6:87680077	4,04E-07	6,81E-03	-0.13	*HTR1E*	33055	intron	NA	0.02	*ZNF292*	NA	NA	NA	FALSE
chr18:77454510	4,13E-07	6,85E-03	-0.284	*CTDP1*	13082	intron	-0.031	-0.031	*CTDP1*	NA	NA	NA	FALSE
chr14:24801107	4,51E-07	7,14E-03	-0.142	*ADCY4*	3171	exon	-0.049	0.357	*TINF2*	NA	NA	NA	FALSE
chr16:58534596	4,49E-07	7,14E-03	0.143	*NDRG4*	551	promoter	NA	-0.299	*GINS3*	NA	NA	NA	FALSE
chr11:42895488	5,21E-07	7,77E-03	0.289	*HNRNPKP3*	395432	intergenic	NA	NA	*NA*	NA	NA	NA	FALSE
chr6:53069739	5,61E-07	8,02E-03	-0.185	*GCM1*	-56116	intergenic	NA	0.388	*RN7SK*	NA	NA	NA	FALSE
chr13:20528307	7,23E-07	9,93E-03	-0.357	*ZMYM2*	-4503	intergenic	-0.006	0.243	*PSPC1*	NA	NA	NA	FALSE
chr21:47307758	8,33E-07	1,07E-02	-0.118	*PCBP3*	-8364	intron	-0.421	-0.421	*PCBP3*	NA	NA	NA	FALSE
chr22:50473537	8,97E-07	1,13E-02	-0.105	*IL17REL*	-22483	intergenic	NA	-0.372	*RP3-402G11.25*	NA	NA	NA	FALSE
chr4:132897258	9,74E-07	1,16E-02	-0.11	*BC131768*	248007	intergenic	NA	NA	*NA*	NA	NA	NA	FALSE
chr4:8890318	9,90E-07	1,16E-02	0.121	*HMX1*	-16776	intergenic	NA	NA	*NA*	NA	NA	NA	FALSE
chr5:270983	1,12E-06	1,27E-02	0.159	*PDCD6*	-753	promoter	0.081	0.424	*CTD-2083E4.4*	NA	NA	NA	FALSE
chr5:80529135	1,30E-06	1,37E-02	-0.195	*CKMT2*	-4	promoter	NA	0.318	*ZCCHC9*	NA	NA	NA	TRUE
chr11:3662811	1,32E-06	1,38E-02	-0.149	*ART5*	675	promoter	NA	-0.314	*PGAP2*	NA	NA	NA	FALSE
chr4:3835991	1,38E-06	1,41E-02	-0.195	*ADRA2C*	67697	intergenic	NA	NA	*NA*	NA	NA	NA	FALSE
chr1:12184835	1,55E-06	1,44E-02	-0.23	*TNFRSF8*	-1123	intron	NA	-0.192	*TNFRSF1B*	NA	NA	NA	FALSE
chr2:155146645	1,55E-06	1,44E-02	0.149	*GALNT13*	44322	intron	NA	NA	*NA*	NA	NA	NA	FALSE
chr17:80272875	1,53E-06	1,44E-02	0.168	*CD7*	2606	exon	-0.13	-0.187	*SLC16A3*	NA	NA	NA	FALSE
chr4:39569133	1,58E-06	1,45E-02	-0.409	*SMIM14*	16446	intron	NA	0.14	*RFC1*	NA	NA	NA	FALSE

chr19:53511567	1,78E-06	1,56E-02	-0.106	*AK127846*	804	promoter	NA	-0.307	*ZNF600*	NA	NA	NA	FALSE
chr22:39633525	1,90E-06	1,62E-02	0.114	*PDGFB*	3390	intron	NA	-0.484	*CBX7*	NA	NA	NA	FALSE
chr11:55640460	1,99E-06	1,63E-02	-0.182	*TRIM51*	-10313	intergenic	NA	NA	*NA*	NA	NA	NA	FALSE
chr14:34269955	2,18E-06	1,75E-02	-0.146	*EGLN3*	150078	exon	NA	NA	*NA*	NA	NA	NA	FALSE
chr6:44243633	2,38E-06	1,84E-02	0.202	*SPATS1*	2832	intron	NA	-0.153	*TMEM63B*	NA	NA	NA	FALSE
chr8:144660722	2,53E-06	1,87E-02	0.142	*NAPRT1*	-210	promoter	-0.543	-0.543	*NAPRT1*	NA	NA	NA	FALSE
chr19:37807898	2,54E-06	1,87E-02	0.107	*HKR1*	-915	promoter	0.036	-0.239	*ZNF540*	NA	NA	NA	FALSE
chr20:35001212	2,50E-06	1,87E-02	0.103	*DLGAP4*	5766	intron	-0.04	0.34	*C20orf24*	NA	NA	NA	FALSE
chr19:42021679	2,96E-06	2,04E-02	-0.281	*LOC100505495*	-15126	intergenic	NA	0.494	*ATP5SL*	NA	NA	NA	FALSE
chr22:49447970	3,10E-06	2,06E-02	0.247	*LOC100128946*	185390	intergenic	NA	NA	*NA*	NA	NA	NA	FALSE
chr9:137445381	3,51E-06	2,29E-02	-0.134	*COL5A1*	-88270	intergenic	NA	0.087	*RXRA*	NA	NA	NA	FALSE
chr20:48404038	3,71E-06	2,36E-02	-0.161	*SLC9A8*	-25212	intergenic	-0.007	0.289	*SNAI1*	NA	NA	NA	FALSE
chr4:81110000	3,78E-06	2,38E-02	-0.229	*PRDM8*	3578	intron	0.039	0.039	*PRDM8*	NA	NA	NA	FALSE
chr1:145385336	3,90E-06	2,42E-02	0.13	*TRNA_Asn*	-393	promoter	NA	-0.444	*PIAS3*	NA	NA	NA	FALSE
chr5:80529187	4,21E-06	2,51E-02	-0.115	*CKMT2*	50	promoter	NA	0.409	*ZCCHC9*	NA	NA	NA	TRUE
chr12:11812633	5,18E-06	2,90E-02	-0.147	*ETV6*	9847	intron	-0.471	-0.471	*ETV6*	NA	NA	NA	FALSE
chr16:21373131	5,22E-06	2,90E-02	0.166	*SNX29P1*	12449	intron	NA	-0.144	*NPIPB3*	NA	NA	NA	FALSE
chr19:23076831	5,21E-06	2,90E-02	0.133	*ZNF728*	93375	intergenic	NA	NA	*NA*	NA	NA	NA	FALSE
chr19:23076811	5,31E-06	2,92E-02	0.118	*ZNF728*	93395	intergenic	NA	NA	*NA*	NA	NA	NA	FALSE
chr8:1950591	5,43E-06	2,97E-02	0.201	*KBTBD11*	1234	exon	NA	0.376	*CTD-2336O2.1*	NA	NA	NA	FALSE
chr4:129135369	5,83E-06	3,12E-02	0.294	*LARP1B*	14777	intron	0.119	-0.416	*PGRMC2*	NA	NA	NA	FALSE
chr20:47013316	6,51E-06	3,40E-02	-0.219	*LINC00494*	18928	intergenic	NA	NA	*NA*	NA	NA	NA	FALSE
chr10:103885908	7,51E-06	3,73E-02	0.127	*LDB1*	-5699	intergenic	-0.016	-0.357	*HPS6*	NA	NA	NA	FALSE
chr16:1122053	7,70E-06	3,77E-02	0.111	*SSTR5*	-703	promoter	NA	-0.19	*CACNA1H*	NA	NA	NA	FALSE
chr1:19110630	8,46E-06	4,01E-02	0.176	*TAS1R2*	75526	intergenic	NA	0.06	*IFFO2*	NA	NA	NA	FALSE
chr16:1122013	8,52E-06	4,01E-02	0.105	*SSTR5*	-743	promoter	NA	-0.187	*UBE2I*	NA	NA	NA	FALSE
chr22:50473370	8,70E-06	4,03E-02	-0.13	*IL17REL*	-22316	intergenic	NA	0.333	*CITF22-1A6.3*	NA	NA	NA	FALSE
chr11:2121703	8,77E-06	4,04E-02	0.142	*INS-IGF2*	33202	intergenic	NA	-0.416	*TSPAN32*	NA	NA	NA	FALSE
chr9:140395291	8,94E-06	4,09E-02	0.176	*PNPLA7*	-13212	intron	0.152	-0.257	*NELFB*	NA	NA	NA	FALSE
chr9:89416194	9,31E-06	4,17E-02	0.115	*GAS1*	145911	intergenic	NA	NA	*NA*	NA	NA	NA	FALSE
chr5:177921624	9,68E-06	4,21E-02	-0.368	*COL23A1*	95933	intron	NA	0.274	*CLK4*	NA	NA	NA	FALSE
chr12:65285275	9,91E-06	4,24E-02	-0.32	*FLJ41278*	7723	intron	NA	-0.017	*GNS*	NA	NA	NA	FALSE
chr7:155211164	1,04E-05	4,34E-02	-0.198	*BC150495*	36395	intergenic	NA	0.411	*RBM33*	NA	NA	NA	FALSE
chr9:137673998	1,06E-05	4,41E-02	-0.352	*MIR3689C*	67218	intron	NA	-0.017	*FCN1*	NA	NA	NA	FALSE
chr2:242055173	1,12E-05	4,59E-02	-0.399	*MTERFD2*	-13427	intron	0.082	0.319	*PASK*	NA	NA	NA	FALSE
chr16:8960535	1,17E-05	4,76E-02	-0.152	*CARHSP1*	751	promoter	-0.023	-0.253	*ABAT*	NA	NA	NA	FALSE
chr1:121143463	1,21E-05	4,80E-02	-0.18	*SRGAP2D*	36313	intergenic	NA	-0.107	*NA*	NA	NA	NA	FALSE
chr17:21273093	1,23E-05	4,80E-02	-0.12	*KCNJ12*	-6606	intergenic	NA	0.206	*TMEM11*	NA	NA	NA	FALSE
chr3:194705950	1,35E-05	4,93E-02	-0.18	*XXYLT1*	136975	intergenic	-0.145	NA	*NA*	NA	NA	NA	FALSE
chr16:32290010	1,42E-05	5,14E-02	-0.127	*LOC390705*	11293	intergenic	NA	NA	*NA*	NA	NA	NA	FALSE
chr4:129135308	1,49E-05	5,26E-02	0.327	*LARP1B*	14716	intron	0.07	-0.373	*PGRMC2*	NA	NA	NA	FALSE
chr8:52321396	1,49E-05	5,26E-02	-0.196	*PXDNL*	726	promoter	NA	NA	*NA*	NA	NA	NA	FALSE
chr4:56023753	1,57E-05	5,34E-02	0.163	*KDR*	-31992	intergenic	NA	-0.088	*TMEM165*	NA	NA	NA	FALSE
chr1:55766588	1,64E-05	5,43E-02	-0.429	*MIR4422*	75276	intergenic	NA	0.026	*USP24*	NA	NA	NA	FALSE
chr7:99067371	1,67E-05	5,47E-02	0.189	*TRNA_Trp*	66	promoter	NA	-0.173	*ZKSCAN5*	NA	NA	NA	FALSE
chr10:70776965	1,68E-05	5,47E-02	0.295	*KIAA1279*	28490	intergenic	-0.029	-0.208	*VPS26A*	NA	NA	NA	FALSE
chr14:103603372	1,70E-05	5,50E-02	-0.375	*TNFAIP2*	4287	exon	-0.31	-0.31	*TNFAIP2*	NA	NA	NA	FALSE

chr19:53511717	1,75E-05	5,59E-02	-0.123	*AK127846*	954	promoter	NA	-0.47	*NDUFV2P1*	NA	NA	NA	FALSE
chr9:137673849	1,80E-05	5,65E-02	-0.289	*MIR3689C*	67367	intron	NA	-0.269	*FCN1*	NA	NA	NA	FALSE
chr4:1522024	1,81E-05	5,67E-02	0.235	*AX748388*	55943	intergenic	NA	0.396	*TMEM129*	NA	NA	NA	FALSE
chr21:19130093	1,87E-05	5,80E-02	0.181	*C21orf91-OT1*	34734	intergenic	NA	-0.177	*BTG3*	NA	NA	NA	FALSE
chr5:51168	1,90E-05	5,88E-02	-0.152	*PLEKHG4B*	-89205	intergenic	NA	0.315	*SDHA*	NA	NA	NA	FALSE
chr19:37807906	2,01E-05	6,05E-02	0.143	*HKR1*	-907	promoter	0.069	-0.267	*ZNF540*	NA	NA	NA	FALSE
chr16:14637963	2,05E-05	6,06E-02	-0.34	*PARN*	86166	intron	-0.183	-0.183	*PARN*	NA	NA	NA	FALSE
chr4:25828446	2,13E-05	6,19E-02	0.161	*SEL1L3*	-22073	intron	-0.26	-0.26	*SEL1L3*	NA	NA	NA	FALSE
chr4:81111507	2,15E-05	6,19E-02	-0.17	*PRDM8*	5085	intron	0.142	0.142	*PRDM8*	NA	NA	NA	FALSE
chr3:49570603	2,21E-05	6,26E-02	0.115	*BSN-AS2*	21197	exon	NA	-0.226	*RHOA*	NA	NA	NA	FALSE
chr10:105058506	2,33E-05	6,45E-02	-0.21	*INA*	21588	intergenic	NA	-0.103	*PDCD11*	NA	NA	NA	FALSE
chr16:9005505	2,38E-05	6,55E-02	-0.217	*USP7*	25039	intron	0.045	0.313	*RP11-473I1.9*	NA	NA	NA	FALSE
chr19:49000956	2,39E-05	6,55E-02	0.111	*LMTK3*	15491	exon	0.006	-0.325	*CA11*	NA	NA	NA	FALSE
chr6:163570410	2,41E-05	6,56E-02	-0.202	*AK296276*	42431	intron	NA	NA	*NA*	NA	NA	NA	FALSE
chr4:56023797	2,43E-05	6,57E-02	0.122	*KDR*	-32036	intergenic	NA	-0.129	*TMEM165*	NA	NA	NA	FALSE
chr20:61642359	2,47E-05	6,65E-02	0.17	*LOC63930*	1626	intron	NA	-0.414	*YTHDF1*	NA	NA	NA	FALSE
chr13:108449714	2,48E-05	6,66E-02	0.142	*BC043519*	38086	intron	NA	NA	*NA*	NA	NA	NA	FALSE
chr2:3454241	2,51E-05	6,73E-02	0.155	*TRAPPC12*	62852	intron	0.039	0.303	*TSSC1*	NA	NA	NA	FALSE
chr4:81109946	2,61E-05	6,97E-02	-0.185	*PRDM8*	3524	intron	0.067	0.067	*PRDM8*	NA	NA	NA	FALSE
chr8:144788313	2,75E-05	7,21E-02	0.108	*CCDC166*	1967	intron	NA	-0.354	*BREA2*	NA	NA	NA	FALSE
chr2:237085537	2,84E-05	7,31E-02	0.156	*GBX2*	-8886	intergenic	NA	NA	*NA*	NA	NA	NA	FALSE
chr6:2972312	2,88E-05	7,31E-02	-0.127	*SERPINB6*	88	promoter	0.384	0.384	*SERPINB6*	NA	NA	NA	FALSE
chr22:50472562	2,90E-05	7,33E-02	-0.171	*IL17REL*	-21508	intergenic	NA	-0.227	*RP3-402G11.25*	NA	NA	NA	FALSE
chr15:29684327	3,05E-05	7,55E-02	0.147	*NDNL2*	-122308	intron	-0.093	-0.093	*NDNL2*	NA	NA	NA	FALSE
chr4:56023751	3,15E-05	7,64E-02	0.195	*KDR*	-31990	intergenic	NA	-0.08	*TMEM165*	NA	NA	NA	TRUE
chr7:151137499	3,17E-05	7,64E-02	-0.172	*CRYGN*	-401	promoter	NA	-0.28	*WDR86-AS1*	NA	NA	NA	FALSE
chr4:1522095	3,38E-05	7,82E-02	0.295	*AX748388*	55872	intergenic	NA	0.487	*UVSSA*	NA	NA	NA	FALSE
chr4:8891755	3,41E-05	7,85E-02	0.144	*HMX1*	-18213	intergenic	NA	NA	*NA*	NA	NA	NA	FALSE
chr6:53069733	3,47E-05	7,95E-02	-0.208	*GCM1*	-56110	intergenic	NA	0.45	*RN7SK*	NA	NA	NA	FALSE
chr8:1950540	3,59E-05	8,08E-02	0.145	*KBTBD11*	1183	exon	NA	0.171	*CLN8*	NA	NA	NA	FALSE
chr15:101661802	3,57E-05	8,08E-02	-0.215	*CHSY1*	66465	intergenic	0.165	0.258	*VIMP*	NA	NA	NA	FALSE
chr4:132897056	3,81E-05	8,34E-02	-0.275	*BC131768*	247805	intergenic	NA	NA	*NA*	NA	NA	NA	FALSE
chr10:134597708	3,86E-05	8,40E-02	0.122	*NKX6-2*	1830	intergenic	NA	0.019	*INPP5A*	NA	NA	NA	FALSE
chr2:129160452	3,92E-05	8,47E-02	-0.192	*HS6ST1*	-84282	intergenic	-0.128	-0.128	*HS6ST1*	NA	NA	NA	FALSE
chr5:126111684	3,95E-05	8,48E-02	-0.146	*LMNB1*	-631	promoter	-0.153	0.249	*PHAX*	NA	NA	NA	FALSE
chr16:121845	3,95E-05	8,48E-02	-0.31	*RHBDF1*	785	promoter	NA	-0.426	*SNRNP25*	NA	NA	NA	FALSE
chr20:3733249	4,06E-05	8,59E-02	-0.172	*C20orf27*	15204	exon	0.152	0.372	*AP5S1*	NA	NA	NA	FALSE
chr16:433927	4,30E-05	8,77E-02	-0.204	*LOC100134368*	1688	intron	NA	-0.484	*TMEM8A*	NA	NA	NA	FALSE
chr11:96144166	4,37E-05	8,77E-02	-0.224	*JRKL*	21010	intergenic	NA	-0.169	*CCDC82*	NA	NA	NA	FALSE
chr16:8960813	4,40E-05	8,77E-02	-0.15	*CARHSP1*	473	promoter	0.002	-0.196	*NA*	NA	NA	NA	FALSE
chr3:96205331	4,44E-05	8,80E-02	-0.23	*Mir_548*	-179709	intergenic	NA	NA	*NA*	NA	NA	NA	FALSE
chr7:155211169	4,45E-05	8,81E-02	-0.202	*BC150495*	36400	intergenic	NA	0.26	*INSIG1*	NA	NA	NA	FALSE
chr22:49588344	4,61E-05	8,95E-02	-0.155	*LOC100128946*	325764	intergenic	NA	NA	*NA*	NA	NA	NA	FALSE
chr3:194705962	4,69E-05	9,01E-02	-0.135	*XXYLT1*	136963	intergenic	0.183	NA	*NA*	NA	NA	NA	FALSE
chr19:1047251	4,70E-05	9,01E-02	0.177	*ABCA7*	4592	exon	-0.087	0.3	*CIRBP-AS1*	NA	NA	NA	FALSE
chr3:128151227	4,81E-05	9,11E-02	-0.171	*DNAJB8*	30862	intergenic	NA	0.346	*RPN1*	NA	NA	NA	FALSE
chr11:55640449	4,83E-05	9,13E-02	-0.148	*TRIM51*	-10324	intergenic	NA	NA	*NA*	NA	NA	NA	FALSE

chr14:34269943	4,86E-05	9,15E-02	-0.193	*EGLN3*	150090	exon	NA	NA	*NA*	NA	NA	NA	FALSE
chr10:114335940	4,93E-05	9,20E-02	0.165	*MIR4295*	-57989	intron	NA	0.205	*VTI1A*	NA	NA	NA	FALSE
chr4:123286282	5,02E-05	9,30E-02	-0.161	*ADAD1*	-13839	intergenic	NA	0.175	*KIAA1109*	NA	NA	NA	FALSE
chr17:72916411	5,06E-05	9,32E-02	0.114	*USH1G*	2941	exon	NA	0.339	*KCTD2*	NA	NA	NA	FALSE
chr6:117536160	5,15E-05	9,37E-02	-0.351	*VGLL2*	-50561	intergenic	NA	NA	*NA*	NA	NA	NA	FALSE
chr19:40726376	5,38E-05	9,64E-02	0.175	*TTC9B*	-2071	intergenic	NA	0.325	*MAP3K10*	NA	NA	NA	FALSE
chr20:2690396	5,50E-05	9,77E-02	-0.187	*EBF4*	16874	intron	-0.152	0.488	*PCED1A*	NA	NA	NA	FALSE
chr3:13275522	5,53E-05	9,80E-02	-0.155	*NUP210*	120673	intergenic	-0.13	0.176	*HDAC11*	NA	NA	NA	FALSE
chr9:137674085	5,57E-05	9,85E-02	-0.13	*MIR3689C*	67131	intron	NA	-0.425	*FCN1*	NA	NA	NA	FALSE
chr20:4180020	5,73E-05	9,94E-02	-0.178	*LOC728228*	6285	intergenic	NA	0.301	*RNF24*	NA	NA	NA	FALSE
chr21:47307825	5,84E-05	9,98E-02	-0.142	*PCBP3*	-8297	intron	-0.534	-0.534	*PCBP3*	NA	NA	NA	FALSE

ESM Table 11. Differentially methylated CpGs identified in the $CD4^{-}CD8^{-}$ cell fraction between cases and controls prior to seroconversion

Methylation difference				Nearest gene			Methylation-expression correlation analysis			eQTM analysis			
CpG site	P value	FDR	Methylation difference	Nearest gene	Distance to nearest gene	Genomic part	Nearest gene correlation, Spearman rho	The highest observed correlation, Spearman rho	Correlating gene	CpG name	eQTM, FDR	Overall Z Score	CpG found on 450K
chr11:134709660	5,41E-17	4,72E-11	0.228	*AK125040*	103823	intergenic	NA	NA	*NA*	NA	NA	NA	FALSE
chr2:113192534	6,45E-13	1,87E-07	0.132	*RGPD8*	-473	promoter	0.014	0.253	*TTL*	NA	NA	NA	FALSE
chr8:144788521	1,09E-12	2,37E-07	0.18	*CCDC166*	1759	intron	NA	0.328	*ZC3H3*	NA	NA	NA	FALSE
chr3:194706028	9,52E-12	1,38E-06	-0.135	*XXYLT1*	136897	intergenic	-0.263	NA	*NA*	NA	NA	NA	FALSE
chr6:163570666	5,53E-10	4,25E-05	-0.219	*AK296276*	42175	intron	NA	NA	*NA*	NA	NA	NA	FALSE
chr9:136063981	4,89E-10	4,25E-05	-0.173	*OBP2B*	20648	intergenic	NA	0.226	*SURF2*	NA	NA	NA	FALSE
chr2:176121849	6,44E-10	4,32E-05	-0.276	*ATP5G3*	-75360	intergenic	0.068	0.068	*ATP5G3*	NA	NA	NA	FALSE
chr1:3811367	9,27E-10	5,77E-05	0.238	*C1orf174*	5491	intron	0.116	0.558	*DFFB*	NA	NA	NA	FALSE
chr22:49447907	1,85E-09	1,08E-04	0.263	*LOC100128946*	185327	intergenic	NA	NA	*NA*	NA	NA	NA	FALSE
chr10:123909315	3,11E-09	1,70E-04	0.113	*TACC2*	-13790	intron	NA	-0.108	*PLEKHA1*	NA	NA	NA	FALSE
chr3:194705954	4,26E-09	2,19E-04	-0.133	*XXYLT1*	136971	intergenic	-0.122	NA	*NA*	NA	NA	NA	TRUE
chr11:65359968	6,92E-09	3,14E-04	-0.32	*KCNK7*	3500	exon	0.129	-0.545	*LTBP3*	NA	NA	NA	FALSE
chr9:136075476	1,36E-08	5,18E-04	0.191	*OBP2B*	9153	intergenic	NA	0.31	*SURF1*	NA	NA	NA	FALSE
chr9:137674085	1,43E-08	5,18E-04	-0.131	*MIR3689C*	67131	intron	NA	0.297	*FCN1*	NA	NA	NA	FALSE
chr22:49447894	1,99E-08	6,67E-04	0.217	*LOC100128946*	185314	intergenic	NA	NA	*NA*	NA	NA	NA	FALSE
chr4:8890312	2,46E-08	7,96E-04	0.177	*HMX1*	-16770	intergenic	NA	NA	*NA*	NA	NA	NA	FALSE
chr11:122427613	2,84E-08	8,84E-04	-0.252	*TRNA_Lys*	-3042	intergenic	NA	-0.117	*UBASH3B*	NA	NA	NA	FALSE
chr10:1416833	4,08E-08	1,19E-03	-0.242	*ADARB2-AS1*	-151992	intron	NA	NA	*NA*	NA	NA	NA	FALSE
chr11:122427583	4,40E-08	1,20E-03	-0.236	*TRNA_Lys*	-3072	intergenic	NA	-0.156	*UBASH3B*	NA	NA	NA	FALSE
chr11:122427610	5,13E-08	1,32E-03	-0.217	*TRNA_Lys*	-3045	intergenic	NA	-0.167	*UBASH3B*	NA	NA	NA	FALSE
chr16:32289963	6,46E-08	1,61E-03	-0.142	*LOC390705*	11340	intergenic	NA	NA	*NA*	NA	NA	NA	FALSE
chr11:122427556	8,65E-08	1,88E-03	-0.232	*TRNA_Lys*	-3099	intergenic	NA	-0.075	*UBASH3B*	NA	NA	NA	FALSE
chr11:122427599	8,82E-08	1,88E-03	-0.246	*TRNA_Lys*	-3056	intergenic	NA	-0.177	*UBASH3B*	NA	NA	NA	FALSE
chr2:113192563	9,66E-08	2,01E-03	0.146	*RGPD8*	-502	promoter	0.024	0.336	*TTL*	NA	NA	NA	FALSE
chr2:198768428	1,17E-07	2,33E-03	0.47	*PLCL1*	99004	intron	0.398	0.398	*PLCL1*	NA	NA	NA	FALSE
chr12:132699571	1,24E-07	2,40E-03	-0.12	*GALNT9*	-8999	intron	NA	0.416	*NOC4L*	NA	NA	NA	FALSE
chr1:43425490	1,92E-07	3,22E-03	0.142	*SLC2A1*	-644	promoter	-0.129	0.264	*ERMAP*	NA	NA	NA	FALSE
chr2:27038368	1,99E-07	3,27E-03	-0.21	*CENPA*	29488	intergenic	NA	0.365	*TMEM214*	NA	NA	NA	FALSE
chr11:132662819	2,14E-07	3,46E-03	-0.229	*OPCML*	150219	intron	NA	NA	*NA*	NA	NA	NA	FALSE
chr11:55640232	3,37E-07	5,16E-03	-0.197	*TRIM51*	-10541	intergenic	NA	NA	*NA*	NA	NA	NA	FALSE
chr6:109705383	3,55E-07	5,34E-03	-0.238	*CD164*	-1622	intergenic	0.07	-0.377	*RP11-425D10.10*	NA	NA	NA	FALSE
chr14:65542732	4,27E-07	5,91E-03	0.15	*LOC100506321*	-13904	intron	NA	-0.401	*RP11-840I19.3*	NA	NA	NA	FALSE
chr10:80504266	4,87E-07	6,44E-03	0.133	*AX747983*	259295	intergenic	NA	NA	*NA*	NA	NA	NA	FALSE
chr19:17930969	4,95E-07	6,44E-03	0.364	*INSL3*	1415	intron	0.213	0.319	*JAK3*	NA	NA	NA	FALSE
chr2:27038339	5,31E-07	6,68E-03	-0.217	*CENPA*	29459	intergenic	NA	0.326	*TMEM214*	NA	NA	NA	FALSE
chr5:1064074	5,81E-07	6,71E-03	0.122	*MIR4635*	-986	promoter	NA	0.227	*BRD9*	NA	NA	NA	FALSE
chr16:50876453	6,11E-07	6,71E-03	0.122	*CYLD*	54758	intergenic	0.029	-0.142	*SNX20*	NA	NA	NA	FALSE
chr11:59323373	6,30E-07	6,79E-03	0.113	*TRNA_Lys*	-529	promoter	NA	-0.277	*OSBP*	NA	NA	NA	FALSE
chr11:122427608	7,05E-07	7,20E-03	-0.19	*TRNA_Lys*	-3047	intergenic	NA	-0.146	*UBASH3B*	NA	NA	NA	FALSE
chr22:49447896	8,09E-07	7,67E-03	0.225	*LOC100128946*	185316	intergenic	NA	NA	*NA*	NA	NA	NA	FALSE

chr19:37463844	1,04E-06	9,32E-03	0.143	*ZNF568*	-271	promoter	0.098	-0.208	*ZNF345*	NA	NA	NA	FALSE
chr8:144660146	1,39E-06	1,10E-02	0.101	*NAPRT1*	368	promoter	-0.285	0.361	*ZNF623*	NA	NA	NA	FALSE
chr7:154684199	1,44E-06	1,13E-02	-0.147	*LOC100132707*	-36028	exon	NA	0.274	*PAXIP1OS*	NA	NA	NA	FALSE
chr11:122427568	1,52E-06	1,17E-02	-0.226	*TRNA_Lys*	-3087	intergenic	NA	-0.181	*UBASH3B*	NA	NA	NA	FALSE
chr11:122427550	1,61E-06	1,21E-02	-0.228	*TRNA_Lys*	-3105	intergenic	NA	-0.068	*UBASH3B*	NA	NA	NA	FALSE
chr6:163570725	1,84E-06	1,30E-02	-0.127	*AK296276*	42116	intron	NA	NA	*NA*	NA	NA	NA	TRUE
chr6:121069679	1,95E-06	1,36E-02	-0.152	*C6orf170*	488530	intergenic	NA	NA	*NA*	NA	NA	NA	FALSE
chr10:2964694	2,05E-06	1,41E-02	-0.121	*PFKP*	-145018	intergenic	-0.114	-0.114	*PFKP*	NA	NA	NA	FALSE
chr20:52556902	2,20E-06	1,47E-02	0.12	*BCAS1*	55835	intergenic	NA	NA	*NA*	NA	NA	NA	FALSE
chr19:20179097	2,37E-06	1,57E-02	-0.131	*ZNF90*	-9706	intergenic	NA	-0.21	*ZNF682*	NA	NA	NA	FALSE
chr18:77476221	2,64E-06	1,68E-02	0.125	*CTDP1*	34793	intron	0.191	0.191	*CTDP1*	NA	NA	NA	FALSE
chr5:110230057	3,03E-06	1,82E-02	-0.119	*SLC25A46*	155305	intergenic	-0.115	-0.115	*SLC25A46*	NA	NA	NA	FALSE
chr4:6550122	3,48E-06	2,01E-02	-0.248	*PPP2R2C*	7284	intron	NA	-0.306	*AC093323.3*	NA	NA	NA	FALSE
chr8:95962352	3,66E-06	2,05E-02	-0.129	*TP53INP1*	-738	promoter	-0.277	-0.277	*TP53INP1*	NA	NA	NA	TRUE
chr17:1479190	4,36E-06	2,29E-02	-0.163	*PITPNA*	-13081	intron	-0.08	0.339	*INPP5K*	NA	NA	NA	FALSE
chr7:154684290	4,86E-06	2,44E-02	-0.176	*LOC100132707*	-35937	exon	NA	-0.163	*PAXIP1-AS1*	NA	NA	NA	FALSE
chr11:122427585	4,87E-06	2,44E-02	-0.186	*TRNA_Lys*	-3070	intergenic	NA	-0.087	*UBASH3B*	NA	NA	NA	FALSE
chr10:46097162	5,20E-06	2,54E-02	-0.116	*MARCH8*	-6809	intergenic	0.076	0.432	*ALOX5*	NA	NA	NA	FALSE
chr16:49569399	5,99E-06	2,74E-02	0.175	*ZNF423*	128738	intron	NA	NA	*NA*	NA	NA	NA	FALSE
chr2:27038329	6,17E-06	2,80E-02	-0.217	*CENPA*	29449	intergenic	NA	0.39	*TMEM214*	NA	NA	NA	FALSE
chr22:49447865	6,30E-06	2,82E-02	0.283	*LOC100128946*	185285	intergenic	NA	NA	*NA*	NA	NA	NA	FALSE
chr10:1416828	7,16E-06	2,98E-02	-0.283	*ADARB2-AS1*	-151997	intron	NA	NA	*NA*	NA	NA	NA	FALSE
chr16:1480869	7,38E-06	3,04E-02	0.135	*C16orf91*	-1525	intergenic	-0.06	0.611	*TSR3*	NA	NA	NA	TRUE
chr2:232061966	7,47E-06	3,06E-02	0.3	*ARMC9*	-1328	intergenic	NA	-0.186	*AC017104.6*	NA	NA	NA	FALSE
chr18:11972539	7,53E-06	3,07E-02	0.157	*IMPA2*	-8888	intergenic	0.123	-0.172	*MPPE1*	NA	NA	NA	FALSE
chr7:45854635	7,78E-06	3,16E-02	0.243	*DQ583079*	4347	intergenic	NA	0.347	*RP11-638I8.1*	NA	NA	NA	FALSE
chr2:107200953	7,89E-06	3,19E-02	-0.232	*RGPD3*	-116153	intergenic	NA	NA	*NA*	NA	NA	NA	FALSE
chr1:2785745	8,54E-06	3,33E-02	-0.127	*TTC34*	-79516	intergenic	NA	NA	*NA*	NA	NA	NA	FALSE
chr20:61642401	8,55E-06	3,33E-02	0.125	*LOC63930*	1668	intron	NA	0.483	*OGFR*	NA	NA	NA	FALSE
chr19:37807945	8,70E-06	3,37E-02	0.112	*HKR1*	-868	promoter	-0.162	-0.462	*ZNF570*	NA	NA	NA	FALSE
chr1:172419808	9,17E-06	3,41E-02	0.161	*PIGC*	-6579	intron	-0.278	-0.278	*PIGC*	NA	NA	NA	FALSE
chr9:136075132	9,36E-06	3,43E-02	0.133	*OBP2B*	9497	intergenic	NA	-0.352	*SURF2*	NA	NA	NA	FALSE
chr19:13337161	9,34E-06	3,43E-02	-0.144	*CACNA1A*	31205	intron	NA	-0.153	*NFIX*	NA	NA	NA	FALSE
chr7:98029163	9,54E-06	3,47E-02	-0.16	*BAIAP2L1*	1265	intron	0.242	-0.349	*TECPR1*	NA	NA	NA	FALSE
chr11:62370238	9,98E-06	3,54E-02	-0.137	*MTA2*	-927	promoter	0.281	-0.427	*INTS5*	NA	NA	NA	FALSE
chr12:12225262	1,02E-05	3,56E-02	-0.182	*BCL2L14*	862	promoter	NA	NA	*NA*	NA	NA	NA	TRUE
chr11:42894941	1,04E-05	3,58E-02	0.309	*HNRNPKP3*	395979	intergenic	NA	NA	*NA*	NA	NA	NA	FALSE
chr2:184044615	1,11E-05	3,72E-02	-0.13	*NUP35*	55534	intergenic	0.003	0.003	*NUP35*	NA	NA	NA	FALSE
chr3:194705981	1,11E-05	3,72E-02	-0.125	*XXYLT1*	136944	intergenic	-0.041	NA	*NA*	NA	NA	NA	FALSE
chr20:46526153	1,11E-05	3,72E-02	-0.121	*BX648826*	-58890	intergenic	NA	0.307	*SULF2*	NA	NA	NA	FALSE
chr2:113192585	1,16E-05	3,81E-02	0.127	*RGPD8*	-524	promoter	-0.185	-0.214	*ZC3H6*	NA	NA	NA	FALSE
chr6:1599310	1,18E-05	3,83E-02	0.104	*FOXC1*	-11371	intergenic	NA	NA	*NA*	NA	NA	NA	FALSE
chr2:113192546	1,20E-05	3,87E-02	0.101	*RGPD8*	-485	promoter	-0.175	-0.192	*ZC3H8*	NA	NA	NA	FALSE
chr9:136075222	1,23E-05	3,94E-02	0.168	*OBP2B*	9407	intergenic	NA	-0.256	*SURF2*	NA	NA	NA	FALSE
chr2:113192477	1,32E-05	4,13E-02	0.162	*RGPD8*	-416	promoter	-0.016	0.417	*TTL*	NA	NA	NA	FALSE
chr1:172419795	1,52E-05	4,44E-02	0.149	*PIGC*	-6566	intron	-0.13	0.172	*FASLG*	NA	NA	NA	FALSE
chr7:154684272	1,58E-05	4,55E-02	-0.179	*LOC100132707*	-35955	exon	NA	-0.062	*PAXIP1*	NA	NA	NA	FALSE

chr7:73648167	1,61E-05	4,57E-02	-0.149	*RFC2*	20572	intron	0.016	-0.313	*LAT2*	NA	NA	NA	FALSE
chr14:24801107	1,61E-05	4,57E-02	-0.143	*ADCY4*	3171	exon	0.021	0.438	*PSME2*	NA	NA	NA	FALSE
chr16:87709152	1,65E-05	4,60E-02	0.37	*FLJ00104*	26179	intron	NA	0.15	*KLHDC4*	NA	NA	NA	FALSE
chr9:136075488	1,83E-05	4,98E-02	0.139	*OBP2B*	9141	intergenic	NA	0.328	*MED22*	NA	NA	NA	FALSE
chr11:122427545	1,86E-05	5,01E-02	-0.2	*TRNA_Lys*	-3110	intergenic	NA	-0.117	*UBASH3B*	NA	NA	NA	FALSE
chr9:136075435	1,93E-05	5,14E-02	0.124	*OBP2B*	9194	intergenic	NA	0.378	*GBGT1*	NA	NA	NA	FALSE
chr2:27038396	2,02E-05	5,23E-02	-0.195	*CENPA*	29516	intergenic	NA	0.374	*TMEM214*	NA	NA	NA	FALSE
chr15:93277269	2,05E-05	5,30E-02	0.102	*FAM174B*	36	promoter	NA	-0.217	*CHD2*	NA	NA	NA	FALSE
chr2:176121784	2,15E-05	5,51E-02	-0.142	*ATP5G3*	-75295	intergenic	-0.043	-0.099	*ATF2*	NA	NA	NA	FALSE
chr20:62797416	2,29E-05	5,68E-02	0.114	*MYT1*	1591	intron	NA	0.206	*RGS19*	NA	NA	NA	FALSE
chr10:46097166	2,30E-05	5,70E-02	-0.106	*Mar.08*	-6813	intergenic	0.205	0.372	*ALOX5*	NA	NA	NA	FALSE
chr18:77476218	2,32E-05	5,71E-02	0.105	*CTDP1*	34790	intron	0.151	0.151	*CTDP1*	NA	NA	NA	FALSE
chr19:22700806	2,42E-05	5,86E-02	0.233	*LOC440518*	-78253	intergenic	NA	NA	*NA*	NA	NA	NA	FALSE
chr19:37807898	2,42E-05	5,86E-02	0.166	*HKR1*	-915	promoter	-0.095	-0.425	*ZNF570*	NA	NA	NA	FALSE
chr9:136063893	2,44E-05	5,89E-02	-0.107	*OBP2B*	20736	intergenic	NA	0.137	*GBGT1*	NA	NA	NA	FALSE
chr3:118892467	2,53E-05	5,96E-02	0.219	*UPK1B*	44	promoter	NA	0.261	*ARHGAP31*	NA	NA	NA	FALSE
chr6:39280817	2,51E-05	5,96E-02	-0.187	*KCNK17*	1421	intron	NA	-0.364	*SAYSD1*	NA	NA	NA	FALSE
chr8:72754307	2,50E-05	5,96E-02	0.108	*LOC100132891*	-1051	exon	NA	0.42	*MSC*	NA	NA	NA	FALSE
chr5:173991159	2,58E-05	6,03E-02	0.138	*MSX2*	-160416	intergenic	NA	NA	*NA*	NA	NA	NA	FALSE
chr19:56769427	2,65E-05	6,09E-02	-0.175	*ZSCAN5A*	-29769	intron	NA	-0.338	*ZNF444*	NA	NA	NA	FALSE
chr21:46677471	2,65E-05	6,09E-02	0.523	*POFUT2*	19855	intron	0.279	0.279	*POFUT2*	NA	NA	NA	FALSE
chr6:1599373	2,72E-05	6,18E-02	0.149	*FOXC1*	-11308	intergenic	NA	NA	*NA*	NA	NA	NA	FALSE
chr4:81110085	2,88E-05	6,40E-02	-0.265	*PRDM8*	3663	intron	-0.002	-0.116	*ANTXR2*	NA	NA	NA	FALSE
chr8:54935437	2,92E-05	6,40E-02	0.108	*TCEA1*	-430	promoter	-0.275	-0.275	*TCEA1*	NA	NA	NA	FALSE
chr10:1416879	2,87E-05	6,40E-02	-0.197	*ADARB2-AS1*	-151946	intron	NA	NA	*NA*	NA	NA	NA	FALSE
chr17:4805462	3,03E-05	6,52E-02	0.146	*CHRNE*	908	promoter	-0.138	0.389	*GP1BA*	NA	NA	NA	FALSE
chr10:1416819	3,09E-05	6,60E-02	-0.294	*ADARB2-AS1*	-152006	intron	NA	NA	*NA*	NA	NA	NA	FALSE
chr11:122427594	3,08E-05	6,60E-02	-0.225	*TRNA_Lys*	-3061	intergenic	NA	-0.126	*UBASH3B*	NA	NA	NA	FALSE
chr8:54935522	3,12E-05	6,63E-02	0.148	*TCEA1*	-515	promoter	0.157	0.214	*ATP6V1H*	NA	NA	NA	FALSE
chr12:32626634	3,26E-05	6,79E-02	-0.411	*FGD4*	-12272	intergenic	0.05	0.229	*DNM1L*	NA	NA	NA	FALSE
chr16:9091790	3,50E-05	7,06E-02	-0.111	*USP7*	-34450	intergenic	0.11	0.325	*PMM2*	NA	NA	NA	FALSE
chr6:1594275	3,75E-05	7,39E-02	0.121	*FOXC1*	-16406	intergenic	NA	NA	*NA*	NA	NA	NA	TRUE
chr11:59323375	3,73E-05	7,39E-02	0.117	*TRNA_Lys*	-527	promoter	NA	-0.151	*STX3*	NA	NA	NA	FALSE
chr11:122427537	3,86E-05	7,56E-02	-0.196	*TRNA_Lys*	-3118	intergenic	NA	-0.052	*UBASH3B*	NA	NA	NA	FALSE
chr14:19888831	3,92E-05	7,61E-02	-0.127	*LINC00516*	-5538	intron	NA	NA	*NA*	NA	NA	NA	FALSE
chr21:46714850	4,03E-05	7,77E-02	-0.153	*LOC642852*	6871	exon	NA	-0.347	*POFUT2*	NA	NA	NA	FALSE
chr4:81111299	4,08E-05	7,82E-02	-0.234	*PRDM8*	4877	intron	0.037	-0.15	*ANTXR2*	NA	NA	NA	FALSE
chr5:271209	4,12E-05	7,86E-02	0.109	*PDCD6*	-527	promoter	-0.031	0.195	*CTD-2228K2.5*	NA	NA	NA	FALSE
chr3:193678373	4,18E-05	7,94E-02	-0.108	*DPPA2P3*	33655	intron	NA	0.215	*HES1*	NA	NA	NA	FALSE
chr10:1416906	4,26E-05	8,00E-02	-0.214	*ADARB2-AS1*	-151919	intron	NA	NA	*NA*	NA	NA	NA	FALSE
chr10:132893061	4,40E-05	8,09E-02	0.26	*MIR378C*	-132131	intron	NA	NA	*NA*	NA	NA	NA	FALSE
chr19:1047251	4,93E-05	8,68E-02	0.171	*ABCA7*	4592	exon	0.077	0.501	*ATP5D*	NA	NA	NA	FALSE
chr19:22800813	4,94E-05	8,68E-02	0.101	*BC030765*	5961	intron	NA	NA	*NA*	NA	NA	NA	FALSE
chr7:64540916	4,97E-05	8,70E-02	-0.159	*BC044608*	723	promoter	NA	0.215	*ERV3-1*	NA	NA	NA	FALSE
chr9:137673947	5,04E-05	8,72E-02	-0.262	*MIR3689C*	67269	intron	NA	0.49	*FCN1*	NA	NA	NA	FALSE
chr14:105636665	5,10E-05	8,78E-02	-0.226	*JAG2*	-1505	intergenic	0.174	0.262	*NUDT14*	NA	NA	NA	FALSE
chr11:122427606	5,26E-05	8,99E-02	-0.241	*TRNA_Lys*	-3049	intergenic	NA	-0.118	*UBASH3B*	NA	NA	NA	FALSE

chr19:50962487	5,31E-05	9,00E-02	0.121	*EMC10*	-17170	exon	0.012	0.527	*KCNC3*	NA	NA	NA	FALSE
chr3:96495764	5,74E-05	9,29E-02	-0.151	*EPHA6*	-37661	intergenic	NA	NA	*NA*	NA	NA	NA	FALSE
chr16:1310093	5,81E-05	9,32E-02	0.12	*TPSD1*	3578	intergenic	NA	0.372	*GNPTG*	NA	NA	NA	FALSE
chr19:22800795	5,93E-05	9,44E-02	0.111	*BC030765*	5979	intron	NA	NA	*NA*	NA	NA	NA	FALSE
chr2:113190197	6,01E-05	9,55E-02	0.132	*RGPD8*	1026	intron	-0.151	0.152	*TTL*	NA	NA	NA	TRUE
chr19:50962477	6,22E-05	9,76E-02	0.137	*EMC10*	-17180	exon	-0.1	-0.275	*MYBPC2*	NA	NA	NA	FALSE
chr12:6252166	6,54E-05	9,92E-02	-0.193	*VWF*	-18331	intergenic	NA	-0.165	*CD9*	NA	NA	NA	FALSE
chr22:38714287	6,47E-05	9,92E-02	0.112	*CSNK1E*	-199	promoter	0.041	0.041	*CSNK1E*	cg06748147	0.042906225	-3.660446	TRUE
chr2:107200920	6,62E-05	9,98E-02	-0.185	*RGPD3*	-116120	intergenic	NA	NA	*NA*	NA	NA	NA	FALSE

ESM Table 12. Differentially methylated regions identified in the CD4$^+$ T cell fraction between cases and controls prior to seroconversion

Differentially methylated region							Nearest gene			GeneHancer analysis	
DMR	Area size, bp	Number of significant CpGs	Number of positive CpGs	Number of negative CpGs	Best P value within DMR	Best FDR within FDR	Nearest gene	Distance to nearest gene	Genomic part	GeneHancer database	Genes possibly regulated by enhacer or promoter
chr19:18118304-chr19:18118337	33	2	0	2	4,22E-22	1,64E-16	*ARRDC2*	-673	promoter		
chr7:3169658-chr7:3169674	16	2	2	0	4,76E-13	4,62E-08	*BC038729*	44630	intergenic		
chr2:27038313-chr2:27038448	135	8	0	8	1,50E-11	6,25E-07	*CENPA*	29459	intergenic		
chr19:37807627-chr19:37807945	318	19	19	0	1,61E-11	6,25E-07	*HKR1*	-907	promoter	promoter	*ZNF527, ZNF793, LINC01535, HKR1, ENSG00000267682*
chr2:113192114-chr2:113192585	471	8	8	0	6,97E-11	2,25E-06	*RGPD8*	-100	promoter		
chr22:39633525-chr22:39633541	16	2	2	0	1,04E-08	1,49E-04	*PDGFB*	3390	intron	promoter	*CBX7, SYNGR1, PDGFB*
chr19:58571181-chr19:58571458	277	9	9	0	1,36E-08	1,75E-04	*ZNF135*	836	promoter	promoter	*ZNF135*
chr5:270987-chr5:271170	183	3	3	0	2,15E-08	2,53E-04	*PDCD6*	-749	promoter	promoter	*HRAT5*
chr18:77397918-chr18:77398519	601	21	21	0	3,06E-08	3,27E-04	*CTDP1*	-41603	intergenic		
chr1:228658942-chr1:228659210	268	12	12	0	3,33E-08	3,40E-04	*Histone3*	-7151	intergenic		
chr7:22860953-chr7:22861119	166	13	0	13	3,68E-08	3,56E-04	*TOMM7*	1487	intron	promoter	*TOMM7*
chr21:47307634-chr21:47307825	191	9	0	9	7,69E-08	6,48E-04	*PCBP3*	-8310	intron	enhancer	*PCBP3*
chr14:103227394-chr14:103227476	82	3	0	3	1,19E-07	9,42E-04	*TRAF3*	-16422	intergenic		
chr2:130794723-chr2:130794741	18	5	0	5	1,29E-07	1,00E-03	*LOC440905*	-5242	intron		
chr12:32626634-chr12:32626743	109	3	0	3	1,42E-07	1,08E-03	*FGD4*	-12272	intergenic		
chr9:136074985-chr9:136075539	554	13	13	0	1,56E-07	1,10E-03	*OBP2B*	9194	intergenic		
chr22:49447601-chr22:49448582	981	23	23	0	2,27E-07	1,33E-03	*LOC100128946*	185327	intergenic		
chr9:137673743-chr9:137674085	342	15	0	15	4,05E-07	2,15E-03	*MIR3689C*	67367	intron		
chr19:2561771-chr19:2561793	22	2	2	0	4,47E-07	2,34E-03	*BC022568*	82057	intron		
chr10:71801791-chr10:71802193	402	4	0	4	5,16E-07	2,48E-03	*H2AFY2*	-10200	intergenic		
chr4:186808118-chr4:186808125	7	2	0	2	5,70E-07	2,66E-03	*SORBS2*	69753	intron		
chr15:93277255-chr15:93277269	14	2	2	0	7,08E-07	3,19E-03	*FAM174B*	50	promoter	promoter	*FAM174B*
chr5:43000441-chr5:43000725	284	4	0	4	8,34E-07	3,52E-03	*AK056817*	-7183	intergenic		
chr13:48892744-chr13:48892861	117	5	0	5	1,09E-06	4,22E-03	*RB1*	14946	intron		
chr4:961370-chr4:962384	1014	18	18	0	1,29E-06	4,84E-03	*DGKQ*	5947	exon		
chr1:30911320-chr1:30911425	105	2	0	2	1,30E-06	4,84E-03	*MATN1-AS1*	-280194	intergenic		
chr4:132896374-chr4:132897121	747	6	0	6	1,44E-06	5,18E-03	*BC131768*	247749	intergenic		
chr3:171464839-chr3:171464959	120	3	0	3	1,62E-06	5,65E-03	*PLD1*	63546	intron		
chr19:1336229-chr19:1336307	78	4	4	0	1,77E-06	5,90E-03	*MUM1*	-18675	intergenic		
chr10:74082048-chr10:74082090	42	4	4	0	1,84E-06	6,01E-03	*DNAJB12*	32852	intergenic	enhancer	*DDIT4, DNAJB12*
chr7:64540896-chr7:64541058	162	3	0	3	2,66E-06	7,76E-03	*BC044608*	743	promoter	enhancer	*GTF2IP14*
chr22:44529945-chr22:44530101	156	6	6	0	2,95E-06	8,40E-03	*TRNA_SeC*	-16509	intron		
chr3:196694114-chr3:196694374	260	9	0	9	3,06E-06	8,65E-03	*PIGZ*	1341	intron		
chr11:122427377-chr11:122427617	240	13	0	13	3,37E-06	9,14E-03	*TRNA_Lys*	-3049	intergenic		
chr8:95962244-chr8:95962383	139	7	0	7	5,46E-06	1,22E-02	*TP53INP1*	-656	promoter	promoter	*NDUFAF6, TP53INP1*
chr6:28459146-chr6:28459170	24	2	0	2	5,92E-06	1,30E-02	*TRNA_Thr*	-2304	intergenic		
chr13:21900270-chr13:21900480	210	9	0	9	6,16E-06	1,34E-02	*MIPEPP3*	28098	intron	enhancer	*GRK6P1, LINC00539*
chr20:56247302-chr20:56247315	13	2	0	2	6,78E-06	1,43E-02	*PMEPA1*	18379	intron	enhancer	*PMEPA1*
chr16:89050366-chr16:89050672	306	7	7	0	6,80E-06	1,43E-02	*CBFA2T3*	-7089	intergenic	enhancer	*CBFA2T3*
chr10:15038010-chr10:15038132	122	4	4	0	8,55E-06	1,67E-02	*DCLRE1C*	25731	intron		
chr18:60278762-chr18:60278915	153	6	6	0	8,66E-06	1,67E-02	*DKFZp451A185*	29853	intergenic		
chr15:101661777-chr15:101662001	224	8	0	8	9,37E-06	1,68E-02	*CHSY1*	66461	intergenic	enhancer	*CHSY1*

chr17:48586117-chr17:48586230	113	7	7	0	9,66E-06	1,71E-02	*MYCBPAP*	487	promoter	promoter	*EPN3, MYCBPAP*
chr22:17198946-chr22:17199120	174	9	0	9	1,09E-05	1,80E-02	*BC038197*	30355	intergenic		
chr19:58521594-chr19:58521598	4	2	2	0	1,30E-05	1,99E-02	*ZNF606*	-6881	intergenic	promoter	*LOC100128398*
chr12:133304192-chr12:133304215	23	2	0	2	1,38E-05	2,08E-02	*ANKLE2*	7084	intron		
chr2:74643130-chr2:74643331	201	12	12	0	1,50E-05	2,18E-02	*DQ588163*	679	promoter		
chr11:67297539-chr11:67297564	25	2	0	2	1,59E-05	2,26E-02	*CABP2*	-6641	intergenic		
chr19:5131177-chr19:5131521	344	8	0	8	1,63E-05	2,30E-02	*BC032415*	17592	exon		
chr10:31040591-chr10:31040696	105	4	4	0	1,64E-05	2,30E-02	*AK302694*	59491	intergenic		
chr3:50231090-chr3:50231414	324	5	5	0	1,67E-05	2,32E-02	*GNAT1*	2079	intron	enhancer	*RBM6, HYAL3*
chr16:32289963-chr16:32290029	66	3	0	3	1,68E-05	2,32E-02	*LOC390705*	11340	intergenic		
chr6:163570179-chr6:163570616	437	17	0	17	1,86E-05	2,50E-02	*AK296276*	42662	intron		
chr10:110075471-chr10:110075815	344	14	0	14	1,88E-05	2,50E-02	*7SK*	625445	intergenic		
chr11:67351930-chr11:67351952	22	5	0	5	1,92E-05	2,53E-02	*GSTP1*	873	promoter	promoter	*NDUFV1, NUDT8, ENSG00000255119*
chr14:19888706-chr14:19889043	337	5	0	5	2,07E-05	2,64E-02	*LINC00516*	-5349	intron		
chr7:76129266-chr7:76129437	171	9	0	9	2,18E-05	2,71E-02	*DTX2*	301	promoter		
chr1:7843461-chr1:7843473	12	2	2	0	2,30E-05	2,77E-02	*PER3*	-907	promoter	promoter	*PER3*
chr16:88453817-chr16:88453894	77	5	5	0	2,40E-05	2,83E-02	*ZNF469*	-39985	intergenic	enhancer	*ZNF469*
chr7:1371569-chr7:1371590	21	2	0	2	2,42E-05	2,84E-02	*UNCX*	98917	intergenic		
chr19:22700771-chr19:22700778	7	2	2	0	2,65E-05	3,01E-02	*LOC440518*	-78288	intergenic		
chr1:43814495-chr1:43814672	177	14	0	14	2,69E-05	3,03E-02	*CDC20*	-10049	exon		
chr1:178456064-chr1:178456108	44	5	5	0	3,11E-05	3,22E-02	*TEX35*	-26148	intergenic		
chr2:1817284-chr2:1817793	509	23	23	0	4,12E-05	3,80E-02	*MYT1L*	29193	intron		
**chr7:128580072-chr7:128580230**	158	5	5	0	4,14E-05	3,82E-02	*IRF5*	-542	promoter	promoter	*IRF5*
chr2:73496134-chr2:73496203	69	5	0	5	4,29E-05	3,84E-02	*FBXO41*	655	promoter		
chr22:26323528-chr22:26323710	182	4	0	4	4,61E-05	4,03E-02	*MYO18B*	-27435	intron		
chr11:68782089-chr11:68782202	113	4	0	4	5,24E-05	4,36E-02	*MRGPRF*	-1266	intron	promoter	*MRPL21, IGHMBP2, MRGPRF*
chr16:67997858-chr16:67997921	63	2	0	2	5,24E-05	4,36E-02	*SLC12A4*	48	promoter	promoter	*PRMT7*
chr6:121069653-chr6:121069703	50	4	0	4	5,26E-05	4,36E-02	*C6orf170*	488540	intergenic		
chr7:75280121-chr7:75280192	71	3	0	3	5,32E-05	4,40E-02	*HIP1*	88134	intron	enhancer	*HIP1*
chr22:25233624-chr22:25233695	71	4	4	0	5,51E-05	4,49E-02	*SGSM1*	-5803	intron		
chr19:728066-chr19:728176	110	6	0	6	6,06E-05	4,74E-02	*PALM*	351	promoter		
chr19:48000357-chr19:48000450	93	3	0	3	6,19E-05	4,77E-02	*NAPA-AS1*	12820	intron		
chr4:675645-chr4:675792	147	12	0	12	7,92E-05	5,46E-02	*MFSD7*	3246	intron		
chr4:186318341-chr4:186318356	15	3	0	3	8,23E-05	5,63E-02	*ANKRD37*	510	promoter	promoter	*LRP2BP, UFSP2, C4orf47*
chr10:132961346-chr10:132961395	49	2	0	2	9,90E-05	6,06E-02	*TCERG1L*	97313	exon		
chr12:127631022-chr12:127631189	167	10	0	10	1,01E-04	6,12E-02	*BC032874*	-86124	intergenic	promoter	*ENSG00000256001*
chr1:152161878-chr1:152161927	49	5	5	0	1,05E-04	6,27E-02	*RPTN*	-30175	intergenic	promoter	*FLG-AS1*
chr4:3835894-chr4:3835991	97	3	0	3	1,09E-04	6,38E-02	*ADRA2C*	67685	intergenic		
chr4:2062464-chr4:2062937	473	12	0	12	1,10E-04	6,39E-02	*NAT8L*	1651	exon		
chr1:62752777-chr1:62752894	117	2	0	2	1,24E-04	6,79E-02	*KANK4*	-14432	intron	enhancer	*KANK4*
chr19:1047141-chr19:1047348	207	8	8	0	1,24E-04	6,80E-02	*ABCA7*	4558	exon		
chr5:1246414-chr5:1246456	42	4	4	0	1,41E-04	7,30E-02	*SLC6A18*	20980	intergenic		
chr8:144659799-chr8:144659988	189	18	18	0	1,43E-04	7,35E-02	*NAPRT1*	549	promoter	promoter	*NAPRT*
chr2:232347976-chr2:232348566	590	14	14	0	1,57E-04	7,73E-02	*NCL*	-19318	intergenic		
chr21:46677404-chr21:46677632	228	9	9	0	1,61E-04	7,83E-02	*POFUT2*	19846	intron		
chr10:1416791-chr10:1416983	192	11	0	11	1,64E-04	7,96E-02	*ADARB2-AS1*	-151997	intron		
chr1:43425385-chr1:43425514	129	8	8	0	1,71E-04	8,13E-02	*SLC2A1*	-606	promoter	promoter	*SVBP, SLC2A1, SLC2A1-AS1*
chr18:76002967-chr18:76003069	102	3	0	3	1,77E-04	8,24E-02	*SALL3*	-737206	intergenic		

chr21:46714650-chr21:46714850	200	10	0	10	1,85E-04	8,37E-02	*LOC642852*	6797	exon	enhancer	*POFUT2, LINC00205*
chr2:150177044-chr2:150177357	313	4	4	0	1,86E-04	8,37E-02	*LYPD6*	-9142	intergenic		
chr17:135245-chr17:135302	57	5	5	0	1,90E-04	8,48E-02	*RPH3AL*	42121	intron		
chr11:78614302-chr11:78614306	4	2	2	0	2,12E-04	9,02E-02	*ODZ4*	59097	intron		
chr18:11972458-chr18:11972666	208	6	6	0	2,34E-04	9,56E-02	*IMPA2*	-8899	intergenic		
chr11:400130-chr11:400432	302	14	14	0	2,35E-04	9,57E-02	*PKP3*	6026	intron		
chr15:79092681-chr15:79092841	160	7	7	0	2,52E-04	9,92E-02	*ADAMTS7*	10938	exon		

ESM Table 13. Differentially methylated regions identified in the CD8$^+$ T cell fraction between cases and controls prior to seroconversion

Differentially methylated region							Nearest gene			GeneHancer analysis	
DMR	Area size, bp	Number of significant CpGs	Number of positive CpGs	Number of negative CpGs	Best P value within DMR	Best FDR within FDR	Nearest gene	Distance to nearest gene	Genomic part	GeneHancer database	Genes possibly regulated by enhacer or promoter
chr7:3169658-chr7:3169674	16	2	2	0	3,00E-10	2,31E-05	*BC038729*	44630	intergenic		
chr19:12035022-chr19:12035043	21	2	2	0	3,10E-10	2,31E-05	*ZNF700*	-840	promoter	promoter	*ZNF700, ZNF763, ENSG00000267274*
chr14:45343058-chr14:45343116	58	2	0	2	6,22E-10	4,33E-05	*C14orf28*	-23449	intergenic		
chr21:47307692-chr21:47307825	133	6	0	6	6,48E-09	3,38E-04	*PCBP3*	-8385	intron	enhancer	*PCBP3*
chr16:58534501-chr16:58534640	139	3	3	0	8,70E-09	4,13E-04	*NDRG4*	551	promoter	promoter	*NDRG4*
chr9:137673739-chr9:137675444	1705	28	0	28	3,92E-08	1,35E-03	*MIR3689C*	67218	intron		
chr18:60278578-chr18:60278825	247	5	5	0	4,29E-08	1,35E-03	*DKFZp451A185*	29589	intergenic		
chr4:56023751-chr4:56023797	46	4	4	0	8,68E-08	2,45E-03	*KDR*	-31990	intergenic		
chr19:37807633-chr19:37807939	306	13	13	0	1,40E-07	3,61E-03	*HKR1*	-1025	intron	promoter	*ZNF527, LINC01535, ZNF793, HKR1, ENSG00000267682*
chr21:46714714-chr21:46714891	177	7	0	7	1,98E-07	4,59E-03	*LOC642852*	6912	exon	enhancer	*LINC00205*
chr16:8960438-chr16:8960833	395	18	0	18	3,16E-07	6,34E-03	*CARHSP1*	772	promoter	promoter	*PMM2, CARHSP1, ENSG00000260276*
chr1:145385184-chr1:145385411	227	15	14	1	3,51E-07	6,42E-03	*TRNA_Asn*	-430	promoter		
chr14:24801052-chr14:24801180	128	5	0	5	4,51E-07	7,14E-03	*ADCY4*	3171	exon		
chr11:42895488-chr11:42895608	120	3	3	0	5,21E-07	7,77E-03	*HNRNPKP3*	395432	intergenic		
chr6:53069701-chr6:53069739	38	3	0	3	5,61E-07	8,02E-03	*GCM1*	-56110	intergenic		
chr22:50472562-chr22:50473702	1140	6	0	6	8,97E-07	1,13E-02	*IL17REL*	-21508	intergenic		
chr4:132896405-chr4:132897280	875	23	0	23	9,74E-07	1,16E-02	*BC131768*	247839	intergenic		
chr4:8890256-chr4:8891755	1499	8	8	0	9,90E-07	1,16E-02	*HMX1*	-16714	intergenic		
chr5:270919-chr5:271170	251	8	8	0	1,12E-06	1,27E-02	*PDCD6*	-682	promoter	promoter	*HRAT5*
chr5:80529067-chr5:80529208	141	8	0	8	1,30E-06	1,37E-02	*CKMT2*	-72	promoter	promoter	*CKMT2-AS1*
chr11:3662811-chr11:3662967	156	2	0	2	1,32E-06	1,38E-02	*ART5*	675	promoter	promoter	*ART5*
chr4:3835938-chr4:3835991	53	3	0	3	1,38E-06	1,41E-02	*ADRA2C*	67685	intergenic		
chr17:80272853-chr17:80272875	22	2	2	0	1,53E-06	1,44E-02	*CD7*	2606	exon		
chr1:12184835-chr1:12184846	11	2	0	2	1,55E-06	1,44E-02	*TNFRSF8*	-1112	intron		
chr19:53511567-chr19:53511717	150	2	0	2	1,78E-06	1,56E-02	*AK127846*	954	promoter		
chr11:55640248-chr11:55640479	231	15	0	15	1,99E-06	1,63E-02	*TRIM51*	-10418	intergenic		
chr14:34269670-chr14:34270090	420	18	0	18	2,18E-06	1,75E-02	*EGLN3*	150027	exon		
chr6:44243475-chr6:44243633	158	7	7	0	2,38E-06	1,84E-02	*SPATS1*	2832	intron		
chr8:144660146-chr8:144660722	576	4	4	0	2,53E-06	1,87E-02	*NAPRT1*	-181	promoter	promoter	*NAPRT*
chr19:42021679-chr19:42021799	120	4	0	4	2,96E-06	2,04E-02	*LOC100505495*	-15126	intergenic		
chr22:49447609-chr22:49448436	827	22	22	0	3,10E-06	2,06E-02	*LOC100128946*	185390	intergenic		
chr4:81109888-chr4:81111756	1868	60	0	60	3,78E-06	2,38E-02	*PRDM8*	3915	intron		
chr19:23076700-chr19:23076831	131	3	3	0	5,21E-06	2,90E-02	*ZNF728*	93375	intergenic		
chr8:1950540-chr8:1950658	118	12	12	0	5,43E-06	2,97E-02	*KBTBD11*	1234	exon	enhancer	*KBTBD11*
chr4:129135308-chr4:129135369	61	2	2	0	5,83E-06	3,12E-02	*LARP1B*	14716	intron		
chr20:47013135-chr20:47013950	815	22	1	21	6,51E-06	3,40E-02	*LINC00494*	19361	intergenic		
chr16:1122013-chr16:1122053	40	4	4	0	7,70E-06	3,77E-02	*SSTR5*	-703	promoter		
chr1:19110630-chr1:19110910	280	5	5	0	8,46E-06	4,01E-02	*TAS1R2*	75526	intergenic		
chr9:140395198-chr9:140395357	159	7	7	0	8,94E-06	4,09E-02	*PNPLA7*	-13212	intron		
chr7:155211140-chr7:155211176	36	7	0	7	1,04E-05	4,34E-02	*BC150495*	36387	intergenic		
chr1:121143410-chr1:121143613	203	10	0	10	1,21E-05	4,80E-02	*SRGAP2D*	36313	intergenic		
chr3:194705950-chr3:194706107	157	5	0	5	1,35E-05	4,93E-02	*XXYLT1*	136975	intergenic		
chr16:32289928-chr16:32290380	452	10	0	10	1,42E-05	5,14E-02	*LOC390705*	11324	intergenic		
chr8:52321396-chr8:52321489	93	2	0	2	1,49E-05	5,26E-02	*PXDNL*	726	promoter		
chr7:99067328-chr7:99067371	43	4	4	0	1,67E-05	5,47E-02	*TRNA_Trp*	66	promoter		
chr10:70776965-chr10:70776986	21	2	2	0	1,68E-05	5,47E-02	*KIAA1279*	28511	intergenic		

chr14:103603202-chr14:103603554	352	10	0	10	1,70E-05	5,50E-02	*TNFAIP2*	4287	exon	enhancer	*NDUFB4P11*
chr4:1521934-chr4:1522400	466	24	24	0	1,81E-05	5,67E-02	*AX748388*	55872	intergenic		
chr5:51168-chr5:51230	62	3	0	3	1,90E-05	5,88E-02	*PLEKHG4B*	-89152	intergenic		
chr19:49000956-chr19:49001623	667	4	4	0	2,39E-05	6,55E-02	*LMTK3*	14824	exon		
chr6:163570179-chr6:163570776	597	34	0	34	2,41E-05	6,56E-02	*AK296276*	42574	intron		
chr20:61642309-chr20:61642387	78	5	5	0	2,47E-05	6,65E-02	*LOC63930*	1626	intron	enhancer	*BHLHE23, LINC01749*
chr8:144787910-chr8:144789427	1517	24	24	0	2,75E-05	7,21E-02	*CCDC166*	1666	intron		
chr6:2972312-chr6:2972326	14	3	0	3	2,88E-05	7,31E-02	*SERPINB6*	74	promoter	promoter	*SERPINB6*
chr15:101661802-chr15:101662001	199	8	0	8	3,57E-05	8,08E-02	*CHSY1*	66465	intergenic	enhancer	*CHSY1*
chr10:134597567-chr10:134597708	141	2	2	0	3,86E-05	8,40E-02	*NKX6-2*	1971	intergenic		
chr5:126111555-chr5:126111694	139	6	0	6	3,95E-05	8,48E-02	*LMNB1*	-631	promoter		
chr16:121845-chr16:121905	60	3	0	3	3,95E-05	8,48E-02	*RHBDF1*	785	promoter	promoter	*NPRL3, ENSG00000268836, MPG, RHBDF1*
chr20:3733245-chr20:3733249	4	2	0	2	4,06E-05	8,59E-02	*C20orf27*	15208	exon	enhancer	*HSPA12B, CDC25B*
chr16:433855-chr16:434103	248	8	0	8	4,30E-05	8,77E-02	*LOC100134368*	1864	intron	promoter	*TMEM8A, MRPL28*
chr11:96144166-chr11:96144326	160	2	0	2	4,37E-05	8,77E-02	*JRKL*	21010	intergenic		
chr22:49588344-chr22:49588480	136	6	0	6	4,61E-05	8,95E-02	*LOC100128946*	325900	intergenic		
chr19:1047141-chr19:1047391	250	15	15	0	4,70E-05	9,01E-02	*ABCA7*	4529	exon		
chr3:128151140-chr3:128151291	151	7	0	7	4,81E-05	9,11E-02	*DNAJB8*	30862	intergenic		
chr4:123286278-chr4:123286282	4	2	0	2	5,02E-05	9,30E-02	*ADAD1*	-13843	intergenic		
chr17:72916365-chr17:72916434	69	5	5	0	5,06E-05	9,32E-02	*USH1G*	2923	exon		
chr20:2690382-chr20:2690396	14	2	0	2	5,50E-05	9,77E-02	*EBF4*	16860	intron		
chr3:13275511-chr3:13275522	11	3	0	3	5,53E-05	9,80E-02	*NUP210*	120673	intergenic		

ESM Table 14. Differentially methylated regions identified in the CD4⁻CD8⁻ cell fraction between cases and controls prior to seroconversion

Differentially methylated region							Nearest gene			GeneHancer analysis	
DMR	Area size, bp	Number of significant CpGs	Number of positive CpGs	Number of negative CpGs	Best P value within DMR	Best FDR within FDR	Nearest gene	Distance to nearest gene	Genomic part	GeneHancer database	Genes possibly regulated by enhacer or promoter
chr2:113192187-chr2:113192585	398	9	9	0	6,45E-13	1,87E-07	*RGPD8*	-416	promoter		
chr8:144787766-chr8:144788824	1058	23	23	0	1,09E-12	2,37E-07	*CCDC166*	1666	intron		
chr3:194705950-chr3:194706146	196	9	0	9	9,52E-12	1,38E-06	*XXYLT1*	136975	intergenic		
chr9:136063893-chr9:136063981	88	2	0	2	4,89E-10	4,25E-05	*OBP2B*	20648	intergenic		
chr6:163570410-chr6:163570776	366	16	0	16	5,53E-10	4,25E-05	*AK296276*	42175	intron		
chr2:176121784-chr2:176121927	143	8	0	8	6,44E-10	4,32E-05	*ATP5G3*	-75360	intergenic		
chr22:49447601-chr22:49447913	312	13	13	0	1,85E-09	1,08E-04	*LOC100128946*	185265	intergenic		
chr10:123909315-chr10:123909514	199	2	2	0	3,11E-09	1,70E-04	*TACC2*	-13591	intron		
chr11:65359906-chr11:65360009	103	12	0	12	6,92E-09	3,14E-04	*KCNK7*	3500	exon	enhancer	*MAP3K11, EHBP1L1*
chr9:136075028-chr9:136075533	505	14	14	0	1,36E-08	5,18E-04	*OBP2B*	9153	intergenic		
chr9:137673849-chr9:137674085	236	14	0	14	1,43E-08	5,18E-04	*MIR3689C*	67265	intron		
chr4:8890133-chr4:8891755	1622	7	7	0	2,46E-08	7,96E-04	*HMX1*	-16770	intergenic		
chr11:122427537-chr11:122427617	80	14	0	14	2,84E-08	8,84E-04	*TRNA_Lys*	-3042	intergenic		
chr10:1416791-chr10:1416983	192	17	0	17	4,08E-08	1,19E-03	*ADARB2-AS1*	-152006	intron		
chr16:32289963-chr16:32290196	233	7	0	7	6,46E-08	1,61E-03	*LOC390705*	11107	intergenic		
chr1:43425385-chr1:43425514	129	7	7	0	1,92E-07	3,22E-03	*SLC2A1*	-633	promoter	promoter	*SVBP, SLC2A1, SLC2A1-AS1*
chr11:132662753-chr11:132662968	215	5	0	5	2,14E-07	3,46E-03	*OPCML*	150219	intron		
chr11:55640097-chr11:55640479	382	19	0	19	3,37E-07	5,16E-03	*TRIM51*	-10541	intergenic		
chr6:109705248-chr6:109705383	135	6	0	6	3,55E-07	5,34E-03	*CD164*	-1568	intergenic	promoter	*SMPD2, ZBTB24*
chr11:59323373-chr11:59323375	2	2	2	0	6,30E-07	6,79E-03	*TRNA_Lys*	-527	promoter	enhancer	*STX3*
chr19:37463627-chr19:37463867	240	6	6	0	1,04E-06	9,32E-03	*ZNF568*	-296	promoter	promoter	*ZNF383, ZNF527, ZNF568*
chr8:144659799-chr8:144660146	347	17	17	0	1,39E-06	1,10E-02	*NAPRT1*	494	promoter	promoter	*NAPRT*
chr7:154684101-chr7:154684455	354	12	0	12	1,44E-06	1,13E-02	*LOC100132707*	-35955	exon		
chr6:121069599-chr6:121069769	170	6	0	6	1,95E-06	1,36E-02	*C6orf170*	488506	intergenic		
chr10:2964694-chr10:2964704	10	2	0	2	2,05E-06	1,41E-02	*PFKP*	-145018	intergenic		
chr18:77476218-chr18:77476246	28	3	3	0	2,64E-06	1,68E-02	*CTDP1*	34793	intron		
chr5:110229890-chr5:110230059	169	5	0	5	3,03E-06	1,82E-02	*SLC25A46*	155138	intergenic		
chr8:95962270-chr8:95962352	82	3	0	3	3,66E-06	2,05E-02	*TP53INP1*	-673	promoter	promoter	*TP53INP1, NDUFAF6*
chr17:1479078-chr17:1479190	112	4	0	4	4,36E-06	2,29E-02	*PITPNA*	-13081	intron	enhancer	*SLC43A2*
chr10:46097162-chr10:46097166	4	3	0	3	5,20E-06	2,54E-02	*MARCH8*	-6811	intergenic		
chr2:107200907-chr2:107200963	56	9	0	9	7,89E-06	3,19E-02	*RGPD3*	-116153	intergenic		
chr20:61642298-chr20:61642401	103	6	6	0	8,55E-06	3,33E-02	*LOC63930*	1626	intron	enhancer	*BHLHE23, LINC01749*
chr19:37807567-chr19:37807945	378	22	22	0	8,70E-06	3,37E-02	*HKR1*	-1156	intron	promoter	*ZNF527, LINC01535, ZNF793, HKR1*
chr1:172419795-chr1:172419861	66	3	3	0	9,17E-06	3,41E-02	*PIGC*	-6579	intron	enhancer	*C1orf105, PIGC, SUCO*
chr7:98029126-chr7:98029163	37	2	0	2	9,54E-06	3,47E-02	*BAIAP2L1*	1265	intron	promoter	*BRI3, BAIAP2L1*
chr11:62370220-chr11:62370410	190	11	0	11	9,98E-06	3,54E-02	*MTA2*	-1099	intron	promoter	*EML3, GANAB, TUT1, MTA2*
chr12:12225214-chr12:12225285	71	6	0	6	1,02E-05	3,56E-02	*BCL2L14*	885	promoter	promoter	*BCL2L14*
chr20:46526131-chr20:46526153	22	2	0	2	1,11E-05	3,72E-02	*BX648826*	-58890	intergenic		
chr6:1599255-chr6:1599388	133	8	8	0	1,18E-05	3,83E-02	*FOXC1*	-11293	intergenic		
chr14:24801057-chr14:24801180	123	4	0	4	1,61E-05	4,57E-02	*ADCY4*	3171	exon		
chr16:87709144-chr16:87709241	97	4	4	0	1,65E-05	4,60E-02	*FLJ00104*	26179	intron		

chr19:22700742-chr19:22700806	64	3	3	0	2,42E-05	5,86E-02	*LOC440518*	-78253	intergenic		
chr8:72754075-chr8:72754314	239	7	7	0	2,50E-05	5,96E-02	*LOC100132891*	-1269	exon	promoter	*MSC*
chr6:39280817-chr6:39280861	44	4	0	4	2,51E-05	5,96E-02	*KCNK17*	1421	intron	enhancer	*KCNK17*
chr3:118892305-chr3:118892496	191	10	10	0	2,53E-05	5,96E-02	*UPK1B*	44	promoter		
chr19:56769408-chr19:56769430	22	3	0	3	2,65E-05	6,09E-02	*ZSCAN5A*	-29750	intron		
chr21:46675864-chr21:46677632	1768	27	27	0	2,65E-05	6,09E-02	*POFUT2*	19855	intron		
chr4:81109972-chr4:81111756	1784	51	0	51	2,88E-05	6,40E-02	*PRDM8*	3856	intron		
chr8:54935437-chr8:54935522	85	2	2	0	2,92E-05	6,40E-02	*TCEA1*	-515	promoter		
chr17:4805462-chr17:4805474	12	2	2	0	3,03E-05	6,52E-02	*CHRNE*	908	promoter	promoter	*CHRNE, C17orf107*
chr12:32626634-chr12:32626743	109	3	0	3	3,26E-05	6,79E-02	*FGD4*	-12272	intergenic		
chr6:1594275-chr6:1594451	176	3	3	0	3,75E-05	7,39E-02	*FOXC1*	-16406	intergenic		
chr14:19888706-chr14:19889624	918	19	0	19	3,92E-05	7,61E-02	*LINC00516*	-4792	intron		
chr21:46714714-chr21:46714850	136	3	0	3	4,03E-05	7,77E-02	*LOC642852*	6871	exon	enhancer	*LINC00205*
chr5:270919-chr5:271209	290	7	7	0	4,12E-05	7,86E-02	*PDCD6*	-682	promoter	promoter	*HRAT5*
chr3:193678336-chr3:193678373	37	2	0	2	4,18E-05	7,94E-02	*DPPA2P3*	33692	intron		
chr19:1047188-chr19:1047323	135	11	11	0	4,93E-05	8,68E-02	*ABCA7*	4605	exon		
chr19:22800715-chr19:22800880	165	4	4	0	4,94E-05	8,68E-02	*BC030765*	5979	intron		
chr7:64540916-chr7:64540960	44	3	0	3	4,97E-05	8,70E-02	*BC044608*	723	promoter	enhancer	*GTF2IP14*
chr14:105636575-chr14:105636711	136	5	0	5	5,10E-05	8,78E-02	*JAG2*	-1415	intergenic		
chr19:50962477-chr19:50962487	10	2	2	0	5,31E-05	9,00E-02	*EMC10*	-17180	exon		
chr3:96495764-chr3:96495824	60	5	0	5	5,74E-05	9,29E-02	*EPHA6*	-37653	intergenic		
chr2:113190019-chr2:113190239	220	9	9	0	6,01E-05	9,55E-02	*RGPD8*	1026	intron	promoter	*RGPD8*
